# *Xylaria* species associated with fallen leaves and petioles

**DOI:** 10.1186/s40529-023-00377-w

**Published:** 2023-07-14

**Authors:** Yu-Ming Ju, Huei-Mei Hsieh

**Affiliations:** Institute of Plant and Microbial Biology, Academia Sinica, Nankang, Taipei, 11529 Taiwan

**Keywords:** 1 newly combined name, 2 new replacement names, 3 unnamed species, 9 newly described species, Ascomycota, Caulicolous, Foliicolous, Taxonomy, Xylariaceae

## Abstract

**Background:**

*Xylaria* species growing on fallen leaves and petioles have not been treated systematically. One source of confusion in this group of *Xylaria* species has stemmed from *X. filiformis*, which is an ancient name published in 1805 as *Sphaeria filiformis* and has commonly labeled on specimen packets that contain leaf- and petiole-inhabiting *Xylaria* species. Here we clarified the identity of *X. filiformis* and distinguish it from the species that are easily confused with it, notably *X*. *simplicissima*, to which most specimens labeled as *X*. *filiformis* are referred. Our research also led us to encounter many other leaf- and petiole-inhabiting *Xylaria* species, prompting a comprehensive study of this group of fungi.

**Results:**

Forty-five foliicolous and caulicolous species of *Xylaria* were studied, including nine newly described species—*X*. *allima*, *X*. *appendiculatoides*, *X*. *hispidipes*, *X*. *minuscula*, *X*. *neblinensis*, *X. spiculaticlavata*, *X*. *vermiformis*, *X*. *vittatipiliformis*, and *X*. *vittiformis*; three unnamed species—*X*. sp. AR1741, *X*. sp. GS7461A, and *X*. sp. GS7461B; *X*. *simplicissima*, a name newly combined with *Xylaria* from *Rhizomorpha simplicissima*; and *X*. *noduliformis* and *X*. *imminuta*, which are two new replacement names, respectively, for *X*. *maitlandii* var. *nuda* and *X*. *hypsipoda* var. *microspora*. The 45 taxa can be classified into three groups by stromatal shape and conspicuousness of perithecial mounds on the stromatal surface: (i) the *X*. *filiformis* group contains 10 species, (ii) the *X*. *phyllocharis* group contains 19 species, and (iii) the *X*. *heloidea* group contains 16 species. One of the newly described or unnamed species belongs to the *X*. *filiformis* group—*X*. *vermiformis*; nine of them belong to the *X*. *phyllocharis* group—*X. allima, X. appendiculatoides, Xylaria minuscula*, *X*. *neblinensis*, *X*. sp. AR1741, *X*. sp. GS7461B, *X. spiculaticlavata*, *X*. *vittatipiliformis*, and *X*. *vittiformis*; and three of them belong to the *X*. *heloidea* group—*X*. *hispidipes*, *X*. *imminuta*, and *X*. sp. GS7461A.

**Conclusion:**

The 45 species of *Xylaria* associated with fallen leaves and petioles can be identified by using the dichotomous identification key that we provided herein. It is important to note that most of the studied species are represented by only one or several specimens and many have not been recollected and cultured.

## Background

Fallen leaves and petioles represent one of the four general substrate types, with which *Xylaria* species are associated. Most *Xylaria* species produce stromata on dead wood and branches, while fewer species are associated with fallen fruits and seeds (Ju et al. 2018), termite nests and soil (Hsieh et al. 2022; Ju and Hsieh 2007; Ju et al. 2022; Rogers et al. 2005; Wangsawat et al. 2021), and fallen leaves and petioles. One issue in identifying *Xylaria* species inhabiting fallen leaves and petioles stemmed from the name *X*. *filiformis* (Albertini & Schwein.) Fr., which has been commonly applied to *Xylaria* collections from fallen leaves, petioles or even other substrates worldwide. One particular case is the confusion between *X*. *filiformis*, an uncommonly collected species, and *X*. *simplicissima* (Pers.) Y.-M. Ju & H.-M. Hsieh, a more commonly encountered species but often immature, because of their overlapping distributions in Europe and likely in North America. Specimens containing *X. simplicissima* were labeled as *X*. *filiformis* in most cases. Similar confusion can occur with other species that have filiform or cylindrical stromata with evident perithecial mounds on the surface.

Recent molecular phylogenetic studies (Hsieh et al. 2010; U’Ren et al. 2016) have shown that leaf- and petiole-inhabiting *Xylaria* species are not necessarily closely related, even though they share the same growth substrate type. These species formed more than one clade among *Xylaria* species associated with other substrate types.

*Xylaria* species growing on fallen leaves and petioles have not been treated systematically, posing an obstacle in identifying these fungi. Rogers et al. (1987), while commenting on page 170 on several small leaf-inhabiting *Xylaria* species collected from rain forests of North Sulawesi, Indonesia, stated that these *Xylaria* species “are in urgent need of study on a worldwide basis.” *Xylaria* species that inhabit leaves and petioles are often overlooked because they produce delicate and often minute stromata compared to those growing on other substrate types. In many cases, their stromata are only produced scarcely, being represented by one or several stromata only. It is not uncommon that stromata of different species are growing together even on the same leaf; examples can be found in the isotype specimens of *X*. *phyllophila* Ces. (K ex Cooke herb.), where it is mixed with *X*. *minuscula* Y.-M. Ju & H.-M. Hsieh, and the isotype of *X*. *phyllocharis* Mont. (PC ex Leprieur herb.), where it is mixed with *X*. *aristata* Mont. var. *aristata* and *X*. *spiculaticlavata* Y.-M. Ju & H.-M. Hsieh.

In this study, we aimed to resolve the issues revolving around the name *X*. *filiformis*, particularly in answering what the species really is and those species that have been confused with it. We went through published *Xylaria* names from fallen leaves and petioles and managed to study their type and/or authentic materials. *Xylaria* species growing on fallen leaves and petioles are separated into three groups herein, i.e., the *X. filiformis* group, the *X*. *phyllocharis* group, and the *X*. *heloidea* Penz. & Sacc. group, mainly by stromatal shape, compactness of perithecial aggregation, and conspicuousness of perithecial mounds on the stromatal surface. It is important to note that these groups are not natural assemblages and that the grouping is by no means an attempt to reflect close relatedness within each group. Instead, the grouping is simply a convenient way to convey the general morphology of the taxa included within each group.

The *X*. *filiformis* group contains *X*. *filiformis* and nine other species—*X*. *castilloi* San Martín & J. D. Rogers, *X*. *diminuta* San Martín & J. D. Rogers, *X*. *duranii* San Martín & Vanoye, *X*. *eugeniae* San Martín, Vanoye & P. Lavín, *X*. *meliacearum* Læssøe, *X*. *noduliformis* Y.-M. Ju & H.-M. Hsieh, *X*. *simplicissima*, *X*. *vagans* Petch, and *X*. *vermiformis* Y.-M. Ju, H.-M. Hsieh, which are characterized by filiform stromata and half-exposed to fully exposed perithecial mounds. *Xylaria noduliformis*, although having half-exposed perithecial mounds, is like species of the *X*. *phyllocharis* group in possessing stouter stromata. The *X*. *phyllocharis* group contains 19 taxa—*X*. *allima* Y.-M. Ju & H.-M. Hsieh, *X*. *appendiculata* Ferd. & Winge, *X*. *appendiculatoides* Y.-M. Ju & H.-M. Hsieh, *X*. *asperata* J. D. Rogers, Callan, Rossman & Samuels, *X*. *foliicola* G. Huang & L. Guo, *X*. *kamatii* Pande, *X*. *lima* Höhn., *X*. *luxurians* (Rehm) C. G. Lloyd, *X*. *maitlandii* (Dennis) D. Hawksworth, *X*. *minuscula*, *X*. *neblinensis* Y.-M. Ju & H.-M. Hsieh, *X*. *phyllocharis*, *X*. *phyllophila*, *X*. *polysporicola* H.-X. Ma & X.-Y. Pan, *X*. sp. AR1741, *X*. sp. GS7461B, *X*. *spiculaticlavata*, *X*. *vittatipiliformis* Y.-M. Ju, H.-M. Hsieh & Fournier, and *X*. *vittiformis* Y.-M. Ju & H.-M. Hsieh. Their perithecial mounds are unexposed, being inconspicuous in most species but undulate in *X*. *appendiculatoides*, *X*. *luxurians*, *X*. *minuscula*, *X*. *neblinensis*, and *X*. *phyllophila*. The 16 taxa of the *X*. *heloidea* group—*X*. *amphithele* San Martín & J. D. Rogers, *X*. *aristata* var. *aristata*, *X*. *aristata* var. *hirsuta* Theiss., *X*. *axifera* Mont., *X*. *clusiae* K. F. Rodrigues, J. D. Rogers & Samuels, *X*. *delicatula* Starb., *X*. *heloidea*, *X*. *hispidipes* Y.-M. Ju & H.-M. Hsieh, *X*. *hypsipoda* Massee, *X*. *imminuta* Y.-M. Ju & H.-M. Hsieh, *X*. *memecyli* Pande, *X*. *nainitalensis* Dargan, *X*. *petchii* C. G. Lloyd, *X*. *pisoniae* D. Scott, J. D. Rogers & Y.-M. Ju, *X*. *sicula* Pass. & Beltr., and *X*. sp. GS7461A—are characterized by having a capitate or obconical fertile part of stromata. Their perithecia are clumped together in stromata, with *X*. *petchii* being peculiar in having perithecia clumped together near the top of a stroma but scattered below. The apex of stromata is fertile in certain taxa but sterile in others.

## Methods

Materials of leaf- and petiole-inhabiting *Xylaria* taxa were studied from various herbaria, including BO, BPI, C, HAST, HBG, K, L, NY, PC, PH, S, UPS, and WSP. Herbarium abbreviations are in accordance with Index Herbariorum (http://sweetgum.nybg.org/science/ih/). Cultures were obtained by placing tissue or ascospores from freshly collected stromata on SME medium (Kenerley and Rogers 1976). Resulting colonies were transferred to 9-cm plastic Petri dishes containing 2% Difco oatmeal agar (OA), from which the culture descriptions were made, and were incubated at 20 °C under 12 h fluorescent light. Asci and ascospores were mounted in water and Melzer’s reagent and examined using differential interference microscopy and bright-field microscopy. Images of stromata, perithecia, asci and ascospores were taken and measured with the software Piximetre 5.10, which is available at http://ach.log.free.fr/Piximetre/.

Methods for obtaining sequences of nuc rDNA internal transcribed spacers (ITS1-5.8 S-ITS2 = ITS) followed Hsieh et al. (2009).

## Taxonomy

### Accepted taxa

**Fig. 1:**
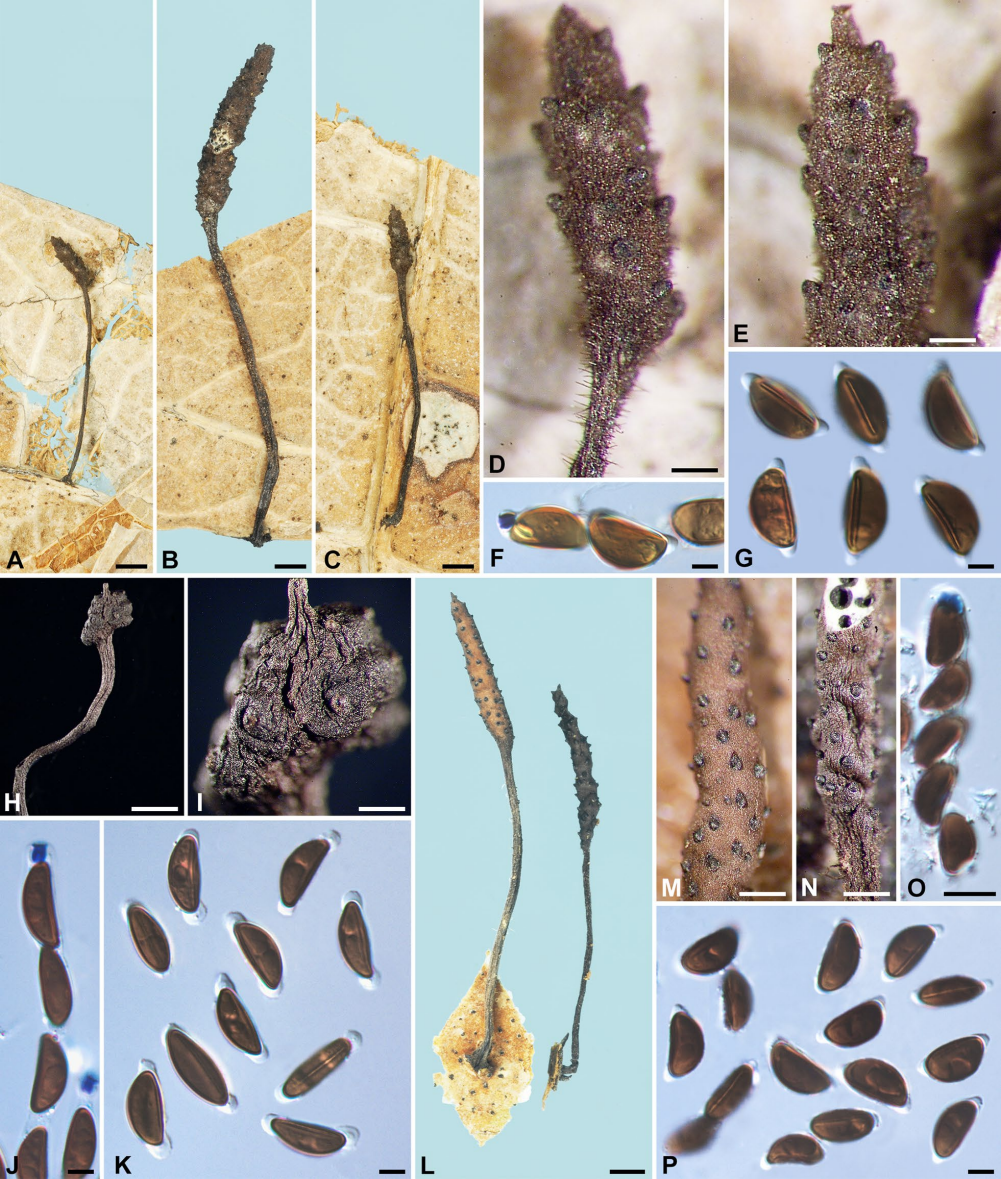
*Xylaria allima*, *X*. *amphithele*, and *X*. *appendiculata*. **A**–**G** *X*. *allima* (holotype). **A**–**C** Stromata. **D**, **E** Stromatal surfaces coated with a tomentum and showing coarsely conic-papillate ostioles that are tilting upwards; **D** also showing stiff hairs on the stipe. **F** Ascal apical ring bluing in Melzer’s reagent and ascospores. **G** Ascospores. **H**–**K** *X*. *amphithele* (HAST 145965). **H** Stroma. **I** Stromatal surface. **J** Ascal apical rings and ascospores. **K** Ascospores. **L**–**P** *X*. *appendiculata* (HAST 145966). **L** Stromata. **M**, **N** Stromatal surfaces, immature in **M** and fully mature in **N **. **O** Ascal apical ring and ascospores. **P** Ascospores. Bars in **A**–**C**, **L** = 1 mm; **D**, **E** = 0.25 mm; **H** = 2 mm; **I**, **M**, **N **= 0.5 mm; **F**, **G**, **J**, **K**, **O**, **P** = 5 μm

***Xylaria allima*** Y.-M. Ju & H.-M. Hsieh, sp. nov. Fig. 1A–G.

MycoBank MB848538.

**Typification** PANAMA. Canal Zone, Fort Sherman area, on fallen leaves, 5 Aug 1945, *Martin, G. W. 6154*, as *X. appendiculata* Ferd. & Winge by Dennis, R. W. G. (holotype of *X. allima* K[M] 236751).

**Etymology** Near *X. lima* in having a tomentose stromatal surface and a non-cellular appendage on each end of the ascospores.

Stromata cylindrical at fertile part, unbranched, with an acute apex, on a long, hirsute stipe, 8.6–16.5 mm in total length, 2–6 mm long × 0.6–1.1 mm broad at fertile part; surface dark vinaceous brown, with slight perithecial mounds, overlain entirely with brown tomentum, underlain with a thin, soft layer ca. 10 μm thick or less, concolorous with the surface; interior white, homogeneous, soft. Perithecia spherical, 250–350 μm broad. Ostioles coarsely conic-papillate, tilting upwards, 100–120 μm high × 110–130 μm broad at base. Asci not intact, with an apical ring staining blue in Melzer’s iodine reagent, urn-shaped, 3.5–4 μm high × 3.5–4 μm broad. Ascospores brown to dark brown, unicellular, ellipsoid-inequilateral, with narrowly rounded to broadly rounded ends, smooth, (14.5–)15–16.5(–17) × (8–)8.5–9.5(–10) µm (15.7 ± 0.7 × 9.0 ± 0.4 μm, N = 40), with a straight germ slit slightly less than spore-length on the ventral side, surrounded with a hyaline sheath swelling at both ends to form papillate non-cellular appendages; epispore smooth.

**Notes** *Xylaria allima* features a dark vinaceous brown, tomentose stromatal surface, beneath which a black layer is lacking, and conic-papillate ostioles tilting upwards. These features are also shared by *X*. *lima*, which can be readily separated from *X*. *allima* by smaller ascospores (10–)10.5–12(–14) × (5–)6–7(–7.5) µm.

*Xylaria appendiculata* sensu Dennis (1956) is based on the collection *Martin 6154* (K[M] 236751), which, however, differs from *X*. *appendiculata* that we define herein by the tomentose stromatal surface and larger ascospores. The ascospore size range given by Dennis (1956) is 12–15 × 7–8 μm, somewhat smaller than our measurement. It should be noted that a duplicate of the collection *Martin 6154* in BPI is not the same as the current species but is instead *X. lima*, which has smaller ascospores.

***Xylaria amphithele*** San Martín & J. D. Rogers, Mycotaxon 34: 304. 1989. Figs. 1H–K, 12H.

Stromata conical to globose at fertile part, unbranched, with or without a sterile apex, on a long, glabrous stipe, up to 50 mm in total length, 1.5–2.5 mm long × 1–2.5 mm broad at fertile part; surface blackish, with conspicuous to half-exposed perithecial mounds, lacking an outer layer, underlain with a thin, soft layer ca. 10 μm thick or less, concolorous with the surface; interior white, homogeneous, soft. Perithecia spherical, 300–550 μm broad. Ostioles slightly papillate, ca. 50 μm broad at base. Asci with eight ascospores arranged in uniseriate manner, cylindrical, 130–220 μm total length, the spore-bearing part 80–120 μm long × 7–9 μm broad, with an apical ring staining blue in Melzer’s iodine reagent, inverted hat-shaped, 3.5–4.5 μm high × 2–3 μm broad. Ascospores brown to dark brown, unicellular, ellipsoid-inequilateral, with narrowly rounded ends, smooth, (12–)12.5–15.5(–17) × (5–)6–7.5(–8) µm (14.0 ± 1.5 × 6.7 ± 0.8 μm, N = 60), with a straight germ slit nearly spore-length to spore-length on the ventral side, surrounded with a hyaline sheath swelling at both ends to form papillate non-cellular appendages, sometimes retaining a cellular appendage within a non-cellular appendage; epispore smooth.

Cultures and anamorph. Colonies reaching the edge of 9-cm Petri dish at 1.5 wk, whitish to pinkish, becoming blackish from center outwards, appressed, azonate, with diffuse margins. Reverse uncolored. Stromata arising copiously from the entire colony surface, cylindrical, tapering upwards, unbranched, up to 2.5 cm long × ca. 0.6 mm diam, becoming black from base upwards, white on the surface of upper part. Anamorph not produced.

**Specimens examined** FRENCH WEST INDIES. Guadeloupe, ravine Blondeau, on dead leaves, 4 Sep 2005, *Lechat, C. L. 5352* (cultured) (HAST 145965), GenBank: ITS = GU300083. MEXICO. Tamaulipas state, Gómez Farias, on dried fallen leaves in median subdeciduous rain forest, Jul 1987, *San Martín, F. 207* (isotype of *X. amphithele* WSP ex Rogers herb.).

**Notes** *Xylaria amphithele* is known thus far from Mexico and Guadeloupe, both located in Central America. It is characterized primarily by having the fertile part of stromata conical to globose and the ascospore enclosed within a hyaline sheath conspicuously swelling at both ends. *Xylaria amphithele* clustered with two other foliicolous species, *X*. *vagans* and *X*. *aristata*, in the phylogenetic study of Hsieh et al. (2010), where these two species were labeled as *X*. sp. 6 and *X*. *sicula* f. *major*, respectively.

**Fig. 2:**
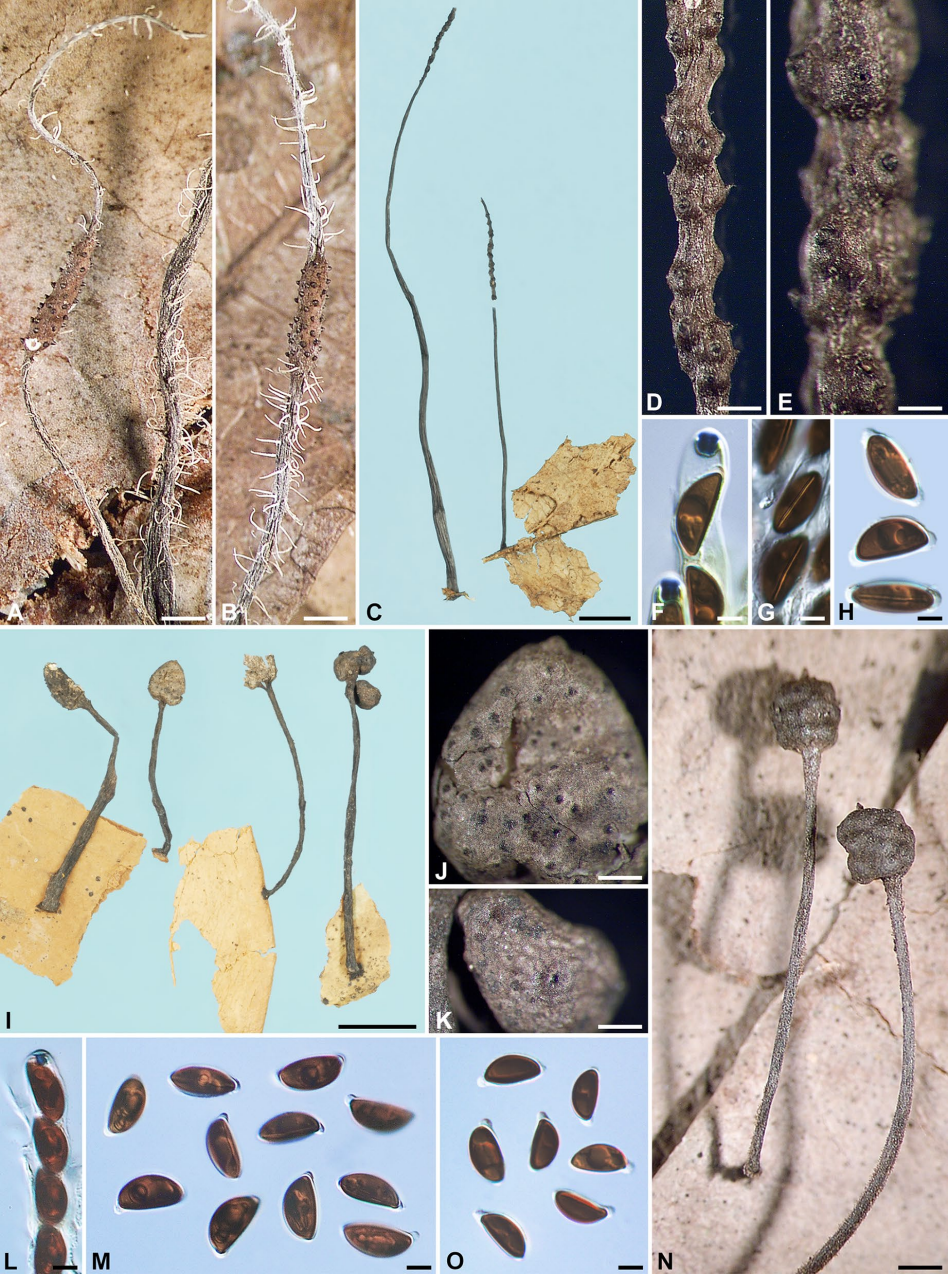
*Xylaria appendiculata*, *X*. *appendiculatoides*, and *X*. *aristata* var. *aristata*. **A**, **B** *X*. *appendiculata* (BPI 881476 GS4843 from Guyana), immature stromata showing perpendicular pegs arising from the surface. **C**–**H** *X*. *appendiculatoides* (holotype). **C** Stromata. **D**, **E** Stromatal surfaces showing conic-papillate ostioles that are tilting upwards. **F** Ascal apical rings and ascospores. **G**, **H** Ascospores. **I**–**O** *X*. *aristata* var. *aristata* (**I**–**M** holotype; **N**, **O** WSP San Martín 1121 from Mexico). **I**, **N** Stromata. **J**, **K** Stromatal surface. **L** Ascal apical ring and ascospores. **M**, **O** Ascospores. Bars in **A**, **B**, **N** = 1 mm; **C**, **I** = 5 mm; **D**, **J**, **K** = 0.5 mm; **E** = 0.25 mm; **F**–**H**, **L**, **M**, **O** = 5 μm

***Xylaria appendiculata*** Ferd. & Winge, Bot. Tidsskr. 29: 17. 1908. Figs. 1L–P, 2A, B.

≡ *Xylosphaera appendiculata* (Ferd. & Winge) Dennis, Kew Bull. 13: 102. 1958.

≡ *Podosordaria appendiculata* (Perd. & Winge) P. M. D. Martin, J. S. African Bot. 36: 131. 1970, nom. inval. ICN Art. 41.1 (Shenzhen Code); J. S. African Bot. 42: 79. 1976.

? = *Xylaria phyllocharis* Mont. var. *hirtella* Theiss., Ann. Mycol. 6: 343. 1908.

Stromata short-cylindrical to long-cylindrical at fertile part, unbranched, with a blunt or acuminate apex, on a hirsute stipe or subsessile to astipitate, (1 –)8–20(–28) mm in total length, 1–13 mm long × 0.7–1.5 mm broad at fertile part; surface brown to dark vinaceous brown except for black perithecial mounds, lacking perithecial mounds when young, becoming slightly undulate, finely longitudinally striated at maturity, lacking an outer layer, underlain with a thin, soft layer ca. 10 μm thick or less, concolorous with the surface; interior white, homogeneous, soft. Perithecia spherical, 200–250 μm broad. Ostioles coarsely conic-papillate, tilting upwards, 110–130 μm high × 100–130 μm broad at base. Asci with eight ascospores arranged in uniseriate manner, cylindrical, 100–145 μm total length, the spore-bearing part 80–95 μm long × 9–13 μm broad, with an apical ring staining blue in Melzer’s iodine reagent, urn-shaped, 3–4 μm high × 3–3.5 μm broad. Ascospores dark brown, unicellular, ellipsoid-inequilateral, with narrowly rounded ends, smooth, (11.5–)12.5–14(–15) × (6–)6.5–7.5(–9) µm (13.1 ± 0.8 × 7.2 ± 0.5 μm, N = 60), with a straight germ slit nearly spore-length to spore-length on the ventral side, surrounded with a hyaline sheath swelling at both ends to form papillate non-cellular appendages; epispore smooth.

**Specimens examined** FRENCH GUIANA. ca. 10 km SW of Saul, track from Saul leading SW toward Mt. Galbao, elev. ca. 200 m, on dead leaves, 7–9 Jan 1986, *Samuels, G. J. & Boise, J. GS2643*, as *X. appendiculata* (HAST 145966 ex NY); ca. 20 km SW of Saul, Mt. Galbao, Upland moist forest with relatively few epiphytes and mosses on trees, alt. 650 m, on dead leaves, 22 Jan 1986, *Samuels, G. J. & Boise, J. GS3164*, as *X. appendiculata* (HAST 145967 ex NY). FRENCH WEST INDIES. Guadeloupe, Basse Terre, on fallen leaves of *Clusia venosa*, Nov 1992, *Vivant, J. GUAD60*, as *X. appendiculata* (WSP 74830). GUYANA. Cuyuni-Mazaruni Region, Mazaruni Subregion, Foothills immediately S of Mt. Ayanganna, alt. 550–650 m, on leaves, 26 Feb 1987, *Samuels, G. J. et al. GS4843*, as *X. phyllocharis* var. *hirtella* (BPI 881476 ex NY), immature; Cuyuni-Mazaruni Region, VII. Mazaruni Subregion, VII-2, Headwaters of Kangu R, W branch ca. 4 km NW of eastern peak of Mt. Ayanganna, first talus slope on sandstone, alt. 700 m, on dead leaves, 5–7 Mar 1987, *Samuels, G. J., Pipoly, J., Gharbarran, G. & Chin, J. GS5034*, as *X. appendiculata* (HAST 145968); Cuyuni-Mazeruni, Region VII, Mazeruni Subregion VII-2 and Potaro-Siparuni Region No. VIII, Potaro Subregion VIII, eastern side of Mt. Ayanganna, on decaying dicot leaves, 11–12 Mar 1987, *Samuels, G. J., Pipoly, J., Gharbarran, G. & Chin, J. GS5123*, as *X. appendiculata* (BPI 883911 ex NY). US VIRGIN ISLANDS. St. Croix, Caledonia Valley, on leaves of *Crescentia*, 2 Feb 1906, *Raunkiaer, C.*, *Plantae ex Ind. Occid. 1780* (holotype of *X. appendiculata* C). VENEZUELA. Edo. Merida, above Lagunillas, La Trampa, Loma El Burro, in remnant forest, alt. 2100 m. 8°33′0″N 71°27′0″W, on decaying *Clusia* leaves, 12 Nov 1990, *Samuels, G. J., Hein, B., Huhndorf, S. M., Holmquist, O., Iturriaga, T. & Hererra, M. GS7018*, as *X. appendiculata* (BPI 882429); Edo. Miranda, Parque Nacional Guatopo, trail between Agua Blanca and La Cruceta, alt. 500–600 m, on dicot leaves, 27–30 Nov 1990, *Samuels, G. J., Hein, B. & Huhndorf, S. M. GS7636*, as *X. appendiculata* (BPI 88201).

**Notes** *Xylaria appendiculata* was named and illustrated with emphasis on the conspicuous non-cellular appendages on the ascospore ends (Ferdinandsen and Winge 1908). Unlike most examined specimens where stromata are cylindrical and stipitate, the holotype specimen of *X*. *appendiculata* has stunted, sessile to short-stipitate stromata 1–2 mm high × 0.7–1 mm broad. The current species belongs to the *X*. *phyllocharis* group and differs from other species in the group with a similar ascospore size range mainly by having a glabrous stromatal surface, lacking an outer stromatal layer, and possessing non-cellular ascospore appendages.

*Xylaria appendiculata* was likely included within *X*. *phyllocharis* in Fournier et al. (2020), who noted a wide range of variations in ascospore morphology. *Xylaria appendiculata* differs from *X*. *phyllocharis* by having more conspicuous ostioles, larger ascospores, and a prominent hyaline sheath swelling at ascospore ends.

BPI 881476 is immature and has whitish perpendicular pegs arising from the young stromatal surface (Fig. 2A, B). These pegs, presumably synnematal remnants, seem to largely wither off during the maturation process and leave little trace of them on mature stromata. The original material of *X. phyllocharis* var. *hirtella* Theiss. appears to be missing. Theissen (1908) described the stromata being short-apiculate on top and having stipes covered with dark hairs. The stromatal surface had half-liberated perithecial mounds with hemispherical to conical ostioles. The ascospores were surrounded by a delicate hyaline sheath that hardly becomes appendiculate on the ends. The ascospore size range was not originally given but was later reported as 12–14 × 6.5–8.5 μm (Theissen 1909). Theissen’s descriptions of *X. phyllocharis* var. *hirtella* (Theissen 1908, 1909) seem to suggest the current species except for the hyaline sheath not being conspicuously detached at the ascospore ends.

The interpretation of *X*. *appendiculata* in (Dennis 1956) based on *Martin, G. W. 6154* from Panama (K[M] 236751) is *X*. *allima*, which has a tomentose stromatal surface.

***Xylaria appendiculatoides*** Y.-M. Ju & H.-M. Hsieh, sp. nov. Fig.2C–H.

MycoBank MB848539.

**Typification** BRAZIL. on fallen leaves, *Rick, J. 42*, as *X. filiformis* (holotype of *X. appendiculatoides* BPI 713892 ex Lloyd herb. 11867).

**Etymology** Referring to its similarity to *X. appendiculata*, from which it differs in having larger ascospores and protuberant perithecial mounds.

Stromata cylindrical at fertile part, unbranched, with the apex broken off, probably acute, on a glabrous stipe, 34–59 mm in total length, 8–11 mm long × 0.7–1 mm broad at fertile part, up to 35 mm long at stipe; surface blackish brown to black, slightly polished, with conspicuous perithecial mounds and fine longitudinal striations, lacking an outer layer, underlain with a thin, soft layer ca. 10 μm thick or less, concolorous with the surface; interior white, homogeneous, soft. Perithecia spherical, 250–350 μm broad. Ostioles conic-papillate, tilting upwards, 110–130 μm high × 120–150 μm broad at base. Asci not intact, with an apical ring staining deep blue in Melzer’s iodine reagent, urn-shaped, 4–4.5 μm high × 3.5–4 μm broad. Ascospores dark brown, unicellular, ellipsoid-inequilateral, with acute to narrowly to broadly rounded ends, smooth, (14–)15–16(–17) × (6.5–)7.0–7.5(–8) µm (15.3 ± 0.9 × 7.3 ± 0.3 μm, N = 40), with a straight germ slit spore-length on the ventral side, surrounded with a hyaline sheath swelling at both ends to form papillate non-cellular appendages; epispore smooth.

**Additional specimen examined** BRAZIL. on fallen leaves, *Rick, J. 21*, as *X.* cf. *filiformis* (BPI 713890 ex Lloyd herb. 11868).

**Notes** *Xylaria appendiculatoides* can easily be confused with *X. appendiculata* due to their similar stromatal morphology, coarsely conic-papillate ostioles, and prominent secondary appendages on the ascospores. However, they can be separated by the former species having protuberant perithecial mounds and longer ascospores (13.9–)14.4–16.2(–17.1) µm compared to (11.4–)12.3–13.9(–15.1) µm for *X*. *appendiculata*. The current species is known only from two specimens identified as *X*. *filiformis* in the Lloyd herbarium (BPI).

***Xylaria aristata*** Mont. **var.** ***aristata***, Ann. Sci. Nat., Bot., sér. IV, 3: 106. 1855. Figs. 2I–O and 12I.

≡ *Xylosphaera aristata* (Mont.) Dennis, Kew Bull. 13: 102. 1958.

≡ *Podosordaria aristata* (Mont.) P. M. D. Martin, J. S. African Bot. 36: 91. 1970, nom. inval. ICN Art. 41.1 (Shenzhen Code); J. S. African Bot. 42: 79. 1976.

= *Xylaria oocephala* Penz. & Sacc., Malpighia 11: 500. 1897.

? = *Xylaria setocephala* Yates, Philipp. J. Sci. 12: 379. 1917.

? = *Xylaria bogoriensis* C. G. Lloyd, Mycol. Writings 7: 1309. 1924.

= *Xylaria hainanensis* Y. F. Zhu & L. Guo, Mycosystema 30: 527. 2011.

= *Xylaria sphaerica* G. Huang & L. Guo, in G. Huang, R. S. Wang, L. Guo & N. Liu, Mycotaxon 130: 300. 2015.

Stromata capitate at fertile part, unbranched, with a mucronate to acicular apex, on a long, glabrous or hirsute stipe, 12–35 mm in total length, 1.5–2.5 mm broad at fertile part; surface grayish brown, dark brown to blackish, lacking perithecial mounds or with slight perithecial mounds, overlain with a thin grayish pellicle cracked reticulately into plaques 100–200 μm broad but gradually worn off when overmature, underlain with a carbonized layer ca. 50 μm thick; interior white, homogeneous, fragile. Perithecia spherical, 300–400 μm broad. Ostioles slightly papillate, ca. 100 μm broad at base. Asci with eight ascospores arranged in uniseriate to partially biseriate manner, cylindrical, 115–165 μm total length, the spore-bearing part 65–80 μm long × 6–10 μm broad, with an apical ring staining blue in Melzer’s iodine reagent, urn-shaped, 2.5–3.5 μm high × 2.5–3 μm broad. Ascospores brown to dark brown, unicellular, ellipsoid-inequilateral, with narrowly rounded ends frequently bearing a cellular appendage on one end, smooth, (10–)10.5–12.5(–14) × (5.5–)6–7(–7.5) µm (11.5 ± 1.1 × 6.5 ± 0.5 μm, N = 80), with a straight germ slit nearly spore-length to spore-length on the ventral side, lacking a hyaline sheath; epispore smooth.

Cultures and anamorph. Colonies reaching the edge of 9-cm Petri dish in 4 wk, whitish, immediately becoming blackish in patches from center outwards, appressed, azonate, with diffuse margins. Reverse pale pink. Stromata arising copiously from the entire colony surface, cylindrical, tapering upwards, unbranched, up to 2.5 cm long × 0.3–0.5 mm diam, becoming black from base upwards, white on the surface of upper part. Anamorph not produced.

**Specimens examined** BRAZIL. Pyrenopolis, on dead leaves, 1912, *Brockes, F. A.*, as *X. oocephala* (S F43698 ex Sydow herb.). FRENCH GUIANA. on fallen leaves, *Leprieur, F. R. 1209* (holotype of *X. aristata* var. *aristata* [in 2 packets] PC 0096736 & 0096737 ex Montagne herb. 10093 & 10094, respectively); Cayenne City, on dead dicot leaves, Mar 1986, *Samuels, G. J. GS4508*, as *X.* cf. *aristata* (BPI 881467 ex NY). GUYANA. Kopinang Village, Mt. Wokomung, alt. 1540–1570 m, on dead leaves, 12 Jul 1989, *Samuels, G. J., Boom, B. M. & Bacchus, G. GS6692*, as *X*. *aristata* (HAST 145969). INDIA. Kerala State, Kannur District, Andaloorkavu, on petiole of a fallen leaf, 5 Aug 2011, *Deepna Latha, K. P. DKP20*, as *X*. *aristata* (HAST 145970). INDONESIA. Java, Hortus Bogoriensis, in ramulis dejectis, 28 Dec 1896, *Penzig, O.* (holotype of *X. oocephala* BO 3310, isotype PAD); Java, Hortus Bogoriensis, on dead leaves, Jan 1950, *van Steenis, C. G. G. J.*, as *X. oocephala* (L ex Boedijn herb.); Java, Hortus Bogoriensis, on fallen leaves of *Palaquium* sp., Feb 1921, *van Overeem, C. 37a* (holotype of *X. bogoriensis* BPI 713657 ex Lloyd herb. 10316, isotype L ex U 120499), immature; Java, Hortus Bogoriensis, on petioles, 17 Nov 2017, *Ju, Y.-M. 1823* (cultured), as *X*. *aristata* (HAST 145971), GenBank: ITS = OQ883719; Java, Hortus Bogoriensis, on petioles, Apr 1923, *van Overeem, C.*, as *X. oocephala* (BO 521). MEXICO. Oaxaca State, Temazcal Town, in median subdeciduous tropical rain forest, on lamina and midribs of fallen leaves of “zapote chico”, 7 Oct 1988, *San Martín, F. 1051B*, as *X. delicatula* (WSP ex Rogers herb.); Oaxaca State, Temazcal Town, in median subdeciduous tropical rain forest, on petioles and lamina of fallen leaves, 8 Oct 1988, *San Martín, F. 1121*, as *X. delicatula* (WSP ex Rogers herb.); Quintana Roo State, Othón P. Blanco Municipality, Ejido La Unión, in median subdeciduous tropical rain forest, on lamina and veins of fallen leaves, 12 Nov 1988, *San Martín, F. 1271*, as *X. delicatula* (WSP ex Rogers herb.); Quintana Roo State, Othón P. Blanco Municipality, Ejido La Unión, in median subdeciduous tropical rain forest, on lamina and veins of fallen leaves, 15 Nov 1988, *San Martín, F. 1323*, as *X. delicatula* (WSP ex Rogers herb.); Quintana Roo State, Othón P. Blanco Municipality, Ejido La Unión, km 12 Cafetal-Mahahual Rd. in low subdeciduous tropical rain forest, on petioles and lamina of fallen leaves, 16 Nov 1988, *San Martín, F. 1300, 1367, 1427 & 1450*, as *X. delicatula* (WSP ex Rogers herb.). PHILIPPINES. Luzon, Province of Tayabas, Basiad, on fallen leaves of *Garcinia*, Dec 1916, *Yates, H. S. 25647* (holotype of *X. setocephala* BPI 584093, isotypes BPI 584094 ex MO, BPI 713652 ex Lloyd herb. 10092, K[M]), immature. TAIWAN. Ping-tung Co., Heng-chun, Ken-ting, on dead leaves, 13 Jul 2001, *Ju, Y.-M. & Hsieh, H.-M. 90071613*, as *X. oocephala* (HAST 145972), GenBank: ITS = GU300081.

**Notes** Stromata of *X. aristata* var. *aristata* are overlain with a grayish, reticulately cracked pellicle that gradually wears off at maturity. The stromatal apex is usually long and aciculate in its young stage and becomes short and apiculate when mature. *Xylaria delicatula* is similar in stromatal features but has darker, nearly semicircular to broadly ellipsoid-inequilateral ascospores. The cultures that we obtained produce copious stromata but lack an anamorph, much as reported by San Martín et al. (1997) (as *X*. *delicatula*).

The type materials of *X*. *setocephala* from the Philippines and *X*. *bogoriensis* from Indonesia are immature, but their stromatal features agree with those of *X*. *aristata* var. *aristata*. The protologues of *X*. *hainanensis* and *X*. *sphaerica* from China indicate that these two names are in synonymy with *X*. *aristata* var. *aristata*.

**Fig. 3:**
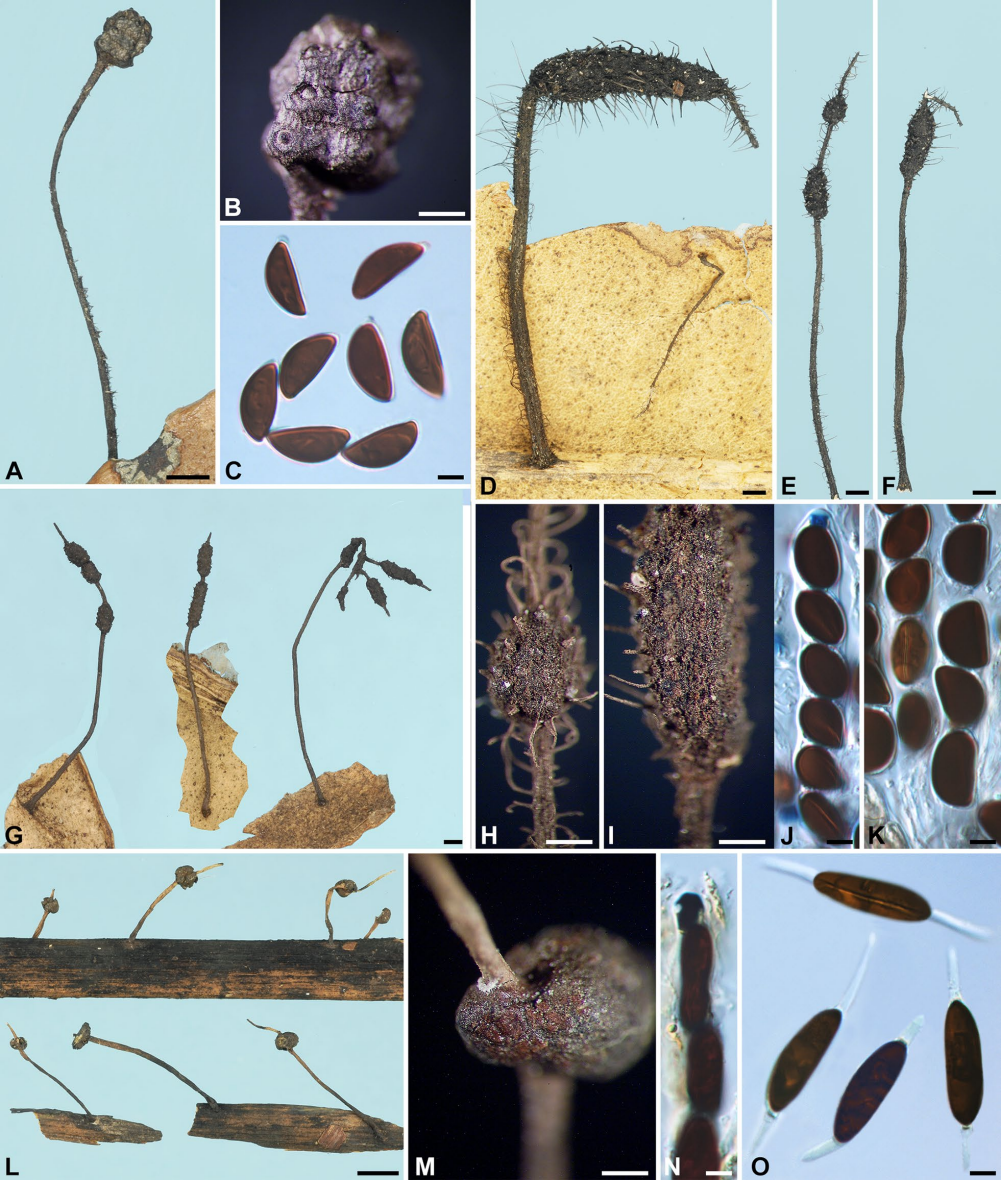
*Xylaria aristata* var. *hirsuta*, *X*. *asperata*, and *X*. *axifera*. **A**–**C** *X*. *aristata* var. *hirsuta* (lectotype). **A** Stroma. **B** Stromatal surface. **C** Ascospores. **D**–**K** *X*. *asperata* (isotype BPI 584098 except for **G**, which is from GS1410 [paratype NY]). **D**–**G** Stromata. **H**, **I** Stromatal surfaces overlain with a dark brown striped layer on fertile part and dark long spikes entirely. **J** Ascal apical ring and ascospores. **K** Ascospores. **L**–**O** *X*. *axifera* (holotype). **L** Stromata. **M** Stromatal surface. **N** Ascal apical ring and ascospores. **O** Ascospores. Bars in **A**, **D**–**G** = 1 mm; **B**, **H**, **I**, **M** = 0.5 mm; **L** = 5 mm; **C**, **J**, **K**, **N**, **O** = 5 μm

***Xylaria aristata*** Mont. **var.** ***hirsuta*** Theiss., Ann. Mycol. 6: 344. 1909. Fig. 3A–C.

**Typification** BRAZIL. on fallen leaves, *Theissen, F.* (lectotype [designated here, MycoBank Typification No. 10013065] of *X. aristata* var. *hirsuta* S F5664 ex Bresadola herb.).

Stromata capitate at fertile part, unbranched, with a rounded top, on a long, hirsute stipe, 10 mm in total length, 1.8 mm high × 1.6 mm broad at fertile part; surface grayish brown dark brown, with inconspicuous perithecial mounds, overlain with a thin grayish pellicle cracked reticulately into plaques 100–200 μm broad, underlain with a carbonized layer ca. 30 μm thick; interior white, homogeneous, fragile. Perithecia spherical, 400–500 μm broad. Ostioles coarsely conic-papillate, ca. 130 μm high × ca. 150 μm broad at base. Asci with eight ascospores arranged in uniseriate manner, cylindrical, 140–170 μm total length, the spore-bearing part 100–130 μm long × 7–9 μm broad, with an apical ring staining blue in Melzer’s iodine reagent, urn-shaped, 2.5–3 μm high × 2.5–3 μm broad. Ascospores dark brown, unicellular, ellipsoid-inequilateral, with narrowly rounded to acute ends frequently bearing a cellular appendage on one end, smooth, (13.5–)14–16(–17) × (5.5–)6–7(–7.5) μm (15.2 ± 1.0 × 6.3 ± 0.4 μm, N = 40), with a straight germ slit nearly spore-length to spore-length on the ventral side, lacking a hyaline sheath; epispore smooth.

**Notes** *Xylaria aristata* var. *hirsuta* is known only from its type specimen, which is highly similar to var. *aristata* except for its longer ascospores. Some collections of var. *aristata* have an ascospore length range approaching that of var. *hirsuta*. Further collections may bridge the gap between the two varieties.

***Xylaria asperata*** J. D. Rogers, Callan, Rossman & Samuels, in Rogers, Callan, Rossman & Samuels, Mycotaxon 31: 120. 1988. Fig. 3D–K.

Stromata ellipsoid, cylindrical to cylindric-fusoid at the continuous or discrete fertile parts, unbranched to occasionally branched once, with an acicular apex, on a long, hirsute stipe, 16–27 mm in total length, 1.2–3.8(–8.5) mm high × 0.8–1.4(–2.4) mm broad at fertile part; surface dark brown to blackish, with inconspicuous to conspicuous perithecial mounds, overlain with a dark brown striped layer on fertile part and dark long spikes entirely, underlain with a thin, black layer ca. 10 μm thick; interior white, homogeneous, soft. Perithecia spherical, 200–300 μm broad. Ostioles coarsely conic-papillate, ca. 150 μm broad at base. Asci with eight ascospores arranged in uniseriate manner, cylindrical, 120–160 μm total length, the spore-bearing part 80–95 μm long × 10–11 μm broad, with an apical ring staining blue in Melzer’s iodine reagent, cylindrical to urn-shaped, 2.5–3 μm high × 3–3.5 μm broad. Ascospores dark brown, unicellular, ellipsoid, strongly inequilateral, with narrowly rounded ends sometimes pinched on one end, smooth, (10.5–)11–12.5(–14.5) × (7–)7.5–8.5(–9) µm (11.9 ± 0.8 × 7.9 ± 0.5 μm, N = 40), with a straight germ slit spore-length or nearly so on the ventral side, lacking a hyaline sheath; epispore smooth.

Anamorph was reported by Rogers et al. (1988) as synnemata on the surface of the stromata collected from the field.

**Specimens examined** VENEZUELA. Cerro de la Neblina, along Rio Mawarinuma, just outside Canon Grande, vicinity of Neblina Base Camp, on fallen leaves of *Ficus* cf. *tonduzii*, Apr–May 1984, *Samuels, G. J. GS1410* (paratype of *X. asperata* NY); Territorio Federal Amazonas, Cerro de la Neblina, 5.1 km NE Pico Phelps, alt. 1730–1850 m, on dead leaves, 3 Feb 1985, *Rossman, A. Y. AR2224* (isotype of *X. asperata* BPI 584098); Territorio Federal Amazonas, Neblina Base Camp on Rio Mawarinuma, alt. 140 m, on thin leaves, 27 Jan 1985, *Rossman, A. Y. AR2394* (paratype of *X. asperata* BPI 584099).

**Notes** *Xylaria asperata* is unique in having dark spikes covering the entire stromatal surface. The dark long spikes appear to be the synnematal remnants persisting to the maturity. The outer stromatal layer is split into fine stripes, which are somewhat obscured by the spikes. The ascal apical rings are slightly broader than high, a feature not commonly found among *Xylaria* species.

***Xylaria axifera*** Mont., Ann. Sci. Nat., Bot., sér. IV, 3: 107. 1855. Fig. 3L–O.

≡ *Xylosphaera axifera* (Mont.) Dennis, Kew Bull. 13: 102. 1958.

≡ *Podosordaria axifera* (Mont.) P. M. D. Martin, J. S. African Bot. 36: 131. 1970, nom. inval. ICN Art. 41.1 (Shenzhen Code); J. S. African Bot. 42: 79. 1976.

Stromata capitate at fertile part, unbranched, with an acicular apex, on a long hirsute stipe, 6–25 mm in total length, 2–2.8 mm high × 2–3 mm broad at fertile part, up to 18 mm long × 0.5–1 mm at stipe; surface dark brown at the fertile part, yellowish brown at the sterile apex and stipe, with inconspicuous to conspicuous perithecial mounds, overlain with a dull reddish brown layer cracked reticulately into plaques 100–300 μm broad, underlain with a carbonized layer 50–80 μm thick; interior white, homogeneous, fragile. Perithecia spherical, 500–800 μm broad. Ostioles slightly papillate, ca. 100 μm broad at base. Asci with eight ascospores arranged in uniseriate to partially biseriate manner, cylindrical, 250–300 μm total length, the spore-bearing part 175–195 μm long × 7–10 μm broad, with an apical ring staining deep blue in Melzer’s iodine reagent, coffin-shaped to urn-shaped with an anchor-like upper rim, 7.5–9 μm high × 4.5–5.5 μm broad. Ascospores brown to dark brown, unicellular, ellipsoid-inequilateral, with broadly rounded ends, sometimes with one or both ends slightly pinched, smooth, (21.5–)22.5–24.5(–26) × (6.5–)7–8(–9) µm (23.6 ± 1.0 × 7.4 ± 0.6 μm, N = 40), with a straight to slightly oblique germ slit spore-length on the dorsal side, surrounded with a hyaline sheath swelling at both ends to form long tubular non-cellular appendages up to 40 μm; epispore smooth.

Cultures and anamorph were reported by Læssøe and Lodge (1994). The anamorph was not produced in culture but on the stromata collected from the field.

**Specimens examined** CUBA. on petioles, *Wright, C.* (K[M]); on petioles, *Wright, C.*, *Fungi Cubenses Wrightiani 798* (6 parts), as *X*. *axifera* (K[M]). DOMINICAN REPUBLIC. Jarabacoa, coffee plantation #24, 650 elev., on petioles of *Schefflera*, 27 Mar 1993, *Lodge, D. J. 2*, as *X*. *axifera* (K[M] 25655). GUYANA. on petioles, *Leprieur, F. R. 1192* (holotype of *X. axifera* PC 0086061 ex Montagne herb. 10095, isotypes K[M] 130076 ex Berkeley herb., K[M] ex Currey herb., PC ex Leprieur herb.). PERU. Dep. Huanuco, Tingo María, grounds of Universidad Agraria, on petiole of Araliaceae, 6 Jul 1987, *Læssøe, T. TL-P-197*, as *X*. *axifera* (K[M] 20529). PUERTO RICO. Bosque Nacional del Caribe, Bisley, on fallen petioles of *Schefflera*, 3 Dec 1997, *Legon, N. W. PR317*, as *X*. *axifera* (K[M] 57023); Luquillo Exp. Forest, El Verde Research Area, on petioles of *Schefflera morototoni*, 15 May 1988, *Lodge, D. J. PR605*, as *X*. *axifera* (K[M] 25564); Luquillo Exp. Forest, El Verde Research Area, on petioles of *Schefflera morototoni*, 21 Jan 1991, *Lodge, D. J. PR614*, as *X*. *axifera* (K[M] 25563); Luquillo Exp. Forest, El Verde Research Area, on petioles of *Schefflera morototoni*, Jan 1991, *Lodge, D. J. PR682*, as *X*. *axifera* (K[M] 25576); Luquillo Exp. Forest, El Verde Research Area, on petioles of *Schefflera morototoni*, 14 Sep 1992, *Lodge, D. J. PR860*, as *X*. *axifera* (K[M] 25575); Luquillo Exp. Forest, El Verde Research Area, on petioles of *Schefflera morototoni*, 12 Dec 1992, *Lodge, D. J. PR957*, as *X*. *axifera* (K[M] 25561); Sierra de Cayey, Guavate, on petioles of *Schefflera morototoni*, 8 Dec 1990, *Lodge, D. J. et al. PR613*, as *X*. *axifera* (K[M] 25565); Sierra de Cayey, nr. Jájome Alto, on petioles of *Schefflera morototoni*, 18 Jun 1988, *Lodge, D. J. PR607*, as *X*. *axifera* (K[M] 25562). TRINIDAD AND TOBAGO. Port of Spain, Emperor Valley, on petioles of *Panax*, *Thaxter, R. 8709*, as *X*. *axifera* (K[M] ex NY).

**Notes** *Xylaria axifera* differs from other leaf- and petiole-inhabiting species by having a reddish brown outer stromatal layer cracked into fine scales and ascospores longer than 20 μm, possessing a straight to slightly oblique germ slit on the dorsal side and a long tubular non-cellular appendages at each end, and a preference for petioles. Læssøe and Lodge (1994) considered *X*. *axifera* to be host-specific to Araliaceae.

*Xylaria axifera* Mont. var. *perexigua* Penz. & Sacc. [INDONESIA. Java, Hortus Bogoriensis, in fragmentis ligneis et foliis coriaceis dejectis, 21 Dec 1896, *Penzig, O.* (holotype BO 3317)] has prominent perithecial mounds on the stromatal surface but is immature as already documented in Penzig and Saccardo (1897). The stromata in the holotype packet bear no resemblance to those of *X*. *axifera*. In general, they are like those of *X*. *sicula*, *X*. *pisoniae*, and their likes.

***Xylaria castilloi*** San Martín & J. D. Rogers, in San Martín, Rogers & Lavín, Revta Mex. Micol. 13: 60. 1997.

For descriptions of the teleomorph, see San Martín et al. (1997) where illustrations of stromata, ascus, and ascospores are also provided. *Xylaria castilloi* is characterized by the following features: stromata cylindrical at fertile part, unbranched, with an obtuse sterile apex, on a hirsute stipe, 15–20 mm in total length × 2–3 mm broad at fertile part, with a blackish surface where perithecial mounds are half-exposed to fully exposed, overlain with stiff hairs and remnants of outer stromatal layer, with a white, fairly hard interior; perithecia spherical, 400–500 μm broad, with an inconspicuous to slightly papillate ostiole; ascospores brown, ellipsoid-inequilateral to crescentic, with narrowly rounded ends, (11.5–)12–14(–15) × (4.5–)5 μm, with a straight germ slit spore-length or nearly so on the ventral side, lacking a hyaline sheath.

Cultures and anamorph were reported by San Martín et al. (1997) with obovoid to ellipsoid conidia (3.5–)4–4.5(–5) × 2–2.5 μm.

**Notes** *Xylaria castilloi* is known only from its type material collected from Mexico (San Martín et al. 1997). Among the species with the stromatal surface overlain with hairs or spikes, *X*. *castilloi* can easily be separated from *X*. *allima*, *X*. *asperata*, *X*. *lima*, and *X*. *maitlandii* mainly by its half-exposed to fully exposed perithecial mounds.

***Xylaria clusiae*** K. F. Rodrigues, J. D. Rogers & Samuels in Samuels & Rogerson, Mem. N. Y. Bot. Gard. 64: 176. 1990.

= *Xylaria lindericola* H.-X. Ma & X.-Y. Pan, in X.-Y. Pan, Z.-K. Song, Z. Qu, T.-D. Liu & H.-X. Ma, MycoKeys 86: 55. 2022.

See Samuels and Rogerson (1990) for a description and Ju et al. (2018) for notes and illustrations. The ascospores are dark brown to blackish brown, ellipsoid-inequilateral, (12.5–)13–15(–16) × (7.5–)8–9(–10) µm (13.9 ± 0.8 × 8.6 ± 0.5 μm, N = 40), with a spore-length germ slit, lacking a hyaline sheath. *Xylaria clusiae* grows on fallen leaves and occasionally fallen fruits of *Clusia* species (Samuels and Rogerson 1990). The protologue of *X*. *lindericola* indicates that this name is in synonymy with *X*. *clusiae*.

**Fig. 4:**
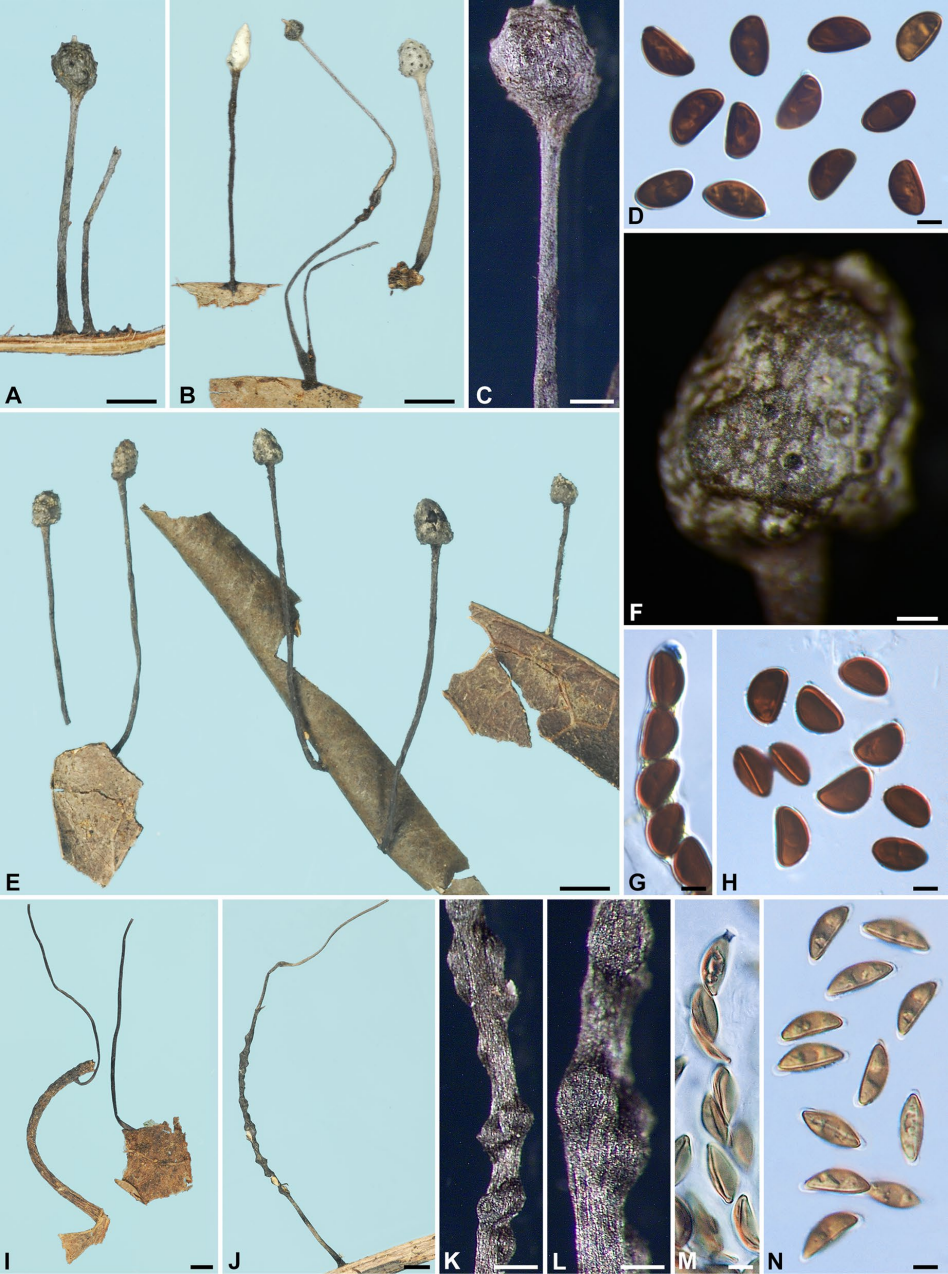
*Xylaria delicatula* and *X*. *filiformis*. **A**–**H** *X*. *delicatula* (**A**–**D** holotype; **E**–**H** GS2775 from French Guiana). **A**, **B**, **E** Stromata; mature stroma in **A** and various immature stages in **B**. **C**, **F** Stromatal surfaces. **D**, **H** Ascospores. **G** Ascal apical ring and ascospores. **I**–**N** *X*. *filiformis* (I isolectotype BPI 799791; J–N K[M] 169694 from France). **I**, **J** Stromata; the isolectotype in I is immature. **K**, **L** Stromatal surfaces. **M** Ascal apical ring and ascospores. **N** Ascospores. Bars in **A**, **B** = 1.25 mm; **C**, **K** = 0.5 mm; **E** = 2.5 mm; **F**, **L** = 0.25 mm; **I** = 5 mm; **J** = 1 mm; **D**, **G**, **H**, **M**, **N** = 5 μm

***Xylaria delicatula*** Starb., Bih. Kongl. Svenska Vetensk.-Akad. Handl. 27, 3: 18. 1901. Fig. 4A–H.

Stromata capitate at fertile part, unbranched, with a mucronate to acicular apex, on a long, glabrous stipe, 7–24 mm in total length, 1.1–2.2 mm long × 1.0–1.6 mm broad at fertile part; surface dark brown to blackish, lacking perithecial mounds or with slight perithecial mounds, overlain with a whitish pellicle when young, cracked reticulately into plaques 100–300 μm and gradually worn off at maturity, underlain with a carbonized layer ca. 50 μm thick; interior white, homogeneous, soft. Perithecia spherical, 300–400 μm broad. Ostioles papillate, ca. 100 μm broad at base. Asci with eight ascospores arranged in uniseriate manner, cylindrical, 140–170 μm total length, the spore-bearing part 70–80 μm long × 7–8 μm broad, with an apical ring staining blue in Melzer’s iodine reagent, urn-shaped, 3–4.5 μm high × 3–3.5 μm broad. Ascospores dark brown, unicellular, nearly semicircular to broadly ellipsoid-inequilateral, with broadly rounded ends to, less frequently, narrowly rounded ends sometimes bearing a cellular appendage on one end, smooth, (10–)10.5–12.5(–14) × (5.5–)6–7(–7.5) µm (11.3 ± 1.0 × 6.6 ± 0.5 μm, N = 70), with a straight germ slit spore-length or nearly so on the ventral side, lacking a hyaline sheath; epispore smooth.

**Specimens examined** BRAZIL. Matto Grosso, Cuyabá, on decayed leaves, 22 Mar 1894, *Malme, G. A. 577* (holotype of *X. delicatula* S F43701). FRENCH GUIANA. between SW of Saul, track between Saul and Mt. Galbao, alt. 450–500 m, on decaying leaves, 11 Jan 1986, *Samuels, G. J. & Boise, J. GS2775*, as *X*. *delicatula* (HAST 145973), GenBank: ITS = OQ883720; km 16 on road between Sinnamary and St. Elie, Orstom Research Area “Ecerex”, primary forest, on decaying dicot leaves, 20 Feb–1 Mar 1986, *Samuels, G. J. GS4039*, as *X. aristata* (BPI 881417 ex NY). GUYANA. Demerara-Mahaica Region, IV, Mahaica Subregion, IV-1, Linden Highway between Georgetown and Yarowcabra, Yarowcabra Forestry Station, elev. 50–100 m, on decaying leaf, 26, 27 Apr 1987, *Samuels, G. J. & Pipoly, J. GS5555*, as *X*. *delicatula* (BPI 883855); Mt. Wokomung, on decaying leaves, no date, *Samuels, G. J. & Pipoly, J. GS6369*, as *X. aristata* (NY).

**Notes** *Xylaria delicatula* resembles *X*. *aristata* var. *aristata* and var. *hirsuta* in stromatal morphology. It differs from these two taxa by frequently having darker ascospores and strongly inequilateral ascospores with broadly rounded ends.

***Xylaria diminuta*** San Martín & J. D. Rogers, in San Martín, Rogers & Lavín, Revta Mex. Micol. 13: 63. 1997.

For a description of the teleomorph, see San Martín et al. (1997) where illustrations of stromata, ascus, and ascospores are also provided. *Xylaria diminuta* is characterized by the following features: stromata cylindrical at fertile part, unbranched, with an acute apex, on a glabrous stipe, 5–13 mm total length × 0.5–1.5 mm broad, with a blackish surface where the perithecial mounds are half-exposed to fully exposed and frequently clumped together along the axis, with a white, hard interior; perithecia spherical, 200–300 μm broad, with a papillate ostiole; ascospores light brown to brown, ellipsoid-inequilateral, with rounded to narrowly rounded ends, (5.5–)6–7 × 3–3.5(–4) µm, lacking a hyaline sheath, with a straight germ slit nearly spore-length on the ventral side.

**Notes** *Xylaria diminuta* belongs to the *X*. *filiformis* group and is unique among the foliicolous *Xylaria* species in having the smallest ascospore size range.

***Xylaria duranii*** San Martín & Vanoye, in San Martín, Lavín & Rogers, Mycotaxon 79: 342. 2001.

? = *Xylaria betulicola* H.-X. Ma, Lar.N. Vassiljeva & Y. Li, in H.-X. Ma & Y. Li, Sydowia 70: 39. 2018.

For a description of the teleomorph, see San Martín et al. (2001) where illustrations of stromata, ascus, and ascospores are also provided. *Xylaria duranii* is characterized by the following features: stromata filiform at fertile part, unbranched, with an acute apex, on a villose stipe, 10–20 mm in total length × 1–1.5 mm broad, with a blackish surface where perithecial mounds are half-exposed to fully exposed, with a white, soft interior; perithecia spherical, 300–400 μm broad, with a slightly papillate to papillate-hemispherical ostiole; ascospores light brown to brown, ellipsoid-inequilateral to somewhat crescentic, with narrowly rounded ends sometimes pinched at one end, 12–14.5(–16) × 4–4.5(–5) µm, with a straight to slightly sigmoid germ slit spore-length on the ventral side, lacking a hyaline sheath.

**Notes** This Mexican species grows on leaves and fruit remains of *Quercus polymorpha* and is known only from its type material. It belongs to the *X*. *filiformis* group and can be separated from the species in this group with similar ascospore size ranges by having a glabrous stromatal fertile part and lacking a hyaline sheath on the ascospores. The protologue of *X. betulicola* in general suggests its resemblance to *X*. *duranii* except for the stromata terminating into a long acicular stromatal apex.

***Xylaria eugeniae*** San Martín, Vanoye & P. Lavín, in San Martín, Rogers & Lavín, Revta Mex. Micol. 13: 64. 1997.

For a description of the teleomorph, see San Martín et al. (1997) where illustrations of stromata, ascus, and ascospores are also provided. *Xylaria eugeniae* is characterized by the following features: stromata filiform at fertile part, unbranched, with an acute apex, on a glabrous stipe, 15–20 mm in total length × 1–1.2 mm broad, with a blackish surface where perithecial mounds are half-exposed to fully exposed, with a white, soft interior; perithecia spherical, 200–400 μm broad, with a papillate to hemispherical ostiole; ascospores brown to dark brown, ellipsoid-inequilateral, with narrowly rounded ends sometimes slightly pinched, 12–13.5 × 4–5 μm with a straight germ slit spore-length on the ventral side, surrounded with a hyaline sheath not swelling at ends.

**Notes** *Xylaria eugeniae* is known only from Mexico and grows on the fallen leaves of *Eugenia capuli*. It belongs to the *X*. *filiformis* group and resembles *X*. *filiformis* and *X*. *vagans* but differs by the hyaline sheath surrounding the ascospores not forming a non-cellular appendage at each end.

***Xylaria filiformis*** (Albertini & Schwein.) Fr., Summa Veg. Scand. II, p. 382. 1849. Fig. 4I–N.

≡ *Sphaeria filiformis* Albertini & Schwein, Consp. Fung. (Leipzig), p. 2. 1805; Albertini & Schwein.: Fr., Syst. Mycol. II, p. 329. 1823.

≡ *Hypoxylon filiforme* (Albertini & Schwein.) Rabenh., Deutsch. Krypt. Flor. I, p. 223. 1844.

≡ *Xylosphaera filiformis* (Albertini & Schwein.) Dennis, Kew Bull. 13: 103. 1958.

≡ *Podosordaria filiformis* (Albertini & Schwein.) P. M. D. Martin, J. S. African Bot. 36: 131. 1970, nom. inval. ICN Art. 41.1 (Shenzhen Code); J. S. African Bot. 42: 79. 1976.

Stromata filiform at fertile part, unbranched, with a long acicular apex, on a glabrous stipe, 14–50 mm in total length, 5–8 mm long × ca. 0.7 mm broad at the broadest of fertile part, 6–20 mm long at stipe; surface black, slightly polished, with half-exposed perithecial mounds and fine longitudinal striations, lacking an outer layer, underlain with a thin, soft layer ca. 20 μm thick, concolorous with the surface; interior white, homogeneous, soft. Perithecia depressed-spherical to spherical, 300–400 μm broad × 200–350 μm high. Ostioles papillate to conic-papillate, ca. 70 μm high × 100–130 μm broad at base. Asci not intact, with an apical ring staining blue in Melzer’s iodine reagent, inverted hat-shaped, 2 μm high × 2–2.5 μm broad. Ascospores light brown, unicellular, short fusoid-inequilateral, with narrowly rounded ends frequently pinched on one or both ends, smooth, (9.5–)11.5–13.5(–14.5) × (4–)4.5–5.5(–6) µm (12.5 ± 1.1 × 5.0 ± 0.5 μm, N = 70), with a straight germ slit spore-length on the ventral side, surrounded with a hyaline sheath swelling at both ends to form papillate non-cellular appendages; epispore smooth.

**Specimens examined** FRANCE. on fallen leaves and petioles of *Acer platanoides*?, 1853–1860, *Roberge, M.*, *Desmazières’ Plantes Cryptogames de France sér. II 377*, as *X*. *filiformis* (K[M] 169694, K[M] 169692). GERMANY. Niesky, on leaves, *Schweinitz, L. D. V.* (lectotype of *Sphaeria filiformis* PH ex Schweinitz herb., isolectotypes BPI 799790 ex Schweinitz herb., BPI 799791 ex Schweinitz herb.), immature; Lichterfelde b. Berlin, auf alten Blättern, Aug 1888, *Sydow, P.*, *Sydow’s Mycotheca Marchica 2242*, as *X*. *filiformis* (HBG, S F131054), immature, with developing perithecia. UK. England, Cumbria County, Meathop Wood, on decaying leaves, 28 Jan 1970, *Howard, P. J.*, comm. *Frankland, J.*, as *X*. *filiformis* (K[M] 163347). USA. Georgia, Rabun Co., on fallen leaves of *Ilex paca*, 19 Aug 1932, *Miller, J. H. 4804*, as *X*. *filiformis* (S F131037).

**Notes** Despite that *X. filiformis* has been commonly reported worldwide on fallen leaves, it is likely that this species is largely restricted to Europe. *Xylaria filiformis* as defined herein has uncommonly been collected, and only two of the examined specimens listed above are mature: K(M) 169694 and K(M) 163347, collected from France and the UK, respectively. The former has all the features as described above, while the latter is overmature, with asci and hyaline sheath no longer available. These two specimens formed the basis for *X*. *filiformis* in Dennis (1971) despite of his belief that this species is cosmopolitan.

The specimen S F131037 from Georgia, USA has ascospores (9.5–)10.5–11.5(–12.5) × (3.5–)4–5(–5.5) µm, slightly smaller than those from Europe but otherwise fits *X*. *filiformis* and is tentatively included here. Rogers and Samuels (1986) reported three species resembling *X*. *filiformis* from New Zealand: *X*. cf. *filiformis*, *Xylaria* taxonomic species 1, and *Xylaria* taxonomic species 2, which have ascospores (12–)13–16(–21) × 5–7(–8) µm, (14.5–)16–20(–22) × (6.5–)7–8.5(–9) µm, and 16–17(–19) × 7–8(–8.5) µm, respectively. These three New Zealand species differ from *X*. *filiformis* by having larger ascospores and lacking a hyaline sheath surrounding their ascospores and likely represent undescribed species. *Xylaria* cf. *filiformis* that San Martín et al. (1997) reported differs from typical *X*. *filiformis* mainly by having larger ascospores (12.5–)13–17.5 × 5.5–6 μm.

We studied many American and European specimens labeled as *X*. *filiformis*, most of which are in fact *X*. *simplicissima* from herbaceous stems and only occasionally from fallen leaves of various plants. *Xylaria simplicissima* can easily be separated from *X*. *filiformis* by its larger ascospores (15–)16.5–19(–21.5) × (5–)5.5–6.5(–7.5) µm that lack a hyaline sheath. *Xylaria vagans* highly resembles *X*. *filiformis* but differs primarily by its darker, ellipsoid-inequilateral ascospores and its geographic distribution in the tropics and subtropics.

Albertini and Schweinitz (1805) clearly described and illustrated *X*. *filiformis* as a fungus emerging from fallen leaves in Niesky, Germany. The color drawings indicate that the stromata are sterile, with one of them bearing immature perithecia arranged in close set, half-buried with the upper conical portion exposed. Schweinitz’s specimen in PH and BPI collected from Niesky also confirms its leaf-inhabiting habit.

Desmazières was probably the first person to record ascospores of *X*. *filiformis*, which were given as “0^mm^,015” (= 15 μm) long on the label of his exsiccata *Plantes Cryptogames de France sér. II 377* issued between 1853 and 1860. He also provided a detailed description for the species on this label and mentioned that the exsiccata contained material collected from leaves of *Acer platanoides*, *Carpinus*, *Castanea*, *Fagus*, *Populus*, *Quercus*, and *Salix*. It is thus possible that the Desmazières exsiccata is composed of stromata from multiple gatherings. The two duplicates of the Desmazières exsiccata in K, upon which Dennis (1971) based his interpretation of *X*. *filiformis*, contain light brown to brown ascospores with a prominent non-cellular appendage on each end. Nonetheless, Nitschke (1867) did not find ascospores in the duplicate of this exsiccata that he studied nor in Rabenhorst’s *Fungi Europaei 57* or his own collections and some Kunze specimens of *X*. *filiformis*. According to Nitschke (1867), the Kunze specimens are probably the original material from Albertini and Schweinitz and correspond well to their description and illustration. Fuckel (1869) reported that no ascospores were found in his specimens collected from leaves of various plants around Oestrich, Germany. Ascospores of *X*. *filiformis* were given as 13–14 × 5–6 μm in Karsten (1879), 13–14 × 5–6 μm in Winter (1887), 12–15 × 5 μm in Saccardo (1882), and 12–15 × 4–5 μm in Traverso (1906).

***Xylaria foliicola*** G. Huang & L. Guo, in G. Huang, L. Guo & N. Liu, Mycotaxon 129: 150. 2014.

For a description of the teleomorph, see Huang et al. (2014) where illustrations of stromata, asci, and ascospores are also provided. *Xylaria foliicola* is characterized by the following features: stromata cylindrical at fertile part, unbranched or branched occasionally, with a long acicular apex, on a glabrous stipe, 23–35 mm in total length, 7–18 mm long × 1–2 mm broad at fertile part, with a black surface lacking perithecial mounds or with slight perithecial mounds, overlain with a dull grayish brown peeling layer split into narrow or thread-like stripes, with a white interior; perithecia subspherical to ellipsoid, 400–650 μm broad, with a papillate ostiole; ascospores brown, ellipsoid-inequilateral, with narrowly rounded ends, smooth, (8.5–)9–11 × 4–6 μm, with a straight germ slit spore-length on the ventral side, lacking a hyaline sheath.

**Notes** *Xylaria foliicola* is known only from its type material collected from Yunnan, China (Huang et al. 2014). *Xylaria minuscula* is similar to *X*. *foliicola* in stromata overlain with a narrowly striped outer peeling layer but differs from it mainly by larger ascospores.

***Xylaria heloidea*** Penz. & Sacc., Malpighia 11: 498. 1897.

See Ju et al. (2018) for a description and illustrations. *Xylaria heloidea* is not exclusively associated with fallen leaves and does not appear to have a specificity to certain hosts.

**Fig. 5:**
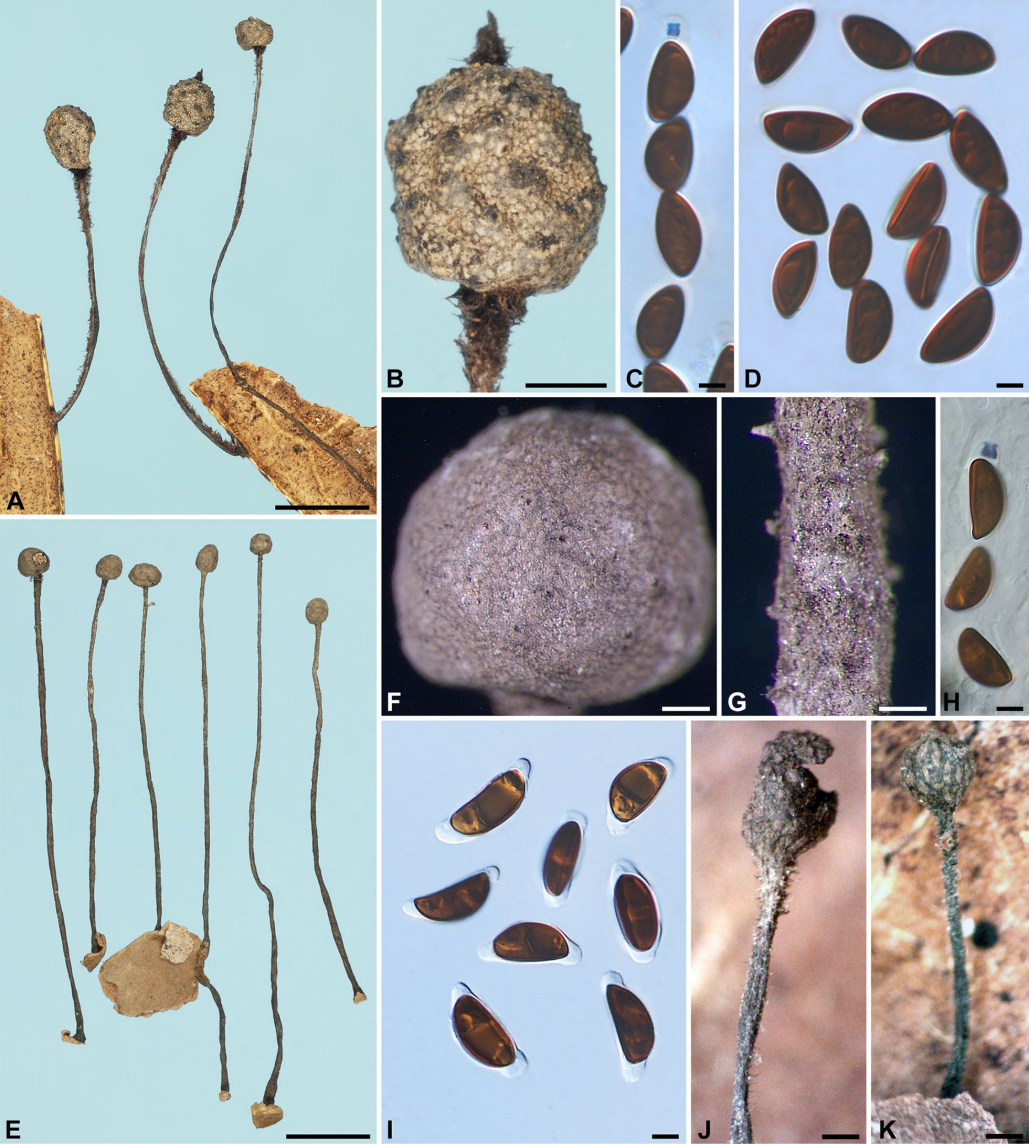
*Xylaria hispidipes*, *X*. *hypsipoda*, and *X*. *imminuta*. **A**–**D** *X*. *hispidipes* (holotype). **A** Stromata. **B** Stromatal surface. **C** Ascal apical ring and ascospores. **D** Ascospores. **E**–**I** *X*. *hypsipoda* (holotype). **E** Stromata. **F**, **G** Stromatal surface. **H** Ascal apical ring and ascospores. **I** Ascospores. **J**, **K** *X*. *imminuta* (holotype), stromata overlain with a thin pellicle that is cracked reticulately into plaques. Bars in **A** = 5 mm; **B** = 1 mm; **E** = 1 cm; **F** = 0.5 mm; **G**, **K** = 0.25 mm; **J** = 0.2 mm; **C**, **D**, **H**, **I** = 5 μm

***Xylaria hispidipes*** Y.-M. Ju & H.-M. Hsieh, sp. nov. Fig. 5A–D.

MycoBank MB848540.

**Typification** INDONESIA. North Sulawesi, Eastern Dumoga-Bone National Park, at confluence of Toraut & Tumpah Rivers, Project Wallace Base Camp, on leaves, Sep–Nov 1985, *Samuels, G. J. GS1981*, as *X. hypsipoda* (holotype of *X. hispidipes* NY 01089626, isotype BO 19978).

**Etymology** Referring to the coarse rigid erect hairs covering the stromatal stipes.

Stromata capitate at fertile part, unbranched, with a mucronate apex, on a long hirsute stipe, 15–40 mm in total length, 1–3.5 mm high × 2–3 mm broad at fertile part, 14–38 mm long at stipe; surface dull grayish brown, lacking perithecial mounds, overlain with a thin pellicle cracked reticulately into plaques 100–200 μm broad, underlain with a carbonized layer 300–400 μm thick; interior white, homogeneous, soft. Perithecia spherical, 300–600 μm broad. Ostioles papillate, ca. 100 μm high × 110–140 μm broad at base. Asci with eight ascospores arranged in uniseriate to partially biseriate manner, cylindrical to clavate, at least 160 μm total length, the spore-bearing part 70–100 μm long × 12–20 μm broad, with an apical ring staining blue in Melzer’s iodine reagent, urn-shaped, 4.5–6 μm high × 3–5 μm broad. Ascospores dark brown, unicellular, ellipsoid-inequilateral, with narrowly rounded ends, smooth, (15–)15.5–17(–18) × (6.5–)7.5–9(–9.5) µm (16.3 ± 0.8 × 8.2 ± 0.6 μm, N = 40), with a straight germ slit spore-length or nearly so on the ventral side, lacking a hyaline sheath; epispore smooth.

Cultures obtained by Rogers et al. (1987) (as *X*. *hypsipoda*) with stromata produced on OA but without an observed anamorph.

**Notes** *Xylaria hispidipes* is similar to *X. hypsipoda* in having a capitate stromatal fertile part overlain with a thin pellicle that is cracked reticulately into plaques on a long stipe. In fact, the type material of *X*. *hispidipes* was previously identified as *X*. *hypsipoda* by Rogers et al. (1987). *Xylaria hypsipoda* can be separated from the current species by its stromata with a rounded fertile apex and a glabrous stipe, less conspicuous ostioles, and smaller ascospores surrounded with a hyaline sheath that swells at both ends. The asci from the type material are now fragmentary; the measurement is thus adopted from Rogers et al. (1987), where colonies on oatmeal agar were described but no anamorph was produced.

***Xylaria hypsipoda*** Massee, Bull. Misc. Inform. Kew nos. 153–154: 174. 1899. Fig. 5E–I.

Stromata capitate at fertile part, unbranched, with a rounded top, on a long glabrous stipe slightly roughened with scars of fallen conidial pegs, (20–)45–70 mm in total length, 2.6–3.4 mm high × 2.7–4 mm broad at fertile part; surface dull grayish brown, lacking perithecial mounds, overlain with a thin pellicle cracked reticulately into plaques 100–200 μm broad, underlain with a carbonized layer 90–100 μm thick; interior white, homogeneous, soft. Perithecia spherical, 500–600 μm broad. Ostioles minutely papillate, ca. 70 μm broad at base. Asci not intact, with an apical ring staining blue in Melzer’s iodine reagent, urn-shaped, 3–3.5 μm high × 3.5 μm broad. Ascospores dark brown, unicellular, ellipsoid-inequilateral, with narrowly to broadly rounded ends, smooth, (13–)13.5–15(–16.5) × (6–)6.5–7.5(–8) µm (14.1 ± 0.8 × 7.0 ± 0.5 μm, N = 40), with a straight germ slit spore-length on the ventral side, surrounded with a hyaline sheath swelling at both ends to form papillate non-cellular appendages; epispore smooth.

**Specimens examined** SINGAPORE. Bukit Mardi, on fallen leaves, 1897, *Ridley, H. N. 34* (holotype of *X. hypsipoda* K[M] 156834). PHILIPPINES. Luzon, Makiling, on dead sheath of *Arenga saccharifera*, 27 Sep 1920, *Sanchez A. 9570*, as *X. hypsipoda* (BPI 713971 ex Lloyd herb. 10074, BPI 585083 ex Reinking herb.).

**Notes** *Xylaria hypsipoda* is unique among foliicolous *Xylaria* species in stromata having a large stature topped by a capitate fertile part. The collection reported as *X*. *hypsipoda* by Rogers et al. (1987) from North Sulawesi, Indonesia is newly described herein as *X*. *hispidipes*. Lloyd (1923) reported a collection of *X*. *hypsipoda* from the Philippines, which was sent to him by O. A. Reinking. He presented a photograph in Fig. 2363 showing two stromata characterized by a globose head on a long slender stipe. However, he did not find spores in the specimen but quoted the spore size as 12 × 6–7 μm, likely provided by Reinking. The specimen (BPI 713971) from the Lloyd herbarium contains only long slender stipes arising from the sheath of *Arenga saccharifera*, without any trace of globose heads. A duplicate of the same collection (BPI 585083) no longer contains any *Xylaria* material.

***Xylaria imminuta*** Y.-M. Ju & H.-M. Hsieh, nom. nov. Fig. 5J, K.

MycoBank MB848541.

≡ *Xylaria hypsipoda* Massee var. *microspora* J. D. Rogers, Callan & Samuels, Mycotaxon 29: 162. 1987.

**Typification** INDONESIA. North Sulawesi, Eastern Dumoga-Bone National Park, at confluence of Toraut & Tumpah Rivers, Project Wallace Base Camp, on leaves, Sep–Nov 1985, *Samuels, G. J. GS1954* (holotype of *X. hypsipoda* var. *microspora* BO 19982).

**Etymology** Referring to its miniature stromata.

Stromata capitate at fertile part, unbranched, with a rounded top, on a long hirsute stipe, 60–85 mm long in total length, 0.6–1.2 mm high × 0.6–0.8 mm broad at fertile part; surface blackish brown, lacking perithecial mounds, overlain with a thin pellicle cracked reticulately into plaques 70–120 μm broad, underlain with a thin black layer ca. 20 μm thick. Perithecia not measured. Ostioles minutely papillate, ca. 50 μm broad at base. Asci not measured. Ascospores as in *X*. *hispidipes* but smaller, 8–9(–9.5) × 4–4.5(–6.6) µm.

**Notes** *Xylaria imminuta* was originally described as *X*. *hypsipoda* var. *microspora* by Rogers et al. (1987) who noted that it is a diminutive form of *X*. *hypsipoda*. The collection identified as *X*. *hypsipoda* by Rogers et al. (1987) is described herein as a new species *X*. *hispidipes*, which differs from *X*. *imminuta* mainly by its larger stromata with a minute sterile apex and larger ascospores (15–)15.5–17(–18) × (6.5–)7.5–9(–9.5) µm.

The holotype at BO is now rather scanty, consisting of less than ten tiny stromata, only three of which still bear a fertile part. As such, we did not exploit the specimen further but instead refer to the ascospore morphology described in Rogers et al. (1987).

***Xylaria kamatii*** Pande, Nova Hedwigia 24: 13. 1973.

For a description of the teleomorph, see Pande (1973) where illustrations of stroma, perithecia, ascus, and ascospores are also provided. *Xylaria kamatii* is characterized by the following features: stromata cylindrical at fertile part, unbranched, with a blunt apex, on a glabrous stipe, 10 mm in total length, 4 mm long × 0.8 mm broad at fertile part, with a black surface where perithecial mounds are inconspicuous, not shrinking upon drying, with a white interior; perithecia spherical to obovoid, 320–480 μm broad × 400–480 μm high, with a slightly papillate ostiole; ascospores brown, ellipsoid-inequilateral, 8–10 × 4–6 μm, with a straight germ slit much less than spore-length, surrounded with a hyaline sheath swelling at both ends to form papillate non-cellular appendages.

**Notes** *Xylaria kamatii* is known only from its type collection, which was made from fallen leaves of *Memecylon umbellatum* (Melastomataceae) in India (Pande 1973). We made a request for loaning the type specimen preserved at AMH but did not receive a response. The protologue indicates that *X*. *kamatii* possesses a short germ slit on its ascospores, which are enclosed by a hyaline sheath. The ascospores in Fig. 2D of Pande (1973) were depicted as having a non-cellular appendage at each end.

**Fig. 6:**
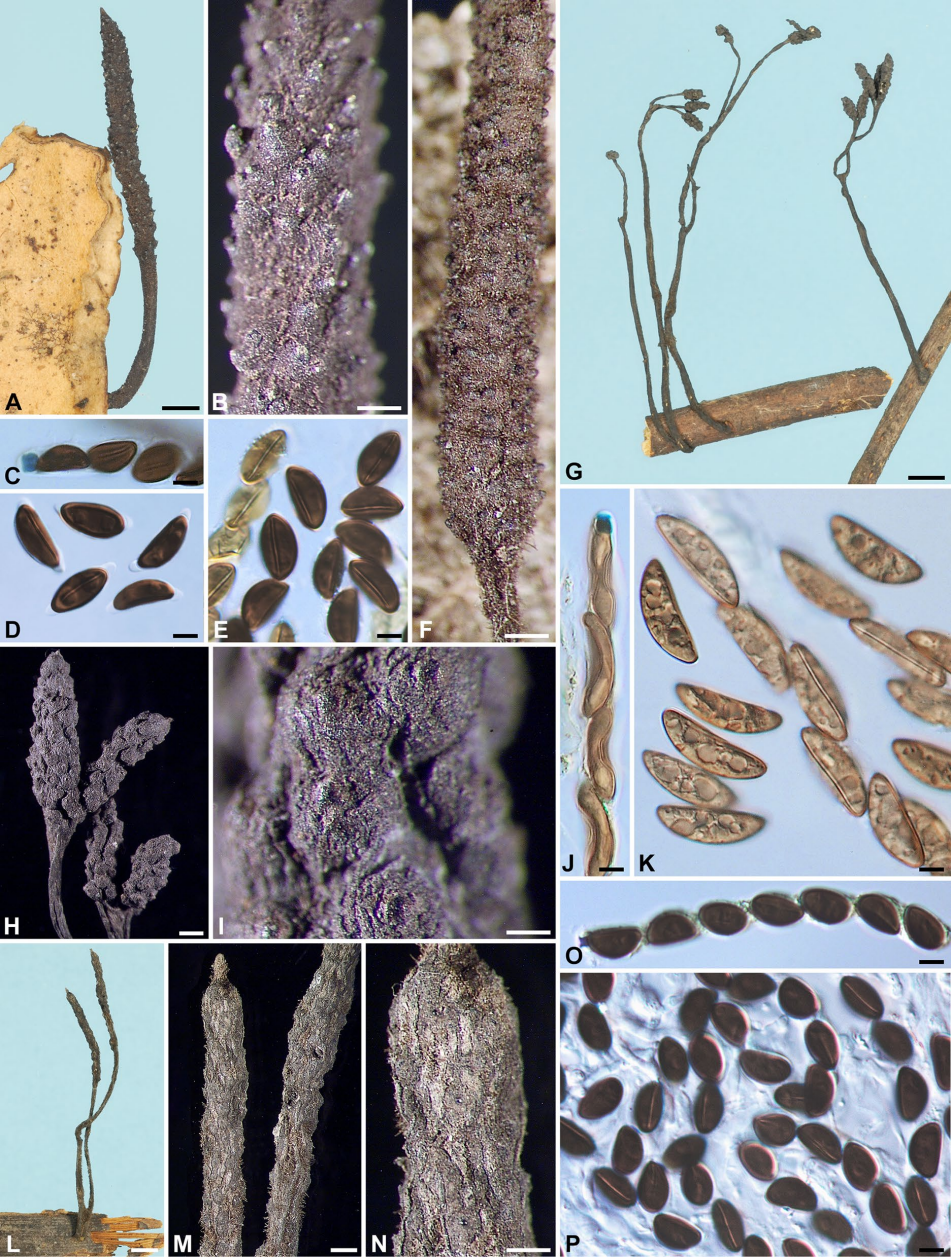
*Xylaria lima*, *X*. *luxurians*, and *X*. *maitlandii*. **A**–** F** *X*. *lima* (holotype except for F, which is from BPI 881412 from French Guiana). **A** Stromata. **B**,** F** Stromatal surfaces coated with a tomentum and showing conic-papillate ostioles that are tilting upwards. **C** Ascal apical ring and ascospores. **D**,** E** Ascospores. **G**–**K** *X*. *luxurians* (isotype HBG). **G**, **H** Stromata. **I** Stromatal surface. **J** Ascal apical ring and ascospores. **K** Ascospores. **L**–**P** *X*. *maitlandii* (holotype). **L** Stromata. **M**, **N** Stromatal surface overlain with a striped hirsute peeling layer. **O** Ascal apical ring and ascospores. **P** Ascospores. Bars in **A**, **H**, **M** = 1 mm; **B**, **I** = 0.25 mm; **F**, **N** = 0.5 mm; **G**, **L** = 5 mm; **C**–**E**, **J**, **K**, **O**, **P** = 5 μm

***Xylaria lima*** Höhn., Denkschr. Kaiserl. Akad. Wiss. Wien, Math.-Naturwiss. Kl. 83: 27. 1907. Fig. 6A–F.

= *Xylaria mexicana* San Martín, J. D. Rogers & P. Lavín, Revta Mex. Micol. 13: 66. 1997.

Stromata cylindrical at fertile part, unbranched, with an acuminate apex, on a hirsute stipe, 11–21 mm in total length, 3.1–7.5 mm long × 0.8–1.1 mm broad at fertile part; surface dark vinaceous brown, with slight perithecial mounds, overlain with a dark brown tomentum, underlain with a thin, soft layer ca. 10 μm thick or less, concolorous with the surface; interior white, homogeneous, soft. Perithecia spherical, 150–250 μm broad. Ostioles conic-papillate, tilting upwards, 90–110 μm high × 90–110 μm broad at base. Asci with eight ascospores arranged in uniseriate manner, cylindrical, 105–145 μm total length, the spore-bearing part 75–105 μm long × 9.5–10.5 μm broad, with an apical ring staining blue in Melzer’s iodine reagent, urn-shaped, 3.5–5 μm high × 3.5–4.5 μm broad. Ascospores brown to dark brown, unicellular, ellipsoid-inequilateral, with narrowly rounded ends, smooth, (10–)10.5–12(–14) × (5–)6–7(–7.5) µm (11.3 ± 0.8 × 6.3 ± 0.5 μm, N = 70), with a straight germ slit spore-length or nearly so on the ventral side, surrounded with a hyaline sheath swelling at both ends to form papillate non-cellular appendages; epispore smooth.

Anamorph was reported by San Martín et al. (1997) (as *X*. *mexicana*) on the surface of immature stromata collected from the field.

**Specimens examined** BRAZIL. São Paulo, Raiz de Serra, on dead leaf, Jun 1901, *von Höhnel, F. 4371* (holotype of *X. lima* FH ex von Höhnel herb.). FRENCH GUIANA. primary forest at km 16 on road between Sinnamary and St. Elie, Orstom Research Area, “Ecerex”, on decaying dicot leaf, 20 Feb–1 Mar 1986, *Samuels, G. J. GS3964*, as *X. appendiculata* (BPI 881412). PANAMA. Canal Zone, Fort Sherman area, on fallen leaves, 5 Aug 1945, *Martin, G. W. 6154*, as *X. appendiculata* (BPI 583953 ex MO 186287).

**Notes** *Xylaria lima* features a tomentose stromatal surface, conic-papillate ostioles tilting upwards, and a non-cellular appendages at each end of the ascospores. It is similar to *X*. *allima*, from which *X. lima* differs primarily by its smaller ascospores.

The protologue of *X. mexicana* suggests that it is in synonym with *X*. *lima*. San Martín et al. (1997) reported the anamorph from immature stromata in nature, where hyaline, smooth, ellipsoid conidia (white in mass) are produced, with a size range (4–)4.5–5 × (1.5–)2(–2.5) µm.

***Xylaria luxurians*** (Rehm) C. G. Lloyd, Mycol. Writings 5: *Xylaria* notes p. 29. 1918. Fig. 6G–K.

*Basionym: Xylaria carpophila* (Pers.) Fr. var. *luxurians* Rehm, Hedwigia 40: 147. 1901.

≡ *Xylosphaera luxurians* (Rehm) Dennis, Kew Bull. 13: 104. 1958.

Stromata cylindrical at fertile part, dichotomously or trichotomously branched once to thrice at stipe, bearing 1–3 clavae on each terminal branch, with a mucronate apex on each clava, on a long, glabrous or hirsute stipe, up to 7 cm in total length, 3–5 mm long × 1.1–2 mm broad at fertile part; surface black, with conspicuous perithecial mounds, overlain with a striped peeling layer, underlain with a thin black layer ca. 20 μm thick; interior white, homogeneous, soft. Perithecia spherical, 450–550 μm broad. Ostioles coarsely papillate, 150–200 μm broad at base. Asci with eight ascospores arranged in uniseriate manner, cylindrical, 130–185 μm total length, the spore-bearing part 95–140 μm long × 9.5–13 μm broad, with an apical ring staining blue in Melzer’s iodine reagent, inverted hat-shaped, 3.5–4.5 μm high × 3–3.5 μm broad. Ascospores light brown to brown, unicellular, ellipsoid-inequilateral to crescentic, with narrowly rounded ends, smooth, (20.5–)21.5–23.5(–24.5) × (6–)6.5–8(–9) µm (22.6 ± 1.1 × 7.4 ± 0.6 μm, N = 40), with a straight to slightly oblique germ slit spore-length or nearly so on the ventral side, lacking a hyaline sheath; epispore smooth.

**Specimens examined** BRAZIL. St. Catharina, Blumenau, probably on fallen petioles, Jul 1888, *Ule*, *E*. *786* (holotype of *X. carpophila* var. *luxurians* S-F62514 ex Rehm herb., isotypes HBG, K[M] [in 2 packets]).

**Notes** *Xylaria luxurians* has stromata with a branched, long stipe that bears one to three clavae on each terminal branch. The photographs shown in Fig. 6G–K were taken from the isotype at HBG, while photographs from the holotype were presented in Ju et al. (2016). One of the two K(M) packets was illustrated by Dennis (1956).

*Xylaria luxurians* was originally published as a variety of *X*. *carpophila*, a fructicolous species quite different from *X*. *luxurians* (Ju et al. 2018). According to the protologue of *X*. *carpophila* var. *luxurians* (Rehm 1901), the type material was collected “in petiolis foliorum?”. Further collections are needed to determine the substrate type and the host range. *Xylaria luxurians* is probably only represented by the type collection thus far. Dennis (1956) cited two specimens for *X*. *luxurians*: one from the type collection and one from *Maggs, D. H. L.C.T.A. 569* from Trinidad (K[M]), which is actually *X*. *meliacearum*. Dennis (1956) also noted that *X*. *luxurians* may be represented by two other specimens: a Venezuelan specimen reported in Miller (1934) and *Bertero 1723* from Chile. We have not examined Miller’s Venezuelan specimen, but Miller (1934) considered it seemingly the same as *X*. *gracillima* sensu Lloyd (1918), which is based on Rick’s Brazilian material. Three specimens identified by Lloyd as *X*. *gracillima* [BRAZIL. *Rick, J.* (BPI 714177 ex Lloyd herb. 10439; BPI 714178 ex Lloyd herb. 182; BPI 714179 ex Lloyd herb. 12636)] were located in his herbarium and redetermined as *X*. *luxurians* by Miller. They have an oblique to slightly sigmoid germ slit on the dorsal side of the ascospores and fit *X*. *chordaeformis* C. G. Lloyd (Ju et al. 2016). It is thus plausible that Miller’s Venezuelan specimen is not *X*. *luxurians* but *X*. *chordaeformis*. The specimen *Bertero 1723* [CHILE. Juan Fernández, ad corticem *Urticae excelsae*, 1830, as *X. hypoxylon* var. *cupressiformis* (K[M] 236761), mistaken as *X. hypoxylon* var. *uniformis* Mont. in Dennis (1956)] is *X*. *zealandica* Cooke.

Two specimens identified as *X*. *luxurians* by C. G. Lloyd [BRAZIL. *Rick, J.* (BPI 714175 ex Lloyd herb. 11859; BPI 714176 ex Lloyd herb. 10462)] are *X*. *chordaeformis*.

***Xylaria maitlandii*** (Dennis) D. Hawksworth, Trans. Br. Mycol. Soc. 61: 199. 1973. Fig. 6L–P.

≡ *Xylosphaera maitlandii* Dennis, Revista Biol. (Lisboa) 1: 181. 1958.

Stromata cylindrical at fertile part, unbranched, with a mucronate apex, on a hirsute stipe, 40–50 mm long in total length, 15–17 mm long × 1.6–1.7 mm broad at fertile part; surface dark brown, lacking perithecial mounds or with slight perithecial mounds, overlain with a striped hirsute peeling layer, underlain with a carbonized layer ca. 40 μm thick; interior white, homogeneous, coriaceous. Perithecia spherical, 300–400 μm broad. Ostioles slightly papillate, 60–80 μm broad at base. Asci with eight ascospores arranged in uniseriate manner, cylindrical, 125–145 μm total length, the spore-bearing part 65–75 μm long × 7–8.5 μm broad, with an apical ring staining blue in Melzer’s iodine reagent, inverted hat-shaped, 2.5–3 μm high × 2.5–3 μm broad. Ascospores dark brown, unicellular, ellipsoid-inequilateral, with broadly rounded ends, smooth, (10–)10.5–11.5(–12) × (5.5–)6–6.5(–7) µm (10.9 ± 0.5 × 6.4 ± 0.3 μm, N = 40), with a straight germ slit spore-length on the ventral side, lacking a hyaline sheath; epispore smooth.

**Specimen examined** UGANDA. Entebbe, Kitubilu Forest, on twigs lying on ground amongst leaf mould, July 1919, *Maitland, T. D.* (holotype of *Xylosphaera maitlandii* K[M] 236752).

**Notes** *Xylaria maitlandii* resembles *X*. *arbuscula* Sacc. and its affinities in having stromata with a mucronate apex and striped outer peeling layer but is unique in having hairs on the outer peeling layer. It remains to be verified if *X. maitlandii* is associated with petioles because the original paper slip enclosed in the packet indicates that the material was collected from twigs. Dennis (1958), however, changed the substrate to fallen petioles in the protologue.

**Fig. 7:**
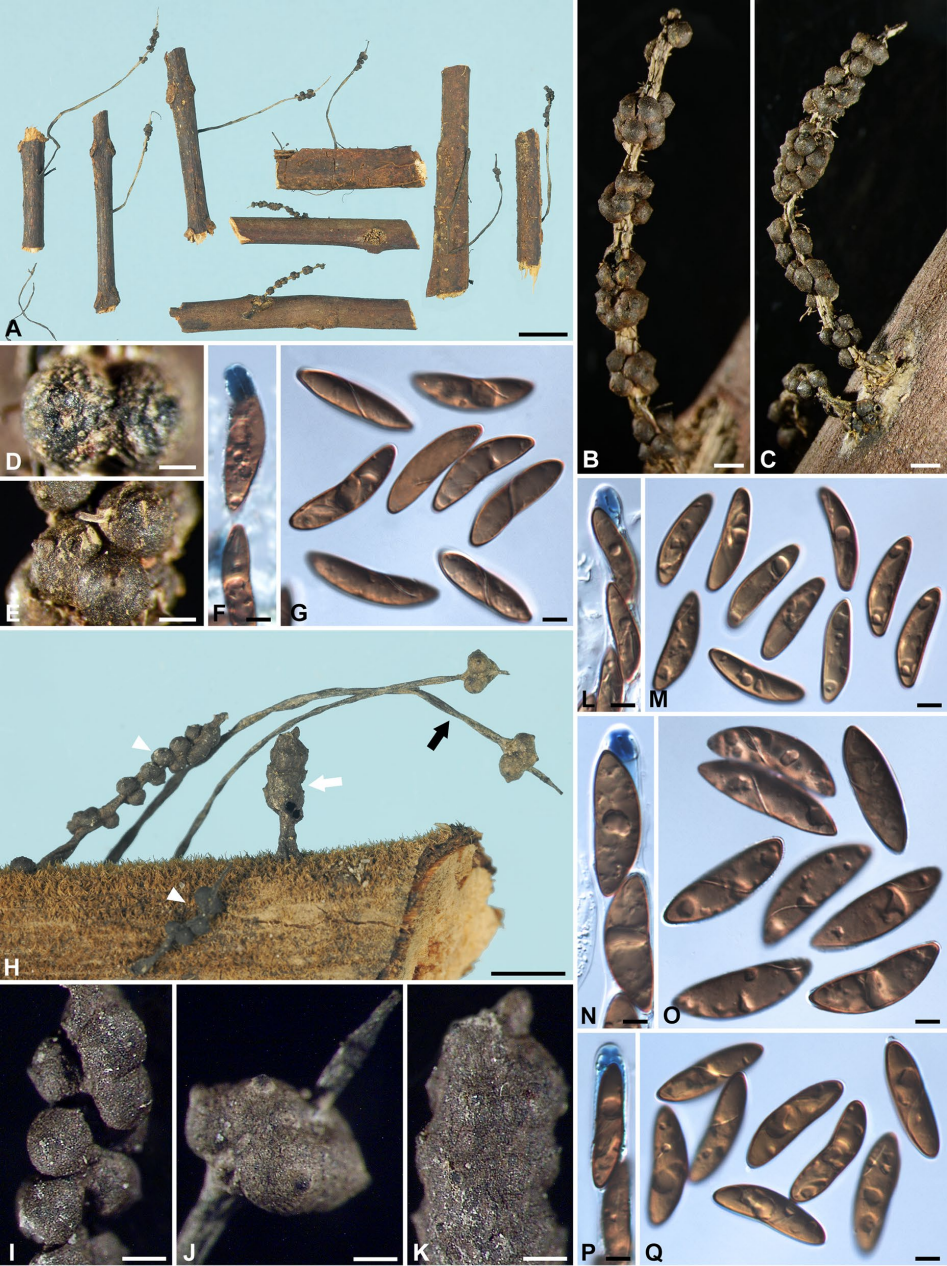
*Xylaria meliacearum*, *X*. sp. GS7461A, and *X*. sp. GS7461B. **A**–**G**,** I**,** L**,** M** *X*. *meliacearum* (**A**–**G** from HAST 145975, **I**, **L**, **M** from HAST 145977). **A**–**C** Stromata. **D**,** E**,** I** Stromatal surfaces. **F** Ascal apical rings and ascospores. **G** Ascospores. **H** Stromata of three *Xylaria* species, with the arrow heads pointing towards that of *X. meliacearum* (HAST 145977), the black arrow pointing towards those of *X*. sp. GS7461A (HAST 145983), and the white arrow pointing towards that of *X*. sp. GS7461B (HAST 145984). **J**, **N**, **O** *X*. sp. GS7461A (HAST 145983). **J** Stromatal surface. **N** Ascal apical ring and ascospores. **O** Ascospores. **K**,** P**,** Q** *X*. sp. GS7461B (HAST 145984). **K** Stromatal surface. **P** Ascal apical ring and ascospores. **Q** Ascospores. Bars in **A** = 1 cm; **B**, **C**, **H** = 1 mm; **D**, **E** = 0.25 mm; **I**–**K** = 0.5 mm; **F**, **G**, **L**–**Q** = 5 μm.

***Xylaria meliacearum*** Læssøe, in Læssøe & Lodge, Mycologia 86: 441. 1994. Fig. 7A–H, I, L, M.

Stromata filiform at fertile part, unbranched, with an acute apex, on a glabrous stipe, 17–65 mm long in total length, 3–17 mm long × 1.6–2.5 mm broad at fertile part; surface sulfur yellow at axis but blackish brown at perithecial mounds, with 2/3-exposed to fully exposed perithecial mounds, overlain with a sulfur-yellow outer layer attached with synnematal remnants at places, gradually ruptured by perithecial mounds into flaky remnants and sloughing off afterwards, underlain with a thin black layer ca. 20 μm thick; interior white, homogeneous, soft. Perithecia spherical, 500–800 μm broad. Ostioles papillate, 120–140 μm broad at base. Asci with eight ascospores arranged in uniseriate manner, cylindrical, 180–270 μm total length, the spore-bearing part 120–170 μm long × 7–8 μm broad, with an apical ring staining blue in Melzer’s iodine reagent, urn-shaped to cylindrical with 1–2 constrictions, 5.5–7 μm high × 3.5–4.5 μm broad. Ascospores brown, unicellular, ellipsoid-inequilateral, with narrowly rounded ends, smooth, (19–)21.5–27.5(–31.5) × (5–)5.5–7(–8) µm (24.5 ± 3.1 × 6.2 ± 0.7 μm, N = 108), with a spiral germ slit spore-length or slightly less than spore-length on the ventral side, lacking a hyaline sheath; epispore smooth.

Cultures and anamorph were reported by Læssøe and Lodge (1994). Stromata were produced on OA with the anamorph formed on stromatal initials. The anamorph formed on the stromata collected from the field is located on the stromatal surface as synnemata or as a continuous layer.

**Specimens examined** FRENCH GUIANA. Paul Isnard Area, Mt. Decou Decou, on twig, 11–12 Mar 1986, *Samuels, G. J. & Searwar, P. GS4251*, as *X*. *meliacearum* (HAST 145976 ex NY); ca. 10 km SW of Saul, track from Saul leading SW toward Mt. Galbao, elev. ca. 200 m, on twig, 7–9 Jan 1986, *Samuels, G. J. & Boise, J. GS2600*, as *X*. *meliacearum* (BPI 881642); ca. 15 km SW of Saul, along trail from Saul to Mt. Galbao, alt. 600 m, on dead twigs, 14–29 Jan 1986, *Samuels, G. J. & Boise, J. GS2879*, as *X*. *meliacearum* (HAST 145975 ex NY); ca. 17.5 km SW of Saul, Mt. Galbao, Camp 3, alt. 350 m, on leaf rachis, 24–26 Jan 1986, *Samuels, G. J. & Boise, J. GS3203*, as *X*. *meliacearum* (BPI 881654). PUERTO RICO. Luquillo Mts., El Verde Research Area, on petioles of *Guarea guidonia*, 16 Oct 1992, *Lodge, D. J. PR894* (paratype of *X*. *meliacearum* WSP ex Rogers herb.). TRINIDAD AND TOBAGO. Trinidad, Heights of Aripo, on dead wood, 11 Jun 1945, *Maggs, D. H. L.C.T.A. 569*, as *X. luxurians* (K[M]). VENEZUELA. Edo. Trujillo, Parque Nacional Guaramacal, ca. 10 km SW of Batatal, La Defensa, along Río Saguás, Campamiento Granja Bocono, in forest along trail to water source, alt. 2000 m, on petiole of *Cecropia* (Urticaceae)?, 20, 23 Nov 1990, *Samuels, G. J., Hein, B. & Huhndorf, S. M. GS7461*, as *X*. *meliacearum* (HAST 145977 ex BPI).

**Notes** *Xylaria meliacearum* belongs to the *X*. *filiformis* group and differs from its species mainly by larger ascospores that have a spiral germ slit. It is noteworthy that the anamorph is born on immature stromata as upright, sulfur-yellow synnematal pegs and becomes synnematal remnants that can still be observed adhering to the outer stromatal layer. The ascospore size range of *X*. *meliacearum* is fairly broad, much as given by Læssøe and Lodge (1994) where the ascospores were measured “(18.5–)19.1–30.0(–33.0) × (4.0–)4.6–6.6(–7.9) µm (means from various collections 20.1–29.7 × 4.7–7.5 µm)”.

The Venezuelan collection GS7461 is mixed with two other species, *X*. sp. GS7461A and *X*. sp. GS7461B, on the same petiole (Fig. 7H). All of these three species have a spiral germ slit on their ascospores but differ by the combinations of stromatal morphology and ascospore size range.

***Xylaria memecyli*** Pande, Nova Hedwigia 24: 14. 1973, as “*memecylonii*.”

For a description of the teleomorph, see Pande (1973) where illustrations of stromata, perithecia, asci, and ascospores are also provided. *Xylaria memecyli* is characterized by the following features: stromata peltate at fertile part, concave underneath, unbranched, convex on top, on a long, glabrous stipe, 10–15 mm in total length, 1 mm high × 2 mm broad at fertile part, with a dark brown to blackish surface lacking perithecial mounds, with a whitish to pale yellow interior; perithecia spherical to obovoid, 400–480 μm broad × 512–590 μm high; ascospores brown, ellipsoid-inequilateral, 12–14 × 6–8 μm, with a straight germ slit spore-length, surrounded with a hyaline sheath swelling at both ends to form papillate non-cellular appendages.

**Notes** *Xylaria memecyli* is known only from the type collection, which was made from fallen leaves of *Memecylon umbellatum* (Melastomataceae) in India (Pande 1973).We made a request for a loan of the type preserved at AMH but did not receive a reply. Although certain important features, such as stromatal surface, perithecial aggregation in stromata, ostioles, and certain ascospore traits, were not described in the protologue, the stromatal shape and ascospore morphology suggest that *X*. *memecyli* is a distinct species. The stromatal size was not given by Pande (1973); the measurement provided above was made from the two stromata on a leaf presented in Fig. 2 by Pande (1973), which were sketched in natural habit as mentioned in the figure legend.

**Fig. 8:**
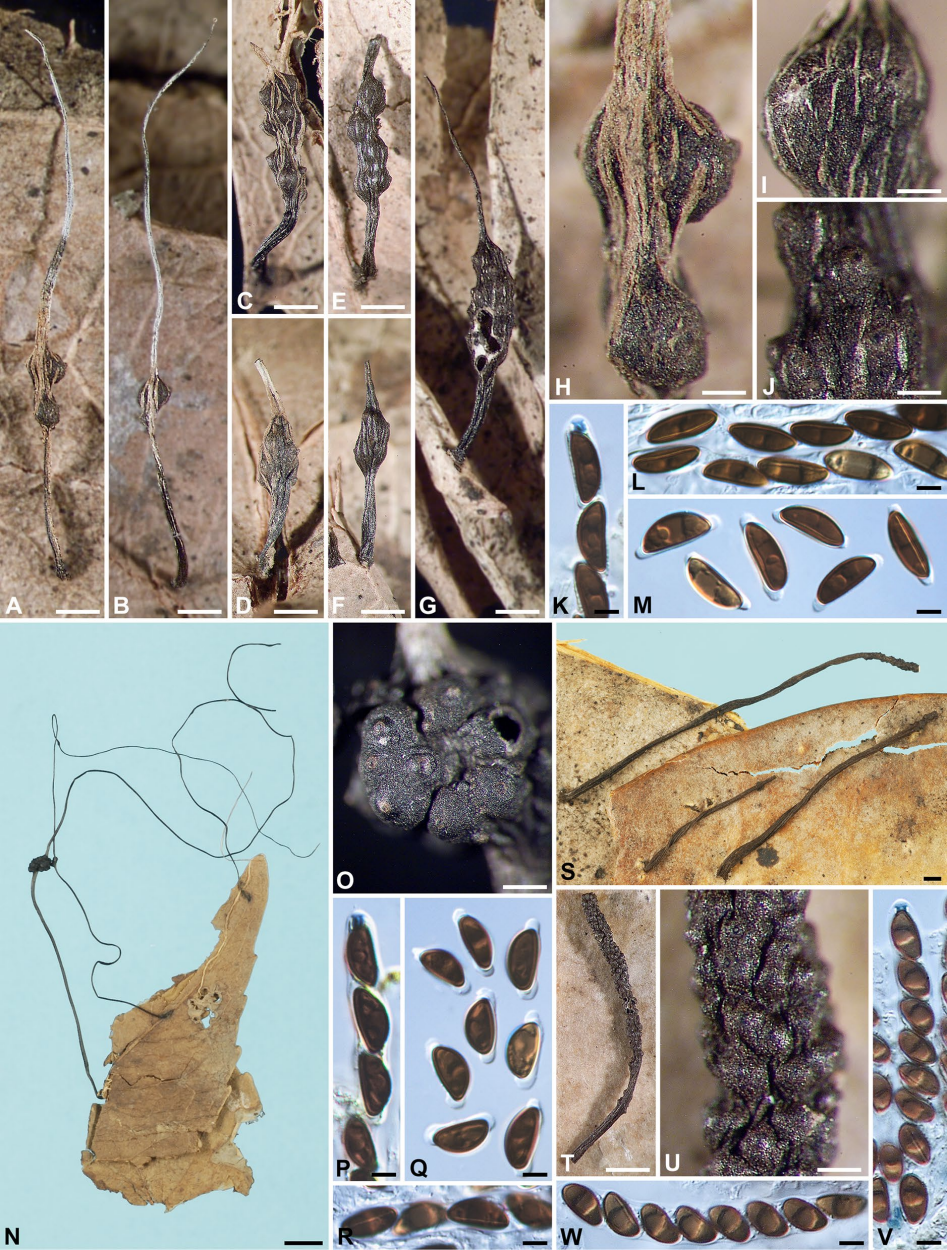
*Xylaria minuscula*, *X*. *nainitalensis*, and *X*. *neblinensis*. **A**–**M** *X*. *minuscula* (holotype). **A**–**G** Stromata. **H**–**J** Stromatal surfaces. **K** Ascal apical ring and ascospores. **L**,** M** Ascospores. **N**–**R** *X*. *nainitalensis* (isotype K[M] 236748). **N** Stromata. **O** Stromatal surface. **P** Ascal apical ring and ascospores. **Q**, **R** Ascospores. **S**–**W** *X*. *neblinensis* (holotype). **S**, **T** Stromata. **U** Stromatal surface. **V** Ascal apical ring and ascospores. **W** Ascospores. Bars in **A**–**G**, **S** = 1 mm; **H**–**J**, **U** = 0.25 mm; **N** = 5 mm; **O** = 0.5 mm; **T** = 2 mm; **K**–**M**, **P**–**R**, **V**, **W** = 5 μm

***Xylaria minuscula*** Y.-M. Ju & H.-M. Hsieh, sp. nov. Figs. 8A–M, 10E, 12 K, L.

MycoBank MB848542.

**Typification** TAIWAN. I-lan Co., Yuan-shan, Fu-shan, on dead leaves of *Castanopsis carlesii* var. *sessilis*, 27 Oct 2001, *Ju, Y.-M. & Hsieh, H.-M. 90102701* (cultured) (holotype of *X. minuscula* HAST 145978), GenBank: ITS = OQ883721.

**Etymology** Referring to its minute stromata.

Stromata cylindrical at fertile part, unbranched, with a long acicular apex, on a glabrous stipe, 3–14 mm in total length, 1–5 mm long × 0.6–1.1 mm broad at fertile part; surface black, with conspicuous to half-exposed perithecial mounds, overlain with a dull grayish brown peeling layer split into narrow or thread-like stripes, underlain with a thin black layer ca. 30 μm thick; interior white, homogeneous, soft. Perithecia spherical, 300–500 μm broad. Ostioles coarsely papillate, 110–120 μm high × 180–190 μm broad at base. Asci with eight ascospores arranged in uniseriate manner, cylindrical, 130–180 μm total length, the spore-bearing part 80–95 μm long × 7–8 μm broad, with an apical ring staining blue in Melzer’s iodine reagent, inverted hat-shaped, 2.5–3 μm high × 2.5–3 μm broad. Ascospores brown to dark brown, unicellular, ellipsoid-inequilateral, with narrowly rounded ends bearing a tiny hyaline cellular appendage on one end, smooth, (13.5–)14–15(–17) × (4.5–)5–6(–7) µm (14.5 ± 0.8 × 5.7 ± 0.5 μm, N = 56), with a straight germ slit spore-length on the ventral side, surrounded with a hyaline sheath swelling at both ends to form papillate non-cellular appendages; epispore smooth.

Cultures and anamorph. Colonies reaching the edge of 9-cm Petri dish in 3 wk, white, velvety, appressed, azonate, with diffuse margins. Reverse uncolored. Stromata arising at periphery, conical, unbranched, 1–2 mm high × 1–2 mm diam at base, white, immediately becoming black from base upwards. Anamorph not produced.

**Additional specimens examined** MALAYSIA. Sarawak, on dead leaves, *Beccari, O.*, as *X. phyllocharis* (K[M] 236749 ex Cooke herb.), mixed with the lectotype of *X. phyllophila*. TAIWAN. Nan-tou County, Yu-chee, Lien-hwa-chee, on fallen leaves, 20 Aug 1991, *Ju, Y.-M. 80082005*, as *X*. sp. (WSP).

**Notes** *Xylaria minuscula* was reported as an unnamed *Xylaria* sp. (80082005) from Taiwan by Ju and Rogers (1999) who suspected that *X. phyllophila* might be a tenable name for it. However, *X. phyllophila* differs from *X*. *minuscula* mainly by having larger ascospores (15–)16.5–18(–19) × (7.5–)8–9(–9.5) µm. *Xylaria minuscula* resembles *X. foliicola* in stromatal morphology but differs by its ascospores having a larger size range, i.e., (13.5–)14–15(–17) × (4.5–)5–6(–7) µm vs. (8.5–)9–11 × 4–6 μm, and being enclosed within a hyaline sheath.

***Xylaria nainitalensis*** Dargan, J. Bio. Res. 3: 43. 1983. Fig. 8N–R.

Stromata capitate at fertile part, unbranched, with a long acicular apex, on a glabrous stipe, 91–147 mm in total length, 1.3–2.9 mm long × 1–2.7 mm broad at fertile part; surface black, with conspicuous perithecial mounds, lacking an outer layer, underlain with a thin, black layer ca. 20 μm thick; interior white, homogeneous, soft. Perithecia spherical, 350–450 μm broad. Ostioles coarsely papillate, 90–130 μm high × 150–180 μm broad at base. Asci with eight ascospores arranged in uniseriate manner, cylindrical, 150–180 μm total length, the spore-bearing part 80–95 μm long × 7–8.5 μm broad, with an apical ring staining blue in Melzer’s iodine reagent, inverted hat-shaped, 2.5–3 μm high × 2.5–3 μm broad. Ascospores brown to dark brown, unicellular, ellipsoid-inequilateral, with narrowly rounded ends sometimes bearing a tiny hyaline cellular appendage on one end, smooth, (10.5–)11.5–13.5(–15) × (5–)5.5–6.5(–7.5) µm (12.3 ± 1.0 × 6.2 ± 0.5 μm, N = 50), with a straight germ slit spore-length on the ventral side, surrounded with a hyaline sheath swelling at both ends to form papillate non-cellular appendages; epispore smooth.

**Specimens examined** INDIA. on twig, *Narula, A. M. 313*, as *X*. *nainitalensis* (WSP ex Rogers herb.); Uttar Pradesh, Nainital, Sat Tal, on dead leaves of *Quercus incana*, 17 Aug 1973, *Dargan, J. S. 13117* (isotype of *X. nainitalensis* K(M) 236748).

**Notes** *Xylaria nainitalensis* features a capitate stromatal fertile part with conspicuous perithecial mounds on the surface and terminating into a long acicular apex. Its ascospores enclosed within a hyaline sheath that forms a non-cellular appendage at each end. A tiny cellular appendage can sometimes be seen on one end of the ascospores.

A specimen [USA. Florida, San Felasco Reserve, on fallen leaves and petioles, 10 Aug 1985, *Rogers, J. D.*, as *X.* cf. *filiformis* (WSP ex Rogers herb.)], bearing a capitate stromatal fertile part that terminates into a long thin apex, looks much like *X*. *nainitalensis* but is immature.

***Xylaria neblinensis*** Y.-M. Ju & H.-M. Hsieh, sp. nov. Fig. 8S–W.

MycoBank MB848544.

**Typification** VENEZUELA. Territorio Federal Amazonas, Cerro de la Neblina, 6.2 km NE Pico Phelps, alt. 1390–1515 m, on fallen leaves of *Clusia* sp., 22 Feb 1985, *Rossman, A. Y. AR2233*, as *X*. cf. *brachiata* (holotype of *X. neblinensis* BPI 583728, isotype K[M]).

**Etymology** Referring to the collecting site Cerro de la Neblina, Venezuela.

Stromata cylindrical at fertile part, unbranched, with an acute apex, on a glabrous stipe, 15–30 mm in total length, 8–12 mm high × 0.6–0.8 mm broad at fertile part; surface blackish brown, with conspicuous perithecial mounds, lacking an outer layer, with the layer immediately beneath the surface extremely thin, less than 10 μm thick; interior white, homogeneous, soft. Perithecia spherical, 200–300 μm broad. Ostioles papillate, 60–80 μm broad at base. Asci with eight ascospores arranged in uniseriate manner, cylindrical, 80–95 μm total length, the spore-bearing part 55–65 μm long × 7.5–8.5 μm broad, with an apical ring staining blue in Melzer’s iodine reagent, inverted hat-shaped, 2–2.5 μm high × 2–2.5 μm broad. Ascospores brown to dark brown, unicellular, ellipsoid-inequilateral, with narrowly rounded to broadly rounded ends, smooth, (9–)9.5–10.5(–11.5) × (5–)5.5–6(–6.5) µm (10.2 ± 0.5 × 5.6 ± 0.3 μm, N = 40), with a straight germ slit spore-length on the ventral side, lacking a hyaline sheath; epispore smooth.

**Notes** *Xylaria neblinensis* is in general similar to *X*. *appendiculatoides* in stromatal morphology, having a blackish brown surface with conspicuous perithecial mounds and a pointed apex. The ascospores of *X*. *neblinensis* differ from those of *X*. *appendiculatoides* by having a smaller size range and lacking a hyaline sheath.

Rogers et al. (1988) tentatively referred to this fungus as *X.* cf. *brachiata* Sacc. but noted that this fungus was not known to them. A larger ascospore size of 14.5–16 × 6.5–7.5 μm was given in Rogers et al. (1988), where specimens AR2232 and AR2284 were also cited for this fungus, in addition to AR2233. It is possible that larger ascospores can be found in these two collections. The notes enclosed in the packet of AR2233 indicate that the ascospore size is much like our measurement. Although the stromatal apex is currently broken off, an acute stromatal apex was nicely depicted by A. Rossman on the notes.

**Fig. 9:**
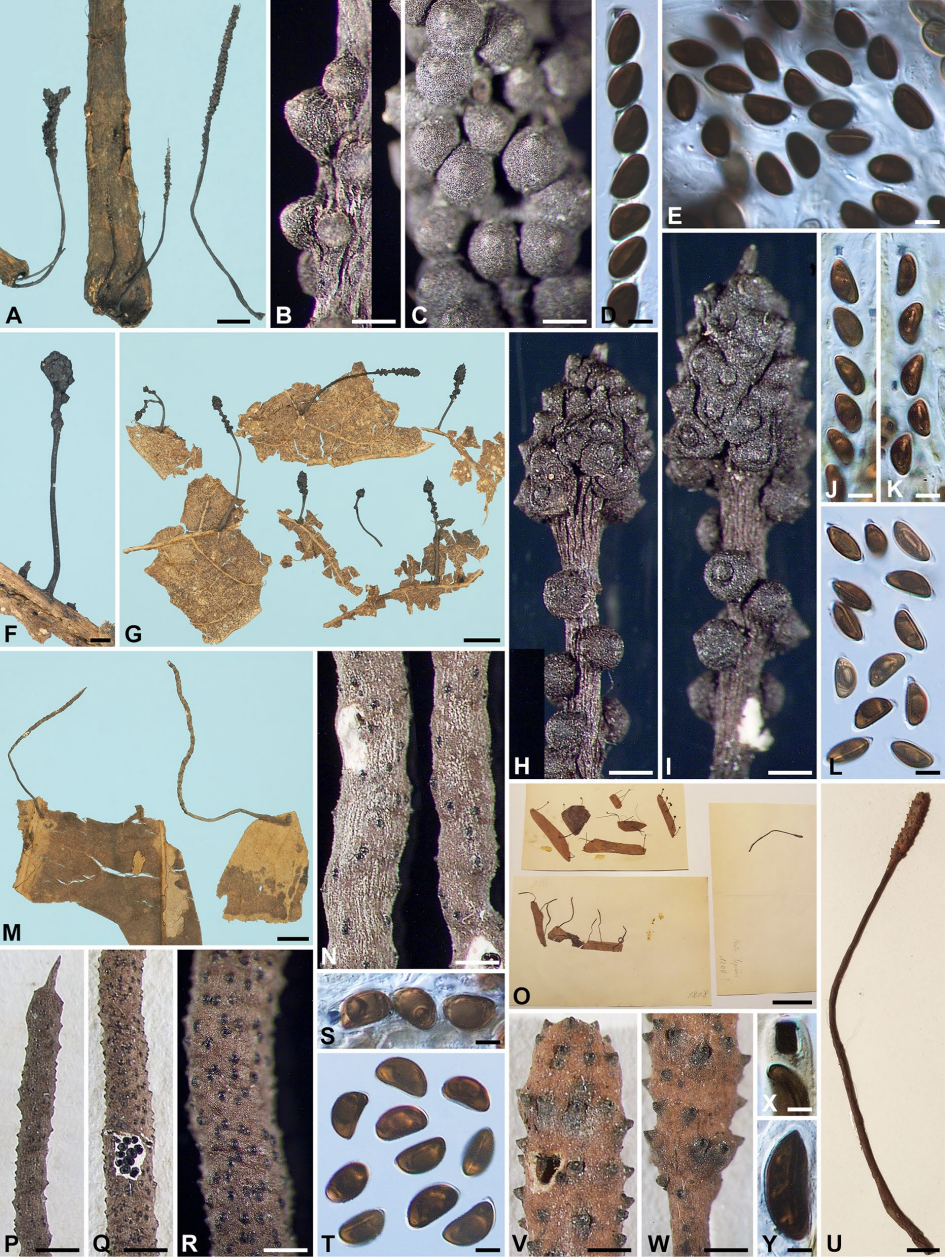
*Xylaria noduliformis*, *X*. *petchii*, *X*. *phyllocharis*, and *X*. *spiculaticlavata*. **A**–**E** *X*. *noduliformis* (holotype). **A** Stromata. **B**, **C** Stromatal surfaces. **D** Ascal apical ring and ascospores. **E** Ascospores. **F**–**L** *X*. *petchii* (**F** holotype; **G**–**L** isotype K[M] 169689). **F**, **G** Stromata. **H**, **I** Stromatal surfaces. **J**, **K** Ascal apical rings and ascospores. **L** Ascospores. **M**–**T** *X*. *phyllocharis* (**M**, **N** holotype; **O**–**T** isotype PC 0086071). **M**, **O**, **P**, **Q** Stromata; three species are present in O, with *X. aristata* on the upper left, *X. phyllocharis* on the lower left, and *X*. *spiculaticlavata* on the right. **N**, **R** Stromatal surfaces. **S** Ascal apical ring and ascospores. **T** Ascospores. **U–Y** *X*. *spiculaticlavata* (holotype). **U** Enlargement of the stroma on the right in O. **V**, **W** Stromatal surfaces showing coarsely conical ostioles that are slightly tilting upwards. **X** Ascal apical ring and ascospore. **Y** Ascospore. Bars in **A**, **G**, **M**, **U** = 5 mm; **B**, **C**, **H**, **I**, **N**, **R** = 0.5 mm; **F**, **V**, **W** = 1 mm; **O** = 5 cm; **P**, **Q** = 1 mm; **D**, **E**, **J**–**L**, **S**, **T**, **X**, **Y** = 5 μm

***Xylaria noduliformis*** Y.-M. Ju & H.-M. Hsieh, nom. nov. Fig. 9A–E.

MycoBank MB848545.

≡ *Xylosphaera maitlandii* Dennis var. *nuda* Dennis, Revista Biol. (Lisboa) 1: 183. 1958.

≡ *Xylaria maitlandii* (Dennis) D. Hawksworth var. *nuda* (Dennis) D. Hawksworth, Trans. Br. Mycol. Soc. 61: 200. 1973.

**Typification** UGANDA. Entebbe, Kitubilu Forest, on twigs lying on ground amongst leaf mold, Jun 1919, *Maitland, T. D. 484A* (holotype of *Xylosphaera maitlandii* var. *nuda* K[M] 236727).

**Etymology** Referring to the stromatal surface with fully exposed perithecial mounds.

Stromata cylindrical at fertile part, unbranched or branched once at clavae, with an acuminate or mucronate apex, on a glabrous stipe, 29–53 mm in total length, 9–22 mm long × 1.2–2.7 mm broad at fertile part; surface black, with fully exposed perithecial mounds, overlain with a brown striped peeling layer obscured with the perithecial mounds fully exposed, underlain with a thin, black layer ca. 30 μm thick; interior white, homogeneous, soft. Perithecia spherical, 400–550 μm broad. Ostioles slightly papillate, often encircled with a paler area, 50–80 μm broad at base. Asci with eight ascospores arranged in uniseriate manner, cylindrical, 180–210 μm total length, the spore-bearing part 70–80 μm long × 7.5–8.5 μm broad, with an apical ring staining deep blue in Melzer’s iodine reagent, inverted hat-shaped, 2–2.5 μm high × 2–2.5 μm broad. Ascospores dark brown to blackish brown, unicellular, ellipsoid-inequilateral, with narrowly to broadly rounded ends, smooth, (9–)9.5–10.5(–11) × (5.5–)6–6.5(–7) µm (10.0 ± 0.5 × 6.0 ± 0.2 μm, N = 40), with a straight germ slit spore-length on the ventral side, lacking a hyaline sheath; epispore smooth.

**Notes** *Xylaria noduliformis* is only known from the type material and is characterized by half-exposed, highly crowded perithecial mounds. Dennis (1958) recorded *X*. *noduliformis* (as *Xylosphaera maitlandii* var. *nuda*) from the same kind of fallen petioles where the original material of *X*. *maitlandii* was collected. However, like *X. maitlandii*, *X*. *noduliformis* may not be associated with petioles because the original paper slip enclosed in the type packet of *X*. *maitlandii* indicates twigs rather than petioles as the substrate.

***Xylaria petchii*** C. G. Lloyd, Mycol. Writings 7: 1310. 1924. Fig. 9F–L.

= *Xylaria filiformoidea* Hladki & A. I. Romero, Fungal Diversity 42: 83. 2010.

Stromata capitate to short-cylindrical at fertile part, unbranched or branched occasionally at stipe, with an apiculate apex, on a glabrous stipe, 7.5–20 mm in total length, 2–5 mm high × 1–2 mm broad at fertile part; surface black, somewhat polished, with conspicuous to half-exposed perithecial mounds clumped together on upper part, frequently with several loose, fully exposed perithecial mounds scattered below, lacking an outer layer, underlain with a thin, soft layer ca. 10 μm thick or less, concolorous with the surface; interior white, homogeneous, soft. Perithecia spherical, 400–500 μm broad. Ostioles conic-papillate, 110–150 μm high × 150–190 μm broad at base. Asci with eight ascospores arranged in uniseriate manner, cylindrical, 95–125 μm total length, the spore-bearing part 55–70 μm long × 6–7 μm broad, with an apical ring staining blue in Melzer’s iodine reagent, inverted hat-shaped, 2–2.5 μm high × 2–2.5 μm broad. Ascospores brown to dark brown, unicellular, ellipsoid-inequilateral, with narrowly to, less frequently, broadly rounded ends, smooth, (7.5–)8.5–9.5(–10) × (3.5–)4–4.5(–5) µm (8.9 ± 0.5 × 4.4 ± 0.2 μm, N = 40), with a straight germ slit spore-length on the ventral side, surrounded with a hyaline sheath swelling at both ends to form papillate non-cellular appendages; epispore smooth.

**Specimens examined** SRI LANKA. Peradeniya, on fallen leaves, 29 Jan 1919, *Petch, T. 6126* (holotype of *X. petchii* BPI 714305 ex Lloyd herb. 10407, isotype K[M] 169689).

**Notes** *Xylaria petchii* is unique in having most perithecia compacted together near the apex of stromata with several loosely scattered below. Petch collected this fungus and sent two stromata to Lloyd, as shown in Fig. 2960 in Lloyd (1924), upon which Lloyd’s description of *X*. *petchii* was based. The holotype packet now contains only one stroma, while the isotype packet at K contains seven stromata. The stromatal habit of *X*. *petchii* is also shown in *X. filiformoidea*, which is described from Argentina with ascospores 8–9 × 4–5 μm and enclosed within a hyaline sheath (Hladki and Romero 2010). *Xylaria filiformoidea* is inseparable from *X*. *petchii*, and these two names are thus considered in synonymy. *Xylaria petchii* is briefly noted in Ju et al. (2016).

***Xylaria phyllocharis*** Mont., Ann. Sci. Nat., Bot., sér. IV, 3: 108. 1855. Fig. 9M–T.

≡ *Xylosphaera phyllocharis* (Mont.) Dennis, Kew Bull. 13: 105. 1958.

Stromata cylindrical at fertile part, unbranched, with an acuminate to mucronate apex, on a glabrous stipe, 11–40 mm in total length, 5–16 mm long × 0.7–1.3 mm broad at fertile part; surface dark vinaceous brown, with slight perithecial mounds, lacking an outer layer, underlain with a thin, soft layer ca. 30 μm thick, concolorous with the surface; interior white, homogeneous, soft. Perithecia spherical, 100–250 μm broad. Ostioles papillate, 80–120 μm broad at base. Asci with eight ascospores arranged in uniseriate manner, cylindrical, 95–130 μm total length, the spore-bearing part 65–85 μm long × 7–9 μm broad, with an apical ring staining blue in Melzer’s iodine reagent, short-cylindrical to urn-shaped, 2.5–3.5 μm high × 2.5–3.5 μm broad. Ascospores brown to dark brown, unicellular, ellipsoid, strongly inequilateral, with broadly rounded ends sometimes bearing a tiny hyaline cellular appendage on one end, smooth, (9–)10–11.5(–12.5) × (4.5–)5.5–6.5(–7) µm (10.6 ± 0.7 × 6.0 ± 0.6 μm, N = 100), with a straight germ slit spore-length on the ventral side, lacking a hyaline sheath; epispore smooth.

Cultures and anamorph were reported by San Martín et al. (1997) with obovoid to ellipsoid conidia 4–5 × 2.5–3 μm.

**Specimens examined** FRENCH GUIANA. on fallen leaves, *Leprieur, F. R. 1208* (holotype of *X. phyllocharis* PC 0086048 ex Montagne herb., isotype PC 0086071 ex Leprieur herb.); ca. 15 km SW of Saul, along trail from Saul to Mt. Galbao, wet forest with moss and epiphyte covered trees, alt. 600–650 m, on dead leaves, 14–29 Jan 1986, *Samuels, G. J. & Boise, J. GS2962* (HAST 145979 ex NY); Saul, Monts La Fumee, dry primary forest, alt. ca. 400 m, on dead leaves, 4–6 Feb 1986, *Samuels, G. J. & Boise, J. GS3519* (HAST 145980 ex NY). FRENCH WEST INDIES. Guadeloupe, Trace de Sofaïa, on dead leaves, 1 Sep 2005, *Lechat, C. L. 5302* (HAST 145981 ex LIP), GenBank: ITS = GU322445; Martinique, Morne Rouge, La Propreté forest track, on dead leaves in rainforest, 6 Jun 2014, *Fournier, J. MJF 14080* (HAST 145152 ex LIP).

**Notes** *Xylaria phyllocharis* can easily be confused with *X*. *appendiculata* due to their similar stromatal morphologies and ascospore size ranges. However, *X. appendiculata* can be distinguished from *X*. *phyllocharis* by its more conspicuous, conic-papillate ostioles and its ascospores that are not strongly inequilateral and are surrounded by a hyaline sheath that swells on both ends. Fournier et al. (2020) reported *X*. *phyllocharis* from French West Indies and acknowledged variation in ascospore size and shape as well as in the presence or absence of a hyaline sheath among examined specimens; it is likely that some of these specimens are assignable to *X*. *appendiculata*. Like the stromatal surfaces of *X*. *phyllocharis* and *X*. *appendiculata*, those of *X. lima* and *X*. *allima* also have a vinaceous tinge; however, those of the latter two species are entirely tomentose.

The holotype in the Montagne herbarium (PC) is not fully mature, with the stromatal surface still overlain with some powdery remnants, but the isotype from the Leprieur herbarium (PC) is fully mature. The isotype packet from the Leprieur herbarium contains three different *Xylaria* species, each glued onto a separate paper card as shown in Fig. 9O. *Xylaria aristata* is on the upper left, *X. phyllocharis* is on the lower left, and the newly described *X*. *spiculaticlavata* is on the right.

**Fig. 10:**
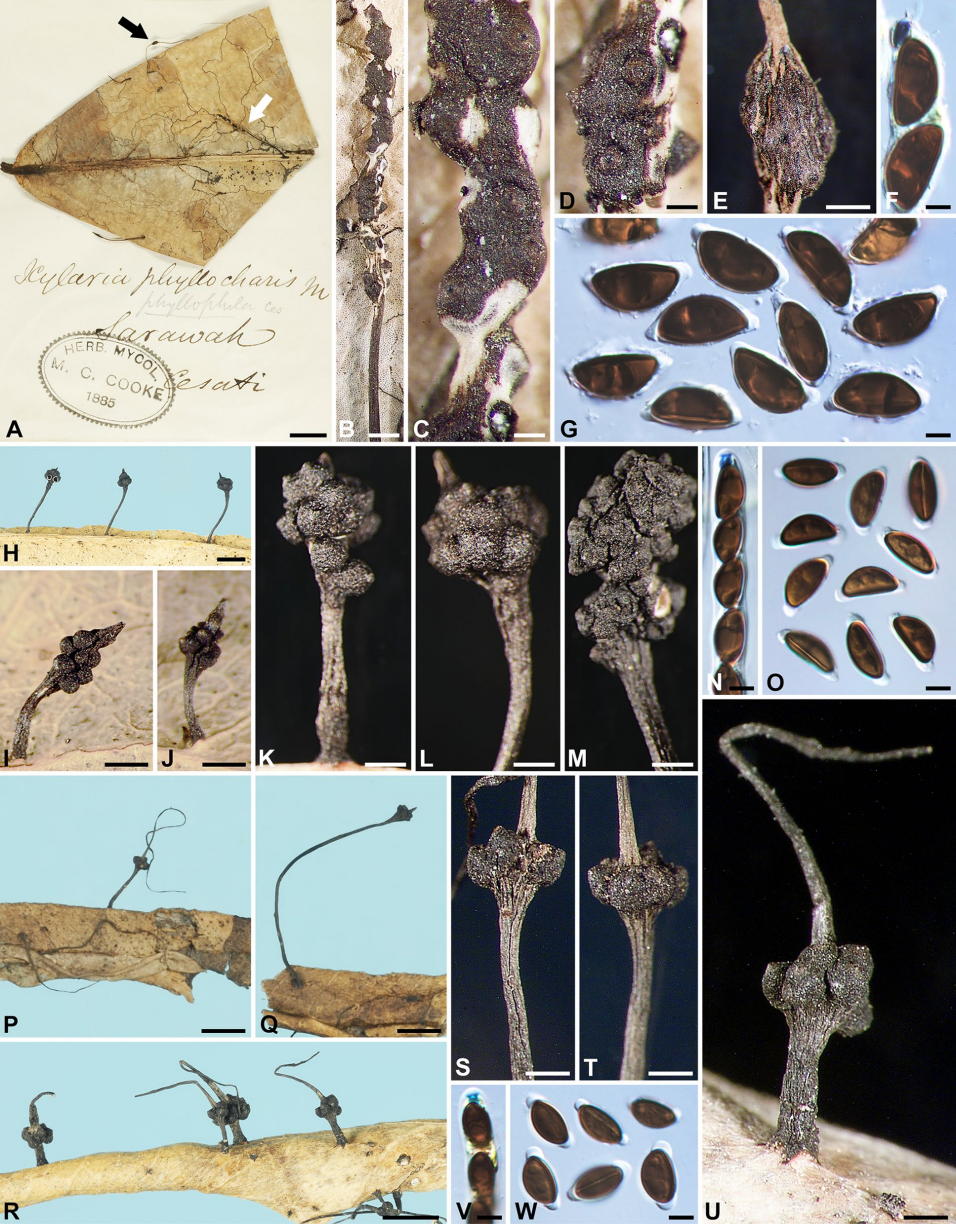
*Xylaria phyllophila*, *X*. *pisoniae*, and *X*. *sicula*. **A**–**G** *X*. *phyllophila* (isotype K[M] 236749). **A**,** B** Stroma, pointed towards by the white arrow in **A** and enlarged in ** B**; the black arrow in A pointing towards the stroma of *X*. *minuscula* that is enlarged in **E**. **C**, **D** Stromatal surfaces. **E** Stroma of *X*. *minuscula* mixed within the isotype specimen. **F** Ascal apical ring and ascospores. **G** Ascospores. **H**–**O** *X*. *pisoniae* (isotype WSP). **H**–**J** Stromata. **K**–**M** Stromatal surfaces. **N** Ascal apical ring and ascospores. **O** Ascospores. **P**–**T** *X*. *sicula* (**P**, **Q**, **S**, **T** BPI 714381 from Algeria; R, U–W K[M] 136350 from Greece). **P**–**R** Stromata. **S**–**U** Stromatal surfaces. **V** Ascal apical ring and ascospores. **W** Ascospores. Bars in **A** = 1 cm; **B**, **I**, **J** = 1 mm; **C**, **D** = 0.25 mm; **E**, **K**–**M**, **S**–**U** = 0.5 mm; **H**, **P**–**R** = 2.5 mm; **F**, **G**, **N**, **O**, **V**, **W** = 5 μm

***Xylaria phyllophila*** Ces., Atti Accad. Sci. Fis. 8: 15. 1879. Fig. 10A–G.

Stroma cylindrical at fertile part, unbranched, with an acute apex, on a glabrous stipe, 22 mm in total length, 9 mm high × 1 mm broad at fertile part; surface blackish brown, with conspicuous perithecial mounds, lacking an outer layer, underlain with a thin, soft layer ca. 10 μm thick or less, concolorous with the surface; interior white, homogeneous, soft. Perithecia spherical, 350–500 μm broad. Ostioles papillate, 50–80 μm broad at base. Asci not intact, with an apical ring staining deep blue in Melzer’s iodine reagent, inverted hat-shaped, 3.5–4 μm high × 3–4 μm broad. Ascospores dark brown, unicellular, ellipsoid-inequilateral, with narrowly rounded ends, smooth, (15–)16.5–18(–19) × (7.5–)8–9(–9.5) µm (17.3 ± 0.9 × 8.7 ± 0.5 μm, N = 40), with a straight germ slit spore-length or nearly so on the ventral side, surrounded with a hyaline sheath swelling at both ends to form papillate non-cellular appendages; epispore smooth.

**Specimen examined** MALAYSIA. Sarawak, on dead leaves, *Beccari, O.*, as *X. phyllocharis* (lectotype [designated here, MycoBank Typification No. 10013067] of *X. phyllophila* K[M] 236749 ex Cooke herb.).

**Notes** *Xylaria phyllophila* can be readily distinguished from species in the *X*. *phyllocharis* group with long cylindrical stromata and conspicuous perithecial mounds by having ascospores longer than 16 μm and enclosed within a hyaline sheath.

Type material of *X*. *phyllophila* was not located at RO. A part of the type material sent to Cooke by Cesati was kept in two packets at K; both were labeled as *X*. *phyllocharis*, reflecting the doubt raised in the protologue that *X*. *phyllophila* could be *X*. *phyllocharis*. There are five stromata attached to the leaf enclosed within K(M) 236749, but only two of them are fertile. One stroma has a long cylindrical fertile part and larger ascospores (15–)16–18.5(–19) × 8–9.5 μm, while the other has an ellipsoid fertile part and smaller ascospores 13.5–15 × 5.5–6.5 μm. Cesati (1879) originally described *X*. *phyllophila* as having a cylindrical fertile part, 0.5 to 1 cm long, umber in color, torulose, apiculate or indeed aristate but did not give the ascospore measurement. Cooke (1883) drew a long cylindrical stroma in accordance with Cesati’s description and gave the ascospore measurement as 20 × 10 μm. Thus, the stroma with a long cylindrical fertile part is lectotypified for *X*. *phyllophila*, and the description herein is based on one stroma only; it should be noted that the stromatal surface has been partially ruined by insects. The other fertile stroma with an ellipsoid fertile part belongs to *X*. *minuscula* (Fig. 10E). The other K packet contains a leaf attached with two stromata with an ellipsoid fertile part. We have not studied the material enclosed in this packet microscopically but suspect that it is *X*. *minuscula*.

***Xylaria pisoniae*** D. Scott, J. D. Rogers & Y.-M. Ju, in Rogers, Scott & Ju, Harvard Pap. Bot. 6: 189. 2001. Fig. 10H–O.

Stromata capitate to conic at fertile part, unbranched or infrequently branched, with an acute apex, on a glabrous stipe, up to 3–15 mm in total length, 1–3 mm high × 1–2 mm broad at fertile part; surface black, with conspicuous perithecial mounds, lacking an outer layer, underlain with a thin, black layer ca. 20 μm thick; interior white, homogeneous, soft. Perithecia spherical, 200–400 μm broad. Ostioles slightly papillate, sometimes encircled with a paler area, ca. 50 μm broad at base. Asci with eight ascospores arranged in uniseriate to partially biseriate manner, cylindrical, 120–155 μm total length, the spore-bearing part 65–95 μm long × 7–9 μm broad, with an apical ring staining blue in Melzer’s iodine reagent, inverted hat-shaped to cylindrical, 2.5–3 μm high × 2–2.5 μm broad. Ascospores brown to dark brown, unicellular, ellipsoid-inequilateral, with narrowly to broadly rounded ends, smooth, (10–)10.5–12(–12.5) × (5–)5.5–6(–6.5) µm (11.3 ± 0.6 × 5.7 ± 0.3 μm, N = 40), with a straight germ slit spore-length or nearly so on the ventral side, surrounded with a hyaline sheath swelling at both ends to form papillate non-cellular appendages; epispore smooth.

Cultures and anamorph were reported by Rogers et al. (2001). The anamorph was found on the stromata produced in culture and collected from the field. The teleomorph was produced on OA in 6 wk.

**Specimen examined** USA. Hawaiian Islands, Hawaii, Volcanoes National Park, Pu’u Puaulu (Bird Park), on dead leaves of *Pisonia brunoniana*, 15 Jan 2000, *Rogers, J. D.* (isotype of *X. pisoniae* WSP).

**Notes** *Xylaria pisoniae* has fairly minute stromata characterized by an acute apex and a capitate or conic fertile part roughened by conspicuous perithecial mounds. It is known only from the type specimen collected from the Hawaiian Islands. *Xylaria nainitalensis* and *X*. *sicula* are morphologically similar to *X*. *pisoniae*, from which they can be readily distinguished by their acicular stromatal apex that is much longer than the fertile part.

***Xylaria polysporicola*** H.-X. Ma & X.-Y. Pan, in X.-Y. Pan, Z.-K. Song, Z. Qu, T.-D. Liu & H.-X. Ma, MycoKeys 86: 57. 2022.

For a description of the teleomorph, see Pan et al. (2022) where illustrations of stromata, asci, and ascospores are also provided. *Xylaria polysporicola* is characterized by the following features: stromata cylindrical at fertile part, unbranched or occasionally branched once at clavae, with an acute apex, on a glabrous stipe, 1–4 cm in total length, 2–15 mm long × 0.5–1.6 mm broad at fertile part; stromatal surface black, slightly polished, with conspicuous perithecial mounds and fine longitudinal striations, lacking an outer layer, underlain with a thin, soft layer ca. 70 μm thick, concolorous with the surface, with a white, soft interior; perithecia subspherical, 450–600 μm diam, with a papillate ostiole ca. 70 μm broad at base; ascospores brown to dark brown, ellipsoid-inequilateral, with broadly to narrowly rounded ends, sometimes with one end slightly pinched, smooth, (11.5–)12.5–14.5(–15) × 5.5–8 μm, with a straight germ slit slightly less than spore-length on the ventral side, surrounded with a hyaline sheath swelling at both ends to form papillate non-cellular appendages.

**Notes** *Xylaria polysporicola* is keyed out in the *X*. *phyllocharis* group herein owing to the cylindrical stromata and undulate perithecial mounds. It resembles *X*. *appendiculata* and *X*. *appendiculatoides* but can be distinguished by having larger perithecia and less conspicuous ostioles, which are papillate and approximately 70 μm broad at base as opposed to coarsely conic-papillate and more than 100 μm broad at base. Nonetheless, the presence of fine longitudinal striations on the slightly polished black stromatal surface suggests that *X*. *polysporicola* may be related to *X*. *filiformis* and *X*. *vagans*, where the perithecial mounds are exposed and ascospores are slightly smaller.

***Xylaria sicula*** Pass. & Beltr., Atti della R. Accademia dei Lincei, Transunti, ser. III, 7: 36. 1882. Fig. 10P–W.

≡ *Thamnomyces siculus* (Pass. & Beltrani) Maire, Mycotheca Boreali-Africana, fasc. 4, no. 100. 1913.

≡ *Podosordaria sicula* (Pass. & Beltrani) P. M. D. Martin, J. S. African Bot. 36: 134. 1970.

= *Xylaria sicula* Pass. & Beltr. f. *major* Ciccarone, Nuovo G. Bot. Ital. 53: 357. 1947.

Stromata capitate, depressed-capitate or obconical at fertile part, unbranched, with a long acicular apex, on a glabrous stipe, up to 3–20 mm in total length, 0.7–1.5 mm high × 1–1.5 mm broad at fertile part; surface black, with conspicuous to half-exposed perithecial mounds, lacking an outer layer, underlain with a thin, black layer ca. 20 μm thick; interior white, homogeneous, soft. Perithecia spherical, 400–550 μm broad. Ostioles slightly papillate, sometimes encircled with a paler area, ca. 60 μm broad at base. Asci with eight ascospores arranged in uniseriate manner, cylindrical, 115–165 μm total length, the spore-bearing part 70–90 μm long × 6.5–8 μm broad, with an apical ring staining blue in Melzer’s iodine reagent, inverted hat-shaped, 2.5–3 μm high × 2.5–3 μm broad. Ascospores brown to dark brown, unicellular, ellipsoid-inequilateral, with narrowly to broadly rounded ends, smooth, (8.5–)9.5–11(–12) × (4–)4.5–6(–6.5) µm (10.3 ± 0.7 × 5.3 ± 0.6 μm, N = 40), with a straight germ slit slightly less than spore-length to nearly spore-length on the ventral side, surrounded with a hyaline sheath swelling at both ends to form papillate non-cellular appendages; epispore smooth.

**Specimens examined** ALGERIA: on dead olive leaves, *Maire, R.*, as *X. sicula* (BPI 714381 ex Lloyd herb. 12667, MPU), immature; on dead olive leaves, Nov 1920, *Maire, R. 7337*, as *X. sicula* (BPI 714379 ex Lloyd herb. 10408), immature; L’Alma, sur les feuilles tombées d’*Olea europaea* L., 19 Oct 1912, *Maire, R.*, *Maire’s Mycotheca Boreali-Africana 100*, as *X. sicula* (S F43691 ex Sydow herb., BPI 586419), immature. GREECE: Corfu, Stefanos, near Agios, on fallen leaves of *Olea europaea*, 12 May 2001, *Spooner, B. M.*, as *X. sicula* (K[M] 136350). SPAIN: Balearic Islands, Menorca, on decaying *Olea* leaf litter and debris, 21 May 2002, *Spooner, B. M.*, as *X. sicula* (K[M] 99656).

**Notes** *Xylaria sicula* is mainly distributed in the Mediterranean region, fruiting on fallen olive leaves. The holotype, collected from Sicily, Italy, could not be located by Graniti (1959) who designated the type of *X*. *sicula* f. *major* from Kenya as the neotype of *X*. *sicula*. For notes on the literature of *X*. *sicula*, see Fournier (2014). *Xylaria sicula* f. *major* mentioned in Hsieh et al. (2010) is not this species but rather *X. aristata* var. *aristata*.

Martin (1970) recombined the epithet with *Podosordaria* but cited a reference by Passerini and Beltrani (1883) instead of the protologue in Passerini and Beltrani (1882), where the basionym was published. According to ICN 41.8 (Shenzhen Code), Martin’s error is to be corrected (Martin 1976) and does not affect the valid publication of *Podosordaria sicula* in 1970.

A collection from BRAZIL [São Leopoldo, on fallen leaves, *Theissen, F. 10*, as *X. aristata* (S F72249 ex Bresadola herb.)], which has ascospores (10.0–)10.4–11.4(–11.7) × (4.5–)4.9–5.5(–5.8) µm and evident perithecial mounds, is similar to *X. sicula*. It remains to be determined if this collection is truly *X*. *sicula*, a host-specific species growing on olive leaves in the Mediterranean region, far away from Brazil.

**Fig. 11:**
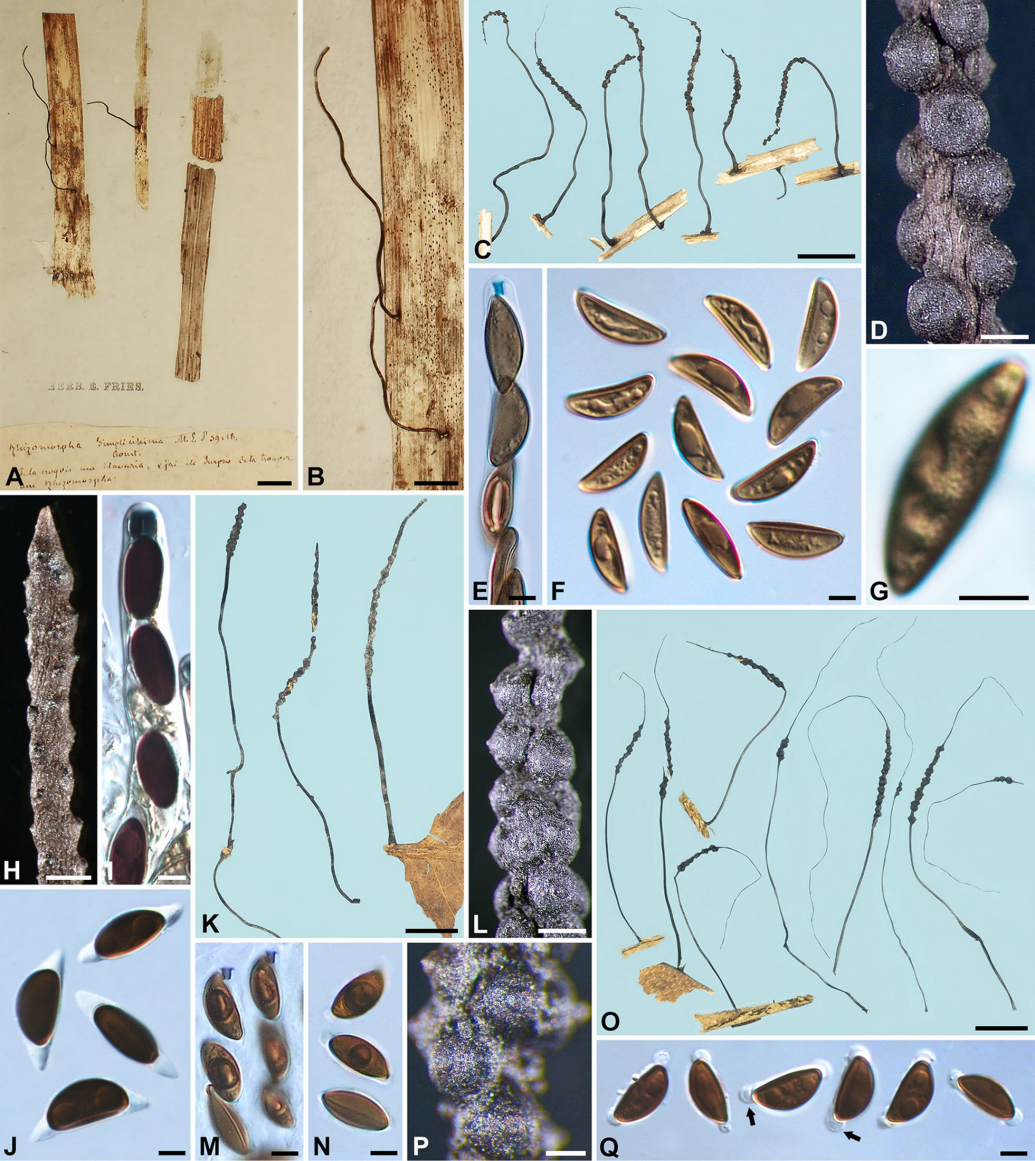
*Xylaria simplicissima*, *X*. sp. AR1741, and *X*. *vagans*. **A**–**G** *X*. *simplicissima* (**A**, **B** lectotype; **C**–**G** holotype of *X. filiformis* var. *caulincola*). **A**–**C** Stromata. **D** Stromatal surface. **E** Ascal apical ring and ascospores. **F**, **G** Ascospores; the ascospore enlarged in G showing the lumpy appearance on the epispore along the germ slit. **H**–**J** *X*. sp. AR1741 (WSP from Venezuela). **H** Stromatal surface showing conic-papillate ostioles that are tilting upwards. **I** Ascal apical ring and ascospores. **J** Ascospores. **K**–**Q** *X*. *vagans* (**K**–**N** holotype; **O**–**Q** WSP Hemmes DEH-1977 from Hawaiian Islands, USA). **K**, **O** Stromata. **L**, **P** Stromatal surfaces. **M** Ascal apical rings and ascospores. **N**, **Q** Ascospores; arrows in **Q** pointing towards two papillate, non-cellular appendages, within which a cellular appendage is visible. Bars in **A**, **C** = 1 cm; **B**, **K**, **O** = 0.5 cm; **D**, **H**, **L** = 0.5 mm; **P** = 0.25 mm; **E**–**G**, **I**, **J**, **M**, **N**, **Q** = 5 μm

**Fig. 12:**
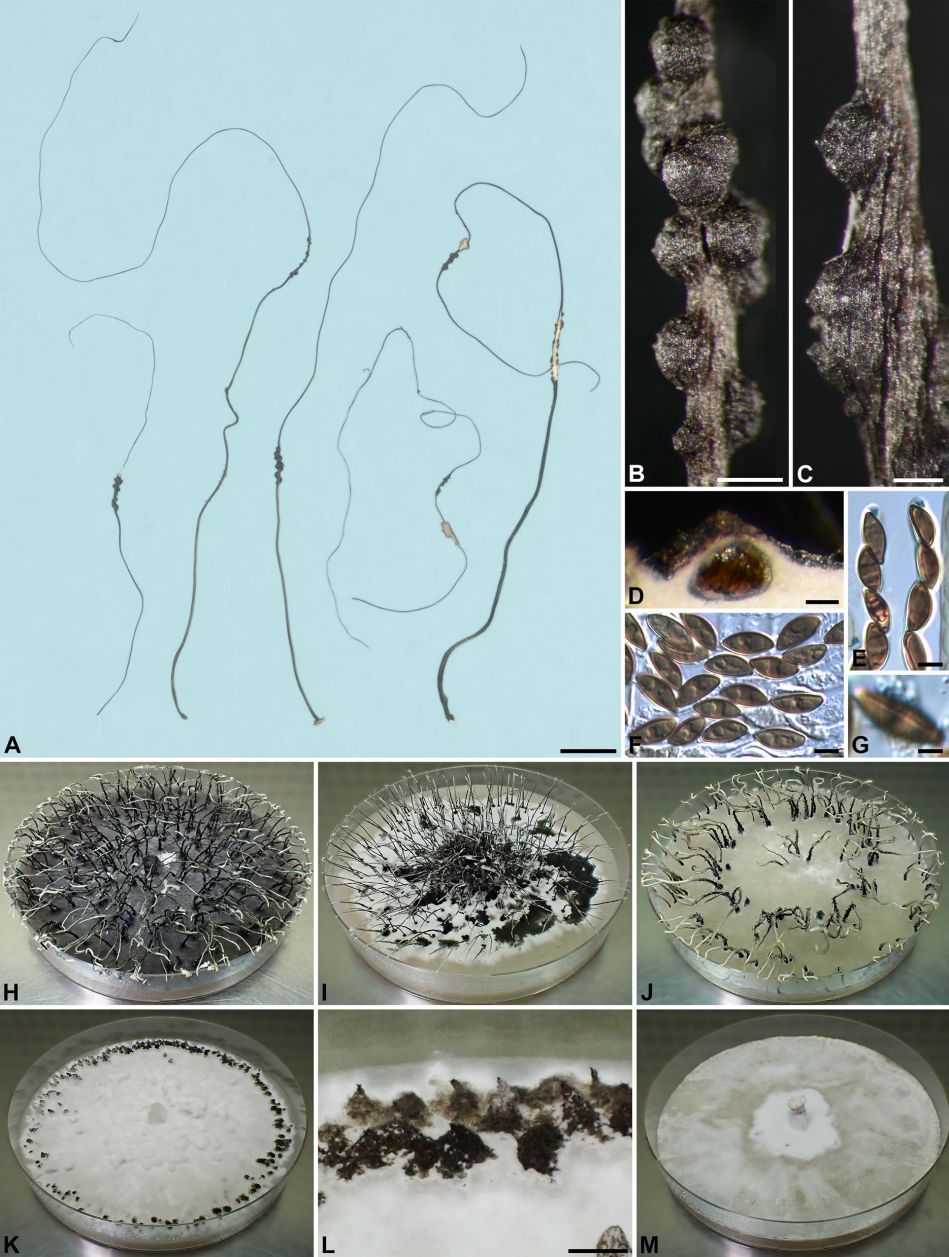
*Xylaria vermiformis* and cultures of various *Xylaria* taxa. **A**–**G***X*. *vermiformis* (holotype). **A** Stromata. **B**, **C** Stromatal surfaces. **D** Vertical section of a depressed-spherical perithecium. **E** Ascal apical rings and ascospores. **F**, **G** Ascospores; the enlarged ascospore in **G** showing a spore-length germ slit. **H**–**M**. Colonies of various *Xylaria* taxa formed on 9-cm Petri plates containing OA. **H** *X*. *amphithele* (from Lechat 5352) at 2 week. **I** *X*. *aristata* var. *aristata* (from Ju 1823) at 4 week. **J** *X*. *simplicissima* (Pennanen MP111004) at 2 week. **K**, **L** *X*. *minuscula* (from holotype) at 4 week; closeup of stunted stromata produced at periphery in **L**. **M** *X*. *vittatipiliformis* (from holotype) at 4 week. Bars in **A** = 5 mm; **L** = 2 mm; **B** = 0.5 mm; **C**, **D** = 0.25 mm; **E**–**G** = 5 μm.

***Xylaria simplicissima*** (Pers.) Y.-M. Ju & H.-M. Hsieh, comb. nov. Figs. 11A–G, 12J.

MycoBank MB848546.

*Basionym: Rhizomorpha simplicissima* Pers., Mycol. Eur. (Erlanga) I, p. 59. 1822.

≡ *Xylaria subularis* Fr., Summa Veg. Scand. II, p. 382. 1849.

**Typification** FRANCE. on herbaceous stems, *Chaillet* (lectotype [designated here, MycoBank Typification No. 10013068] of *Rhizomorpha simplicissima* UPS F-004396 ex Fries herb.), immature. FINLAND. North Karelia, Outokumpu, Rikkaranta, Eskola, N 62.7709, E 28.757, on herbaceous stems, Oct 2011, *Pennanen, M. MP111004* (cultured), comm. *Fournier, J.* (epitype [designated here, MycoBank Typification No. 10013069] of *Rhizomorpha simplicissima* HAST 145982), GenBank: ITS = OQ883722.

= *Sphaeria stipiticola* Swartz, Summa Veg. Scand. (Swartz), p. 55. 1814.

= *Xylaria filiformis* (Albertini & Schwein.) Fr. f. *caulincola* Rehm, Ann. Mycol. 10: 57. 1912.

= *Xylaria jiangsuensis* R. S. Wang & L. Guo, in G. Huang, R. S. Wang, L. Guo & N. Liu, Mycotaxon 130: 302. 2015.

= *Xylaria crinalis* H.-X. Ma, Lar.N. Vassiljeva & Y. Li,, in H.-X. Ma & Y. Li, Sydowia 70: 39. 2018.

Stromata filiform at fertile part, unbranched or rarely branched at clavae, with a long acicular apex, on a glabrous glossy stipe, (10–)25–55(–65) mm in total length, 3–15 mm long × 1–2 mm broad at fertile part; surface blackish, with half-exposed to fully exposed perithecial mounds, lacking an outer layer, underlain with a thin, black layer ca. 20 μm thick; interior white, homogeneous, fragile. Perithecia spherical, 550–750 μm broad. Ostioles slightly papillate, ca. 70 μm broad at base. Asci with eight ascospores arranged in uniseriate manner, cylindrical, 175–220 μm total length, the spore-bearing part 110–130 μm long × 7–8 μm broad, with an apical ring staining blue in Melzer’s iodine reagent, cylindrical to urn-shaped, 3–3.5 μm high × 2.5–3 μm broad. Ascospores brown to dark brown, unicellular, ellipsoid to shortly fusoid, inequilateral, with narrowly rounded ends, smooth, (15–)16.5–19(–21.5) × (5–)5.5–6.5(–7.5) µm (17.7 ± 1.2 × 6.1 ± 0.5 μm, N = 100), with a straight germ slit spore-length on the ventral side, lacking a hyaline sheath; epispore with a lumpy appearance along the germ slit.

Cultures and anamorph. Colonies reaching the edge of 9-cm Petri dish in 2 wk, whitish, mostly submerged, azonate, with diffuse margins. Reverse uncolored. Stromata arising in 2–3 concentric zones, cylindrical, tapering upwards, unbranched, up to 2.6 cm × 0.5–1 mm diam, becoming black from base upwards, white on the surface of upper part and orangish at tip, with abundant clear droplets on the surface. Anamorph not produced.

**Additional specimens examined that are mature** AUSTRIA. Österreich, Steiermark, Schladminger Tauern, Kleinsölktal S von Gröbming, Aufstieg von der Putzentalalm zur Oberen Alm, ca. 1500 m alt., on stems of *Cicerbita alpina*, 18 Sep 1991, *Scheuer, C.*, as *X. filiformis* (WSP 70041 ex GZU). CANADA. Ontario, London, on dead herbaceous stems, Aug–Oct 1911, *Dearness, J.*, *Bartholomew’s Fungi Columbiani 3600*, as *X. filiformis* (HBG, S F131049 ex Sydow herb., WSP 182159); Ontario, London, on old *Archangelica* stems, Jul–Aug 1911, *Dearness, J.*, *Rehm’s Ascomyceten 1969* (holotype of *X. filiformis* var. *caulincola* S F131060 ex Rehm herb., isotypes HBG, S F131063 ex Sydow herb.); Ontario, London, on old stems of *Archangelica* sp. and other plants, 22, 29 Jul 1911, *Dearness, J. 2138*, as *X. filiformis* var. *caulincola* (S F131064 ex Rehm herb.); Ontario, near London, on various dead herbaceous stems, 20 Sep 1911, *Dearness, J.*, as *X. filiformis* var. *caulincola* (HBG); Ontario, pr. London, in caulibus herbarium, praecipue Solidaginis, Sep 1911–1913, *Dearness, J.*, *Sydow’s Fungi Exotici Exsiccati 258*, as *X. filiformis* f. *caulincola* (HBG, L 1038608 ex U 034503, S F131051 ex Sydow herb., S F131053 ex Sydow herb., S F131057 ex Sydow herb.). GERMANY. Oberammergau, Kapellenwand, on stem of *Lunaria rediviva*, Sep 1889, *Allescher*, as *X. filiformis* (S F271420 ex Bresadola herb.). JAPAN. Honshu, Tochigi Pref., Okka-Nikko, Koto-Ku, Bakujo, on *Sasa* sp., 26 Aug 1983, *Samuels, G. J. GS83-342*, as *X.* cf. *filiformis* (WSP ex Rogers herb.). RUSSIA. Berdicino, prov. Jaroslawl, in caulibus emortuis *Epilobii angustifolii* L., Aug 1911, *Serebrianikow*, *Tranzschel & Serebrianikow’s Mycotheca Rossica 328*, as *X. filiformis* (HBG); Russland, St. Petersburger Forst-Institut, in petiolis *Fraxini excelsioris*, Sep 1895, collectors unknown, as *X. filiformis* (S F271421 ex Rehm herb.). USA. New Hampshire, Chocorua, on dead stems and leaves, 4 Aug 1909, *Farlow, W. G.*, *Thaxter’s Reliquiae Farlowianae 97*, as *X. filiformis* (S F131048); North Dakota, Kulm, on herbaceous stems of *Iva xanthiifolia*, 16 Aug 1923, *Brenckle, J. F.*, *Brenckle’s Fungi Dakotenses 575*, as *X. filiformis* f. *caulincola* (WSP 23494).

**Additional specimens examined that are immature** AUSTRIA. Niederösterreich, on petioles of *Rubus* sp., Jun 1905, *Strasser, P.*, as *X. filiformis* (S F271422), immature; Österreich, Steiermark, Rottenmanner Tauern, am Pölsbach S von St. Johann a. Tauern, ca. 1015 m alt., on stems of *Scirpus sylvaticus*, 12 Aug 1984, *Scheuer, C.*, as *X. filiformis* (WSP 70040 ex GZU), immature. CZECH REPUBLIC. Reichenberg in Bohemia, ad petiolos arborum frondosarum, praesertim *Aceris*, *Siegmund Jun., W.*, *Rabenhorst’s Fungi Europaei 917*, as *X. filiformis* (BPI 739338 ex Nitschke herb., HBG [in 2 packets], S F131047 ex Rehm herb.), immature; Reichenberg in Bohemia, on used carpet knitted from plant fibers, *Siegmund Jun., W.*, *Rabenhorst’s Fungi Europaei 57*, as *X. filiformis* (HBG, HBG ex Magnus herb., S F131040 ex Rehm herb., S F131041 ex Sydow herb., S F131046 ex Sydow herb., S F131050 ex Sydow herb.), immature. EUROPE. no detailed locality, on herbaceous stem, *Mougeot, J. B. 989*, as *Rhizomorpha simplicissima* (L 0117822 ex Persoon herb.), immature. FINLAND. Mustiala, På Gråbo, on herbaceous stems, 17 Dec 1865, *Karsten, P. A.*, *Fungi Fennici Exsiccati 559*, as *X. subularis* (K[M]), immature. HUNGARIA. in pratis ”Nagymezö” montium ”Bükk hegyseg”, on stems of *Aconitum variegatum* ssp. *gracile*, Oct 1957, *Tóth, S.*, *Poelt & Scheuer’s Reliquiae Petrakianae 1697*, as *X. filiformis* (S F131039), immature. ITALY. Andalo, on stems of *Senecio cordatus* ssp. *gracile*, Aug 1896, *Bresadola, G.*, as *X. filiformis* (S F131045 ex Bresadola herb.), immature; Bei Paneveggio in Süd-Tyrol, auf faulenden Stengeln von *Aconitum* u. *Cirsium spinossimum*, Aug 1882, *Arnold*, as *X. filiformis* (S F258794 ex Rehm herb.), immature; on leaves, May 1882, *Bresadola, G.*, as *X. filiformis* (S F131052 ex Bresadola herb.), immature. NORWAY. Norvegia, Tr., Lyngen, Övre Karnes, kbl. 1634 III, UTM DC 61 NE, in *Filipenduleto ulmariae*, 3 Aug 1977, *Sivertsen, S.*, as *X. filiformis* (S F131013), immature. SLOVAKIA. Prencow, in foliis emortuis, 28 Oct 1889, *Kmet, A.*, *Fungi Schemnitzienses*, as *X. filiformis* (HBG, S F271423 ex Sydow herb.), immature; Prencow, Mt. Sytno, in caulibus emortius *Ononidis spinosae* L., 27 Jun 1888, *Kmet, A.*, *Fungi Schemnitzienses*, as *X. filiformis* (HBG), immature; Prencow, na háj, in diversis foliis emortuis, 8 Oct 1888, *Kmet, A.*, *Fungi Schemnitzienses*, as *X. filiformis* (S F271424), immature. SWEDEN. Bohuslän: Rödbo parish, Ellesbo, on decaying stems, culms and leaves of various plants under *Scirpus silvaticus* at the edge of a pond, 25 Jul 1942, *Nathorst-Windahl, T.*, *Lundell & Nannfeldt’s Fungi Exsiccati Suecici 1459*, as *X. filiformis* (S F130992), immature; Gästrikland, Gävle, Lövudden, on decaying stems of *Aegopodium podagraria*, 16 Jul 1945, *Nannfeldt, J. A. 7747*, *Lundell & Nannfeldt’s Fungi Exsiccati Suecici 1460*, as *X. filiformis* (S F131023), immature; Jämtland, Åre s:n, Åre, on herbaceous stems of *Aconitum septentrionale*, 22 Aug 1931, *Eliasson, A. G.*, as *X. filiformis* (S F131004), immature; Medelpad, Borgsjö par., Granbodåsen chalet, on dead stalks of *Urtica dioeca* in mixed forest, 11 Sep 1995, *Lundqvist, N. 20263*, as *X. filiformis* (S F130991), immature; on dead stem, *Swartz, O.* (lectotype of *Sphaeria stipiticola* S F131030 ex Swartz herb.), immature; Skåne, on herbaceous stems, collectors unknown, as *Sphaeria filiformis* (UPS F-012070 ex Fries herb.), immature; Stockholm, on herbaceous stems, collectors unknown, as *X. stipiticola* (UPS F-133310 ex Fries herb.), immature; Uppland, Fasterna sn, Grindstugan i Granby, på gamla hallonpinnar, Oct 1988, *Anderberg, A.*, as *X. filiformis* (S F131009), immature; Uppland, Funbo parish, Storudden, on decaying petioles of *Fraxinus excelsior*, 5 Jul 1942, *Åberg, E.*, *Lundell & Nannfeldt’s Fungi Exsiccati Suecici 1461b*, as *X. filiformis* (S F131027), immature; Uppland, Funbo parish, Storudden, on decaying stems of *Urtica dioica*, 5 Jul 1942, *Åberg, E.*, *Lundell & Nannfeldt’s Fungi Exsiccati Suecici 1461a*, as *X. filiformis* (S F131025), immature; Uppland, Lidingö, Hersbyvägen, på c 10 cm bred stambas av *Lonicera tatarica* (rosentry), Apr 1985, *Bremer 2254*, as *X. filiformis* (S F131019), immature; Uppland, Stocksund stn till gamla Stocksundsbron, på multnade stjälkar, 17 Oct 1952, *Berggren, G.*, as *X. filiformis* (S F131018), immature; Uppland, Uppsala par., Uppsala, Hort. Bot., on wood, Sep 1853, *Fries, E.*, as *X. subularis* (UPS F-176043 ex Fries herb.), immature.

**Notes** *Xylaria simplicissima* is distributed in temperate regions of Eurasia and North America, where it is commonly associated with dead herbaceous stems and less commonly with fallen leaves. Most herbarium specimens labeled as *X. filiformis* that were collected from these regions are in fact *X. simplicissima*. *Xylaria filiformis* differs from *X. simplicissima* mainly by having a more delicate stromatal axis and smaller ascospores that possess a hyaline sheath. *Xylaria simplicissima* is represented by numerous specimens, but immature stromata are more commonly encountered.

An authentic specimen labeled as *Rhizomorpha simplicissima* (L 0117822) was located in the Persoon herbarium (L), but it has been relabeled as *Sphaeria filiformis*. This specimen is immature and lacks collecting data. Chaillet collected the original material of *R*. *simplicissima*, as indicated in the protologue (Persoon 1822). A specimen (F-004396) labeled as *R*. *simplicissima* from the Fries herbarium (UPS) was identified as a Chaillet specimen from “Frankrike” (France in Swedish) on a paper slip enclosed within the packet; it is herein designated as the lectotype. However, this specimen is immature, and a fully mature Finnish collection, from which a culture was obtained, is designated as the epitype herein.

The specimens labeled as *S*. *stipiticola* and *X*. *subularis* that we studied are immature. The only specimen labeled as *X*. *subularis* from the Fries herbarium (UPS) was collected in 1853, after the name was published, and contains material of *X*. *simplicissima*. Specimen S F131030 from the Swartz herbarium (S), originally labeled as *S*. *stipiticola* and relabeled as *X*. *filiformis*, contains stromata with developing perithecia and is considered the holotype of *S*. *stipiticola*. The stromatal surface has been ruined by insects at places. A Fries specimen labeled as both *X*. *stipiticola* and *X*. *filiformis* was collected from Stockholm and has a similar insect-gnawing pattern to that of S F131030, of which it may be a duplicate.

*Xylaria simplicissima* has a long, entangling history with *X*. *filiformis*. Fries (1816) tentatively identified a fungus collected from herbaceous stems in Skåne, Sweden as *S*. *filiformis* but was certain that it was the same as *S*. *stipiticola* Swartz (Swartz 1814). Later, Fries (1823) divided *S*. *filiformis* into two varieties based on substrate types: var. *filiformis* grew on fallen leaves and petioles, known to him probably only from Albertini and Schweinitz (1805), while var. *b*, equivalent to *S. stipiticola*, grew on herbaceous stems. It should be noted that *S. stipiticola* is a *nomen nudum*, being published without a description/diagnosis. Var. *b* was later equated by Fries (1828, 1830) to *Rhizomorpha simplicissima* Pers. Fries (1849) eventually recognized var. *b* as a distinct species from *X*. *filiformis* [≡ *S*. *filiformis*] and named it *X*. *subularis* Fr., with *R*. *simplicissima* (as “*simplex* P.”) in synonymy. *Xylaria subularis* is thus a superfluous name of *R*. *simplicissima*.

In North America, *X. simplicissima* was described as *X*. *filiformis* f. *caulincola* (Rehm 1912), which is based on a Canadian collection made by J. Dearness. Lloyd (1918b) identified a Canadian specimen from Dearness as *X. filiformis*, with an ascospore size of 16 × 5 μm; this is likely *X. simplicissima*. The description of *X. filiformis* in Ellis and Everhart (1887) also indicates *X. simplicissima*. The protologues of *X*. *jiangsuensis* and *X*. *crinalis* leave little doubts that they are in synonymy with *X*. *simplicissima*.

***Xylaria*** **sp. AR1741** Fig. 11H–J.

Stroma cylindrical at fertile part, unbranched, with an acute apex, on a glabrous stipe, 15 mm in total length, 7 mm long × 0.5 mm broad at fertile part; surface blackish brown, slightly polished, with slight perithecial mounds and fine longitudinal striations, lacking an outer layer, underlain with a thin, soft layer ca. 20 μm thick, concolorous with the surface; interior white, homogeneous, soft. Perithecia spherical, 250–350 μm broad. Ostioles conic-papillate, tilting upwards, 100–120 μm high × 100–120 μm broad at base. Asci with eight ascospores arranged in uniseriate manner, cylindrical, 90–130 μm total length, the spore-bearing part 85–100 μm long × 9–11 μm broad, with an apical ring staining deep blue in Melzer’s iodine reagent, urn-shaped, 4.5–5.5 μm high × 4–5 μm broad. Ascospores dark brown, unicellular, ellipsoid-inequilateral, with broadly to, less frequently, narrowly rounded ends, smooth, (14–)14.5–16(–16.5) × (6.5–)7–8(–8.5) µm (15.2 ± 0.6 × 7.6 ± 0.6 μm, N = 40), with a straight germ slit spore-length on the ventral side, surrounded with a hyaline sheath swelling at both ends to form cone-shaped non-cellular appendages; epispore smooth.

**Specimen examined** VENEZUELA. Territorio Federal Amazonas, Cerro de la Neblina, San Carlos de Rio Negro, near airport, on IVIC plot, on dead leaves, 5 Jan 1985, *Rossman, A. AR1741-434* (WSP ex Rogers herb.).

**Notes** This small Venezuelan collection represents an undescribed species but contains only a fragmented stroma. It closely resembles *X. appendiculatoides* but has cone-shaped secondary appendages at the ascospore ends. Rogers et al. (1988) suspected that this fungus resembles *X*. *lima*, which differs by having a tomentose stromatal fertile part and smaller ascospores with a cone-shaped secondary appendage at each end.

***Xylaria*** **sp. GS7461A** Fig. 7H, J, N, O.

Stromata depressed-capitate at fertile part, unbranched, with an acute apex, on a glabrous stipe, 18–19.5 mm in total length, 1.2–1.4 mm long × 1.6–2.2 mm broad at fertile part; surface dull grayish brown, with conspicuous to half-exposed perithecial mounds, overlain with a dull grayish brown thin layer cracked reticulately into fine plaques 100–200 μm broad, underlain with a black layer ca. 30 μm thick; interior white, homogeneous, soft. Perithecia spherical, 500–600 μm broad. Ostioles papillate, 160–190 μm broad at base. Asci with eight ascospores arranged in uniseriate manner, cylindrical, 200–240 μm total length, the spore-bearing part 140–180 μm long × 10.5–13.5 μm broad, with an apical ring staining blue in Melzer’s iodine reagent, urn-shaped, 5.5–6.5 μm high × 4–5 μm broad. Ascospores brown, unicellular, ellipsoid-inequilateral, with narrowly rounded ends, smooth, (24.5–)26–29(–31) × (7.5–)8–9(–9.5) µm (27.7 ± 1.5 × 8.7 ± 0.5 μm, N = 40), with a spiral germ slit spore-length or slightly less than spore-length on the ventral side, lacking a hyaline sheath; epispore smooth.

**Specimen examined** VENEZUELA. Edo. Trujillo, Parque Nacional Guaramacal, ca. 10 km SW of Batatal, La Defensa, along Río Saguás, Campamiento Granja Bocono, in forest along trail to water source, alt. 2000 m, on petiole of *Cecropia* (Urticaceae)?, 20, 23 Nov 1990, *Samuels, G. J., Hein, B. & Huhndorf, S. M. GS7461A* (HAST 145983 ex BPI).

**Notes** *Xylaria* sp. GS7461A is characterized by a depressed-capitate stromatal fertile part born on a long glabrous stipe and topped with an acute apex and by a spiral germ slit on the ascospores. Interestingly, *X.* sp. GS7461A grows on the same petiole as *X*. *meliacearum* and *X*. sp. GS7461B (Fig. 7H), and all three species have a spiral germ slit on their ascospores. This may give the false impression that they are different manifestations of the same species. However, they are significantly different by their combinations of stromatal morphology and ascospore size range. The stromatal fertile part and ascospores of *X*. *meliacearum*, *X*. sp. GS7461A, and *X*. sp. GS7461B are torulose/(19–)20–23(–27) × (4.5–)5–6 (–6.5) µm, depressed-capitate/(24.5–)26–29(–31) × (7.5–)8–9(–9.5) µm, and cylindrical/(22.5–)23.5–25(–26) × (6–)6.5–7.5(–8) µm, respectively. *Xylaria* sp. GS7461A, and *X*. sp. GS7461B remain undescribed due to limited materials, with only two and one stromata available for study, respectively.

***Xylaria*** **sp. GS7461B** Fig. 7H, K, P, Q.

Stroma cylindrical at fertile part, unbranched, with a mucronate apex, on a glabrous stipe, 5 mm in total length, 3.4 mm long × 1.6 mm broad at fertile part; surface dull brown, with inconspicuous perithecial mounds, overlain with a dull brown thin layer splitting into vague stripes, underlain with a carbonized layer ca. 70 μm thick; interior whitish, homogeneous, soft. Perithecia spherical, 550–650 μm broad. Ostioles papillate, 120–140 μm broad at base. Asci with eight ascospores arranged in uniseriate manner, cylindrical, 185–230 μm total length, the spore-bearing part 140–175 μm long × 8–10 μm broad, with an apical ring staining blue in Melzer’s iodine reagent, urn-shaped, 4–6 μm high × 3.5–4.5 μm broad. Ascospores brown, unicellular, ellipsoid-inequilateral to crescentic, with narrowly rounded ends sometimes bearing a tiny hyaline cellular appendage on one end, smooth, (22.5–)23.5–25(–26) × (6–)6.5–7.5(–8) µm (24.2 ± 0.8 × 7.1 ± 0.5 μm, N = 40), with a spiral germ slit spore-length or slightly less than spore-length on the ventral side, lacking a hyaline sheath; epispore smooth.

**Specimen examined** VENEZUELA. Edo. Trujillo, Parque Nacional Guaramacal, ca. 10 km SW of Batatal, La Defensa, along Río Saguás, Campamiento Granja Bocono, in forest along trail to water source, alt. 2000 m, on petiole of *Cecropia* (Urticaceae)?, 20, 23 Nov 1990, *Samuels, G. J., Hein, B. & Huhndorf, S. M. GS7461B* (HAST 145984 ex BPI).

**Notes** *Xylaria* sp. GS7461B is reminiscent of species in the *X*. *arbuscula* group, where the point-topped stromata are cylindrical and overlain with an outer layer that splits into stripes, but can be separated from the species in the group by its larger ascospores with a spiral germ slit. It grows on a petiole where *X*. *meliacearum* and *X*. sp. GS7461A are also present (Fig. 7H). Also see the notes on *X*. sp. GS7461A.

***Xylaria spiculaticlavata*** Y.-M. Ju & H.-M. Hsieh, sp. nov. Fig. 9O, U–Y.

MycoBank MB848547.

**Typification** FRENCH GUIANA. on a fallen leaf, *Leprieur, F. R. 1208?* (holotype of *X*. *spiculaticlavata* PC 0086071 ex Leprieur herb.), mixed with *X*. *phyllocharis* and *X*. *aristata*.

**Etymology** Referring to the cylindric-clavate stromatal fertile part studded with conical ostioles, resembling a medieval spiked battle club.

Stroma cylindric-clavate at fertile part, unbranched, with a blunt apex, on a glabrous stipe, 76.6 mm in total length, 10.8 mm long × 2.3 mm broad at fertile part; surface brown, lacking perithecial mounds, lacking an outer layer, underlain with a thin, soft layer ca. 30 μm thick, concolorous with the surface; interior white, homogeneous, soft. Perithecia spherical, 600–800 μm broad. Ostioles coarsely conical, slightly tilting upwards, 240–270 μm high × 270–310 μm broad at base. Asci not intact, with an apical ring staining deep blue in Melzer’s iodine reagent, cylindrical, 7.5–8 μm high × 4–4.5 μm broad. Ascospores brown to dark brown, unicellular, ellipsoid-inequilateral, with broadly to narrowly rounded ends, sometimes with one end slightly pinched, smooth, (22–)23.5–27(–28) × (8.5–)9–10.5(–11) µm (25.1 ± 1.8 × 9.7 ± 0.7 μm, N = 10), with a straight germ slit spore-length or nearly so on the ventral side, surrounded with a hyaline sheath swelling at both ends to form papillate non-cellular appendages; epispore smooth.

**Notes** *Xylaria spiculaticlavata* is peculiar among foliicolous *Xylaria* species in having a plane stromatal fertile part studded with conspicuous conical ostioles, perithecia broader than 600 μm, and ascospores longer than 20 μm and broader than 9 μm. Material of *X*. *spiculaticlavata* is mixed in the isotype packet of *X. phyllocharis* from the Leprieur collection (PC), where materials of *X*. *phyllocharis* and *X*. *aristata* are also enclosed. The materials of these three species are separately glued on three paper cards as shown in Fig. 9O, where the stroma of *X*. *spiculaticlavata* is on the right-hand side bearing the pencil writing “Herb. Leprieur 1208?”. Although the material is represented by one stroma only, we formally describe it as new because it possesses a suite of highly distinctive features among the foliicolous *Xylaria* species and appears to be an extremely rare species having not been recollected since Leprieur made the collection during 1835–1849 (Montagne 1855).

***Xylaria vagans*** Petch, Ann. Roy. Bot. Gard. (Peradeniya) 6: 68. 1915. Fig. 11K–Q.

= *Xylaria hedyosmicola* H.-X. Ma & X.-Y. Pan, in X.-Y. Pan, Z.-K. Song, Z. Qu, T.-D. Liu & H.-X. Ma, MycoKeys 86: 52. 2022.

Stromata filiform at fertile part, unbranched, with a long acicular apex, on a glabrous stipe, 20–53 mm in total length, 4–11.5 mm long × 0.7–1.1 mm broad at fertile part; surface black, slightly polished, with half-exposed perithecial mounds and fine longitudinal striations, lacking an outer layer, underlain with a thin, soft layer ca. 20 μm thick, concolorous with the surface; interior white, homogeneous, soft. Perithecia spherical, 300–550 μm broad. Ostioles conic-papillate, 90–120 μm high × 120–160 μm broad at base. Asci with eight ascospores arranged in uniseriate manner, cylindrical, 125–150 μm total length, the spore-bearing part 75–90 μm long × 6.5–8 μm broad, with an apical ring staining blue in Melzer’s iodine reagent, inverted hat-shaped, 2.5–3 μm high × 2–2.5 μm broad. Ascospores brown to dark brown, unicellular, ellipsoid-inequilateral, with narrowly rounded ends sometimes bearing a tiny hyaline cellular appendage on one end, smooth, (11–)11.5–13(–13.5) × (4.5–)5–6(–6.5) µm (12.1 ± 0.7 × 5.5 ± 0.4 μm, N = 80), with a straight germ slit spore-length on the ventral side, surrounded with a hyaline sheath swelling at both ends to form papillate non-cellular appendages; epispore smooth.

**Specimens examined** SRI LANKA. Central Province, Nuwara Eliya District, Hakgala, elev. 5400 ft, on fallen leaves, Jan 1914, *Petch, T. 4093* (holotype of *X*. *vagans* K[M] 144168, isotype BPI 713891 ex Lloyd herb. 10383). USA. Hawaiian Islands, Hawaii, Hilo, on dead leaves of *Melonchia umbellata*, 26 Aug 2000, *Hemmes, D. DEH-1977* (WSP ex Rogers herb.); Hawaiian Islands, Hawaii, Leilani Estates, on dead leaves of mango and *Melastoma* sp., Apr 1996, *Hemmes, D. DEH-1091* (WSP ex Rogers herb.); Hawaiian Islands, Hawaii, Leilaris Estates, on dead leaves of *Tibouchina semidecandra* or soil, 2 Jan 1996, *Hemmes, D. DEH-1052* (WSP ex Rogers herb.).

**Notes** *Xylaria vegans* is quite similar to *X. filiformis* in stromatal morphology and ascospore size range. *Xylaria filiformis* appears to be a temperate counterpart of *X*. *vegans*, from which it differs by having light brown, short fusoid-inequilateral ascospores with narrowly rounded ends frequently pinched on one or both ends.

A specimen [SRI LANKA. Central Province, Nuwara Eliya District, Hakgala, elev. 5400 ft, on fallen leaves, *Petch, T. 4094*, as *X*. *vagans* (BPI 713889 ex Lloyd herb. 10386)] that Lloyd received from Petch contains rhizomorphs. However, Petch (1915), while discussing horse-hair blights, mentioned that *X*. *vagans* was common in Hakgala on dead leaves but “not necessarily in direct connection with the black rhizomorphs.” An Indian specimen [INDIA. on fallen leaves, Feb 1980, *Narula, A. M. H-13* (WSP ex Rogers herb.)] labeled as *X*. *filiformis* is similar to *X*. *vagans* but has slightly larger ascospores (14–)14.5–15.5(–16.5) × (5.5–)6–6.5(–7) µm (14.9 ± 0.6 × 6.2 ± 0.4 μm, N = 20).

*Xylaria vagans* was included in Hsieh et al. (2010) as *X*. sp. 6 from Hawaiian Islands, which was suspected by Rogers and Ju (2012) to be an undescribed species. *Xylaria hedyosmicola* described by Pan et al. (2022) possesses features of *X*. *vagans*, of which we consider a synonym. The synonymy is further corroborated by the high sequence identities at three loci between *X*. sp. 6 (DEH-1052) and *X*. *hedyosmicola* (FCATAS856) from Hainan, China, shown by BLAST searches: 98.99% (GQ478217 vs. MZ221183) at β-tubulin, 99.92% (GQ844795 vs. MZ683407) at RPB2, and 98.66% (GU300082 vs. MZ227121) at ITS.

***Xylaria vermiformis*** Y.-M. Ju, H.-M. Hsieh, sp. nov. Fig. 12A–G.

MycoBank MB848548.

**Typification** INDIA. Simla, on way to Taradevi Temple, on fallen angiospermous leaves, 9 Sep 1982, *Singh, M. 381*, as *X.* cf. *sicula* (holotype of *X. vermiformis* WSP ex Rogers herb.).

**Etymology** Referring to the wavy, worm-shaped stromata.

Stromata filiform at fertile part, unbranched, with a long acicular apex, on a glabrous stipe, 35–83 mm in total length, 2.4–6.3 mm long × 0.5–0.9 mm broad at fertile part; surface black, with half-exposed perithecial mounds, lacking an outer layer, underlain with a thin, soft layer ca. 20 μm thick, concolorous with the surface; interior white, homogeneous, soft. Perithecia depressed-spherical, 250–400 μm broad × 150–250 μm high. Ostioles conic-papillate, 80–120 μm high × 110–140 μm broad at base. Asci with eight ascospores arranged in uniseriate manner, cylindrical, 95–125 μm total length, the spore-bearing part 55–70 μm long × 5.5–6.5 μm broad, with an apical ring staining blue in Melzer’s iodine reagent, inverted hat-shaped, 1.5–2 μm high × 1.5–2 μm broad. Ascospores brown to dark brown, unicellular, ellipsoid to shortly fusoid, inequilateral, with narrowly rounded ends, smooth, (9–)9.5–10.5(–11) × (3.5–)4–4.5(–5) µm (10.1 ± 0.5 × 4.4 ± 0.3 μm, N = 40), with a straight germ slit spore-length or nearly so on the ventral side, lacking a hyaline sheath; epispore smooth.

**Notes** *Xylaria vermiformis* has half-exposed perithecial mounds arranged on the filiform stromata that terminate into an acicular stromatal apex and thus in general resembles *X*. *filiformis*, *X*. *simplicissima*, and *X*. *vagans*. *Xylaria filiformis* and *X*. *vagans* can be readily separated from *X*. *vermiformis* by having a hyaline sheath swelling at both ends to form papillate non-cellular appendages, and *X*. *simplicissima* can be separated mainly by having larger ascospores.

**Fig. 13:**
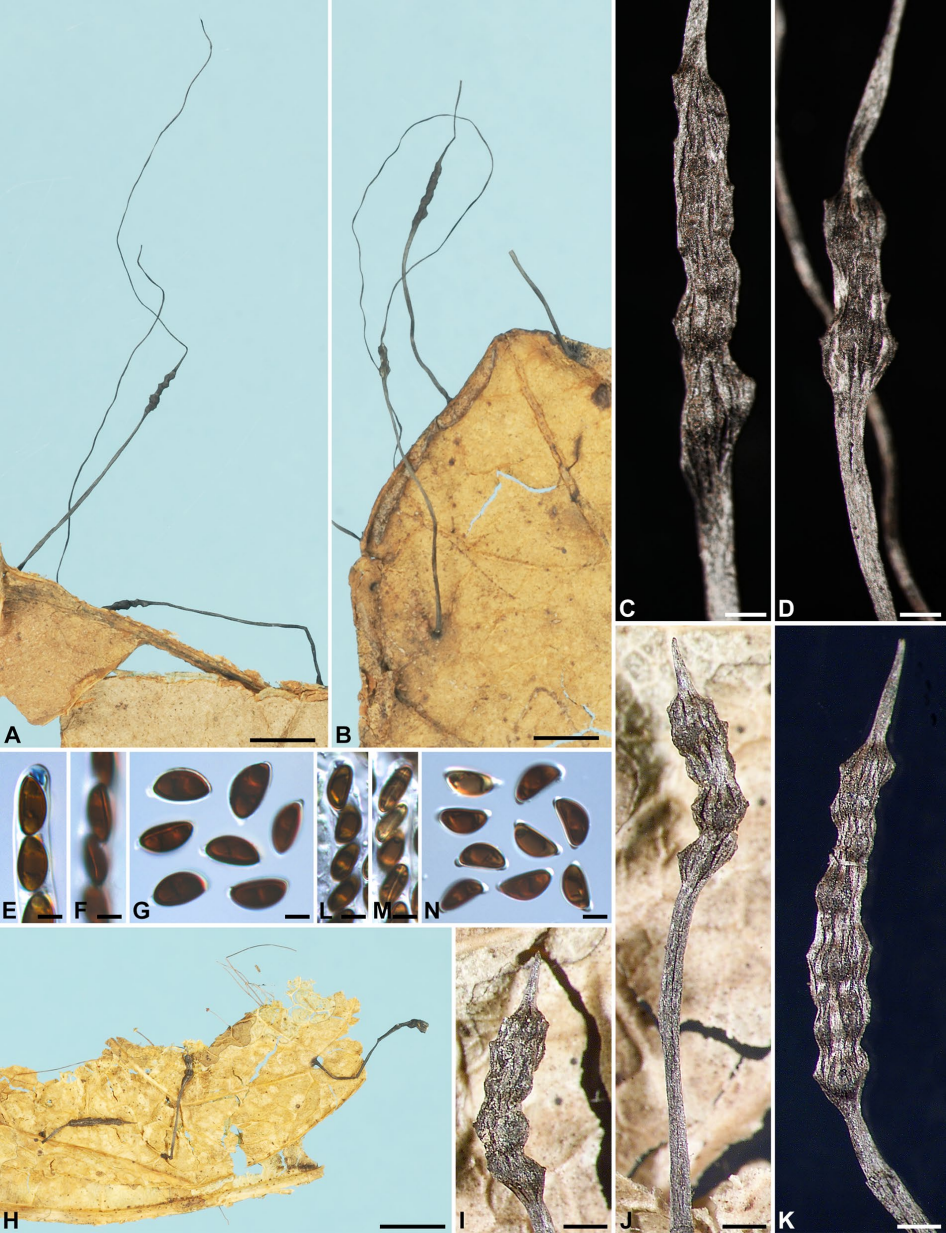
*Xylaria vittatipiliformis* and *X*. *vittiformis*. **A**–**G** *X*. *vittatipiliformis* (holotype). **A**,** B** Stromata. **C**, **D** Stromatal surfaces. **E** Ascal apical ring and ascospores. **F**–**G** Ascospores. **H**–**N** *X*. *vittiformis* (holotype). **H** Stromata. **I**–**K** Stromatal surfaces. **L** Ascal apical ring and ascospores. **M**,** N** Ascospores. Bars in **A**, **B**, **H** = 5 mm; **C**, **D**, **I**–**K** = 0.5 mm; **E**–**G**, **L**–**N** = 5 μm.

***Xylaria vittatipiliformis*** Y.-M. Ju, H.-M. Hsieh & Fournier, sp. nov. Figs. 12M, 13A–G.

MycoBank MB848549.

**Typification** FRENCH WEST INDIES. Guadeloupe, Basse-Terre, Petit-Bourg, trail to La Mamelle, hygrophilic rainforest, on a dead leave in the litter, 14 Aug 2010, *Lechat, C. CLLGUAD029* (cultured) (holotype of *X. vittatipiliformis* LIP, isotype HAST 145985), GenBank: ITS = OQ883723.

**Etymology** Referring to the stromata overlain with an outer layer that splits into band-like stripes and terminates into a long hair-like apex.

Stromata cylindrical at fertile part, unbranched, with a long acicular apex, on a glabrous stipe, 61–78 mm in total length, 3–6 mm long × 0.8–1 mm broad at fertile part; surface black, with inconspicuous to conspicuous perithecial mounds, overlain with a grayish brown outer peeling layer split into band-like stripes, underlain with a black layer 40–50 μm thick; interior white, homogeneous, soft. Perithecia spherical, 400–500 μm broad. Ostioles slightly papillate, ca. 70 μm high × 100–150 μm broad at base. Asci with eight ascospores arranged in uniseriate manner, cylindrical, 110–160 μm total length, the spore-bearing part 70–85 μm long × 7–8.5 μm broad, with an apical ring staining blue in Melzer’s iodine reagent, inverted hat-shaped, 2.5 μm high × 2–2.5 μm broad. Ascospores brown to dark brown, unicellular, ellipsoid-inequilateral, with narrowly to broadly rounded ends frequently bearing a tiny cellular appendage on one end, smooth, (10–)11–12(–12.5) × (5.5–)6–7(–7.5) µm (11.4 ± 0.6 × 6.5 ± 0.5 μm, N = 40), with a straight germ slit spore-length or nearly so on the ventral side, lacking a hyaline sheath; epispore smooth.

Cultures and anamorph. Colonies reaching the edge of 9-cm Petri dish in 3 wk, white, velvety, appressed, azonate, with diffuse margins. Reverse uncolored. Stromata and anamorph not produced.

**Notes** *Xylaria vittatipiliformis* is characterized by cylindrical stromata topped with a long acicular apex and overlain with an outer peeling layer splitting into band-like stripes. Its stromatal surface is much like that of *X*. *vittiformis*, from which *X*. *vittatipiliformis* can be readily separated by having larger ascospores that lack a surrounding hyaline sheath. The type material of *X*. *vittatipiliformis* was described and illustration as *Xylaria* sp. CLLGUAD 029 by Fournier et al. (2020).

***Xylaria vittiformis*** Y.-M. Ju & H.-M. Hsieh, sp. nov. Fig. 13H–N.

MycoBank MB848550.

**Typification** BRAZIL. Rio Grande do Sul, São Leopoldo, in foliis aridis, 1907, *Theissen, F.*, *Rick’s Fungi Austro-Americani 353*, as *X. phyllocharis* Mont. (holotype of *X. vittiformis* HBG, isotype HBG ex Magnus herb.).

**Etymology** Referring to the band-shaped outer peeling layer on the stromatal surface.

Stromata cylindrical to cylindric-fusoid at fertile part, unbranched, with an acuminate apex, on a glabrous stipe, 7.7–14 cm in total length, 2.3–4.1 cm long × 0.6–0.9 mm broad at fertile part; surface black, with inconspicuous to conspicuous perithecial mounds, overlain with a grayish brown outer peeling layer split into band-like stripes, underlain with a thin, black layer ca. 10 μm thick; interior white, homogeneous, soft. Perithecia spherical, 150–200 μm broad. Ostioles slightly papillate, ca. 30 μm high × 30–50 μm broad at base. Asci with eight ascospores arranged in uniseriate manner, cylindrical, immeasurable due to lacking intact stipes, the spore-bearing part 55–65 μm long × 6–7 μm broad, with an apical ring staining blue in Melzer’s iodine reagent, inverted hat-shaped, 1.5 μm high × 2–2.5 μm broad. Ascospores brown to dark brown, unicellular, ellipsoid-inequilateral, with narrowly rounded ends, smooth, (8–)8.5–10(–11) × (4–)4.5–5(–5.5) µm (9.3 ± 0.6 × 4.8 ± 0.3 μm, N = 40), with a straight germ slit spore-length on the ventral side, surrounded with a hyaline sheath swelling at both ends to form papillate non-cellular appendages; epispore smooth.

**Additional specimen examined** BRAZIL. Rio Grande do Sul, São Leopoldo, on fallen leaves, *Theissen, F.*, *Theissen’s Decades Fungorum Brasiliensium 105*, as *X. phyllocharis* (HBG).

**Notes** *Xylaria vittiformis* resembles *X*. *vittatipiliformis* in having the outer peeling layer of stromata splitting into band-like stripes but differs from the latter by having smaller ascospores enclosed within a hyaline sheath which forms a non-cellular appendage at each end and the sterile stromatal apex not being acicular or long hair-like. Known material of *X*. *vittiformis* was collected by F. Theissen from Brazil and distributed among the exsiccata *Fungi Austro-Americani 353* by J. Rick and *Decades Fungorum Brasiliensium 105* by F. Theissen as *X*. *phyllocharis*. *Xylaria phyllocharis* lacks an outer peeling layer on stromata and has a vinaceous brown tinge on the stromatal surface.

### Identification key to accepted taxa

Species that have a newly proposed name herein or that are likely undescribed are in boldface. This key is constructed mainly for identification purpose and is not intended to suggest the phylogenetic relationships between the treated species.

1. Perithecia contained in capitate or obconical head or aggregated into clumps along stromata...3 (*X*. *heloidea* group).
2. Perithecia distributed along stromata, not aggregated into clumps...2.
3. Stromata filiform; perithecial mounds half-exposed to fully exposed, discrete or loosely packed... 18 (*X*. *filiformis* group).
4. Stromata cylindrical; perithecial mounds inconspicuous to conspicuous or half-exposed in rare cases, often closely packed...27 (*X*. *phyllocharis*).
5. Ascospores longer than 20 μm long...4.
6. Ascospores shorter than 20 μm long...5.
7. Ascospores (21.5–)22.5–24.5(–26) × (6.5–)7–8(–9) µm, with a straight germ slit, surrounded with a hyaline sheath forming a long, tubular non-cellular appendage at each end...*X. axifera*.
8. Ascospores (24.5–)26–29(–31) × (7.5–)8–9(–9.5) µm, with a spiral germ slit, lacking a hyaline sheath...***X.*** **sp. Samuels GS7461A**.
9. Stromatal fertile parts with plane to inconspicuous perithecial mounds...6.
10. Stromatal fertile parts with conspicuous to half-exposed perithecial mounds...13.
11. Ascospores 8–9(–9.5) × 4–4.5(–6.6) µm....***X.*** ***imminuta***.
12. Ascospores longer than 10 μm...7.
13. Perithecia distributed on the top of stromatal fertile parts only...8.
14. Perithecia distributed on the entire stromatal fertile parts...9.
15. Stromatal fertile parts capitate; ascospores (12.5–)13–15(–16) × (7.5–)8–9(–10) µm...***X. clusiae***.
16. Stromatal fertile parts peltate; ascospores 12–14 × 6–8 μm ...*X. memecyli*.
17. Stromatal fertile parts globose; ascospores (13–)13.5–15(–16.5) × (6–)6.5–7.5(–8) µm, surrounded with a hyaline sheath swelling at both ends to form non-cellular appendages...*X*. *hypsipoda*.
18. Ascospores lacking a hyaline sheath...10.
19. Ascospores (15–)15.5–17(–18) × (6.5–)7.5–9(–9.5) µm...***X. hispidipes***.
20. Ascospores smaller...11.
21. Ascospores dark brown, nearly semicircular to broadly ellipsoid-inequilateral, with broadly rounded ends to, less frequently, narrowly rounded ends, (10–)10.5–12.5(–14) × (5.5–)6–7(–7.5) µm...*X. delicatula*.
22. Ascospores brown to dark brown, ellipsoid-inequilateral, with narrowly rounded ends...12.
23. Ascospores (10–)10.5–12.5(–14) × (5.5–)6–7(–7.5) µm...*X*. *aristata* var. *aristata*.
24. Ascospores (13.5–)14–16(–17) × (5.5–)6–7(–7.5) µm...*X*. *aristata* var. *hirsuta*.
25. Ascospores (14.5–)15.5–18(–19) × (5–)5.5–6.5(–7) µm...*X*. *heloidea*.
26. Ascospores shorter than 15 μm...14.
27. Stromata with a long acicular apex, much longer than the fertile part...15.
28. Stromata with an acute apex, shorter than the fertile part...16.
29. Ascospores (10.5–)11.5–13.5(–15) × (5–)5.5–6.5(–7.5) µm...*X. nainitalensis*.
30. Ascospores (8.5–)9.5–11(–12) × (4–)4.5–6(–6.5) µm...*X*. *sicula*.
31. Ascospores (12–)12.5–15.5(–17) × (5–)6–7.5(–8) µm...*X*. *amphithele*.
32. Ascospores shorter...17.
33. Ascospores (10–)10.5–12(–12.5) × (5–)5.5–6(–6.5) µm...*X*. *pisoniae*.
34. Ascospores (7.5–)8.5–9.5(–10) × (3.5–)4–4.5(–5) µm...*X*. *petchii*.
35. Ascospores encircled with a hyaline sheath...19.
36. Ascospores lacking a hyaline sheath...21.
37. Ascospores brown to dark brown, 12–13.5 × 4–5 μm, with the hyaline sheath not swelling at ends... *X. eugeniae*.
38. Ascospores with the hyaline sheath swelling at both ends to form non-cellular appendages...20.
39. Ascospores short fusoid-inequilateral, light brown, (9.5–)11.5–13.5(–14.5) × (4–)4.5–5.5(–6) µm; mainly distributed in northern temperate regions...*X*. *filiformis*.
40. Ascospores ellipsoid-inequilateral, brown to dark brown, (11–)11.5–13(–13.5) × (4.5–)5–6(–6.5) µm; mainly distributed in the tropics and subtropics...*X*. *vagans*.
41. Stromata hairy on the entire surface; ascospores (11.5–)12–14(–15) × (4.5–)5 μm...*X. castilloi*.
42. Stromata glabrous on the fertile part...22.
43. Ascospores mostly longer than 16 μm...23.
44. Ascospores mostly shorter than 16 μm...24.
45. Ascospore germ slit straight, (15–)16.5–19(–21.5) × (5–)5.5–6.5(–7.5) µm...***X***. ***simplicissima***.
46. Ascospore germ slit spiral, (19–)21.5–27.5(–31.5) × (5–)5.5–7(–8) µm...*X*. *meliacearum*.
47. Ascospores longer than 11 μm long Ascospores 12–14.5(–16) × 4–4.5(–5) µm...*X. duranii*.
48. Ascospores shorter than 11 μm long...25.
49. Ascospores (5.5–)6–7 × 3–3.5(–4) µm...*X. diminuta*.
50. Ascospores larger...26.
51. Stromata slender, with a long acicular apex; ascospores (9–)9.5–10.5(–11) × (3.5–)4–4.5(–5) µm...***X***. ***vermiformis***.
52. Stromata more slender, with an acuminate or mucronate apex; ascospores (9–)9.5–10.5(–11) × (5.5–)6–6.5(–7) µm...***X***. ***noduliformis***.
53. Stromatal fertile parts overlain with spikes or a tomentum...28.
54. Stromatal fertile parts not overlain with spikes or a tomentum...31.
55. Stromata overlain with stiff spikes on the surface; ascospores (10.5–)11–12.5(–14.5) × (7–)7.5–8.5(–9) µm, lacking a hyaline sheath...*X*. *asperata*.
56. Stromata overlain with a tomentum... 29.
57. Mature stromata overlain with a striped peeling layer; ascospores (10–)10.5–11.5(–12) × (5.5–)6–6.5(–7) µm, lacking a hyaline sheath...*X. maitlandii*.
58. Mature stromata not overlain with an outer layer; ascospores surrounded a hyaline sheath swelling at both ends to form non-cellular appendages...30.
59. Ascospores (14.5–)15–16.5(–17) × (8.0–)8.5–9.5(–10) µm...***X. allima***.
60. Ascospores (10–)10.5–12(–14) × (5–)6–7(–7.5) µm...*X*. *lima*.
61. Ascospores (22.5–)23.5–25(–26) × (6–)6.5–7.5(–8) µm, with a sigmoid to spiral germ slit...***X.*** **sp. Samuels GS7461B**.
62. Ascospores with a straight to slightly oblique germ slit...32.
63. Ascospores longer than 16 μm long...33.
64. Ascospores shorter than 16 μm long...35.
65. Ascospores (15–)16.5–18(–19) × (7.5–)8–9(–9.5) µm...*X*. *phyllophila*.
66. Ascospores longer than 20 μm...34.
67. Perithecial mounds conspicuous; ascospores light brown to brown, (20.5–)21.5–23.5(–24.5) × (6–)6.5–8(–9) µm...*X. luxurians*.
68. Perithecial mounds plane to inconspicuous; ascospores (22–)23.5–27(–28) × (8.5–)9–10.5(–11) µm...***X***. ***spiculaticlavata***.
69. Ascospores 8–10 × 4–6 μm, with a much less than spore-length germ slit ...*X. kamatii*.
70. Ascospores with a spore-length germ slit or nearly so...36.
71. Mature stromata overlain with a longitudinally split peeling layer...37.
72. Mature stromata lacking an outer layer...40.
73. Stromatal outer peeling layer split into band-like stripes...38.
74. Stromatal outer peeling layer split into narrow or thread-like stripes...39.
75. Stromata terminating into an acuminate sterile apex; ascospores (8–)8.5–10(–11) × (4–)4.5–5 (–5.5) µm, surrounded with a hyaline sheath swelling at both ends to form non-cellular appendages...***X***. ***vittiformis***.
76. Stromata terminating into a long acicular apex; ascospores (10–)11–12(–12.5) × (5.5–)6–7(–7.5) µm, lacking a hyaline sheath, commonly bearing a cellular appendage...***X***. ***vittatipiliformis***.
77. Ascospores (8.5–)9–11 × 4–6 μm ...*X. foliicola*.
78. Ascospores (13.5–)14–15(–17) × (4.5–)5–6(–7) µm...***X***. ***minuscula***.
79. Ascospores lacking a hyaline sheath...41.
80. Ascospores surrounded with a hyaline sheath swelling at both ends...42.
81. Stromatal surface slightly undulate; ascospores (9–)10–11.5(–12.5) × (4.5–)5.5–6.5(–7) µm...*X*. *phyllocharis*.
82. Stromatal surface tuberculate; ascospores (9–)9.5–10.5(–11.5) × (5–)5.5–6(–6.5) µm...***X***. ***neblinensis***.
83. Ascospores (14–)14.5–16(–16.5) × (6.5–)7–8(–8.5) µm, with cone-shaped non-cellular appendages...***X***. **sp**. **AR1741**.
84. Ascospores with papillate non-cellular appendages...43.
85. Stromatal surface black; ostioles papillate, not tilting upwards; ascospores (11.5–)12.5–14.5(–15) × 5.5–8 μm...*X*. *polysporicola*.
86. Stromatal surface brown, dark brown to blackish brown; ostioles coarsely conic-papillate, tilting upwards...44.
87. Stromatal surface tinged vinaceous, with slight perithecial mounds; ascospores (11.5–)12.5–14(–15) × (6–)6.5–7.5(–9) µm...*X*. *appendiculata*.
88. Stromatal surface not tinged vinaceous, with conspicuous perithecial mounds; ascospores (14–)15–16(–17) × (6.5–)7.0–7.5(–8) µm...***X. appendiculatoides***.

## Discussion

The diversity of leaf- and petiole-inhabiting *Xylaria* species is mainly found in tropical and subtropical regions except for *X*. *filiformis* and *X*. *simplicissima*, which are prevalent in the northern temperate regions. Host specificity is unclear for most of these species due to the scarcity of available collections. *Xylaria sicula* specifically grows on fallen olive leaves primarily in the Mediterranean region. Læssøe and Lodge (1994) considered *X*. *axifera* and *X*. *meliacearum* collected from Puerto Rico to be specific to the petioles of the plant families Araliaceae and Meliaceae, respectively. However, collections of *X*. *meliacearum* from French Guiana and Venezuela seem to indicate a broader host range for the species. *Xylaria clusiae* and *X*. *pisoniae* were named after their hosts *Clusia* sp. (Samuels and Rogerson 1990) and *Eugenia capuli* (Rogers et al. 2001), respectively, but it is not yet clear if they are genuinely host-specific. Similar cases are found in the species epithets of *X*. *lindericola*, *X. hedyosmicola*, and *X. polysporicola*, which reflect the assumption that they prefer fallen leaves of *Lindera robusta*, *Hedyosmum orientale*, and *Polyspora hainanensis*, respectively (Pan et al. 2022).

As outlined in the dichotomous key above, the 45 species are separated into three groups based on stromatal shape, compactness of perithecial aggregation, and conspicuousness of perithecial mounds. Other diagnostic stromatal features include whether the stromatal apex is fertile or sterile, whether an outer stromatal layer persists into maturity as seen in *X*. *foliicola*, *X*. *minuscula*, *X*. *noduliformis*, *X*. *vittatipiliformis*, and *X*. *vittiformis*, and whether there are overlying spikes as in *X*. *asperata* or hairs as seen in *X*. *allima*, *X*. *castilloi*, *X*. *lima*, and *X*. *maitlandii*. Long and thread-like stromatal apices are noticeable and can be found in *X*. *filiformis*, *X*. *foliicola*, *X*. *minuscula*, *X*. *nainitalensis*, *X*. *sicula*, *X*. *simplicissima*, *X*. *vagans*, *X*. *vermiformis*, and *X*. *vittatipiliformis*. However, caution should be taken because the long stromatal apices can easily break off as seen in some herbarium specimens. For instance, the type material of *X*. *vagans* was described and illustrated as having a long, thin stromatal apex (Petch 1915) but is no longer as such. Fortunately, this feature has seldom been overlooked and can be retrieved by consulting the original descriptions and/or illustrations. Finely longitudinally striated stromatal surfaces that are often slightly polished can be found in *X*. *appendiculata*, *X*. *appendiculatoides*, *X*. *filiformis*, *X*. *polysporicola*, *X*. sp. AR1741, and *X*. *vagans*. This feature has diagnostic value and may eventually prove valuable in demonstrating the close relatedness of these species.

Ostioles can be slightly papillate, papillate, conic-papillate or coarsely conic-papillate as seen in species such as *X*. *appendiculata* and *X*. *allima*. The most salient ostioles is seen in *X*. *spiculaticlavata*, where the ostiolar bases are 270–310 μm broad, much broader than those of other species, which are less than 200 μm broad. Ostioles that tilt upwards conspicuously can be found in *X*. *appendiculatoides*, *X*. *allima*, *X*. *lima*, *X*. sp. AR1741, and *X*. *spiculaticlavata* and are useful for identification.

Ascospores are smooth-walled except for *X*. *simplicissima*, which has a lumpy appearance on the epispore along the germ slit. The hyaline sheath of ascospores is present in a half of the species, swelling at ascospore ends to form non-cellular appendages that are papillate in most species but tubular in *X*. *axifera* and cone-shaped in *X*. sp. AR1741. The ascospore germ slits are spore-length or nearly so in most species but much less than spore-length in *X*. *kamatii* (Pande 1973).

Cultures and/or anamorphs are known from approximately one-quarter of the leaf- and petiole-inhabiting *Xylaria* species. In the present study, cultures were obtained from five species: *X*. *amphithele*, *X*. *aristata* var. *aristata*, *X*. *minuscula*, *X*. *simplicissima*, and *X*. *vittatipiliformis*, but none of them produced anamorphs culture. Cultures have also been obtained from *X*. *axifera* (Læssøe and Lodge 1994), *X*. *castilloi* (San Martín et al. 1997), X. *hispidipes* (Rogers et al. 1987) (as *X*. *hypsipoda*), *X*. *meliacearum* (Læssøe and Lodge 1994), *X*. *phyllocharis* (San Martín et al. 1997)d *pisoniae* (Rogers et al. 2001). Among these species, *X*. *axifera* and *X*. *hispidipes* did not produce anamorphs in culture, but the anamorph of *X*. *axifera* was found on stromata collected from the field. The anamorphs of *X. castilloi*, *X*. *meliacearum*, and *X*. *pisoniae* were produced on the stromata produced in culture as well as collected from nature. Cultures have not been obtained from *X*. *asperata*, but its anamorph was reported as synnemata on the stromatal surface (Rogers et al. 1988). The anamorph of *X*. *lima* was reported by San Martín et al. (1997) (as *X*. *mexicana*) on the surface of immature stromata collected from the field.

## Conclusion

Most of the known leaf- and petiole-inhabiting *Xylaria* species are only represented by one or several dried herbarium specimens, indicating the necessity of recollecting them through further intensive collecting activities. DNA extractions from these herbarium specimens are normally unpermitted due to their status as type, historical or authentic specimens, or unsuitable because the specimens are scanty or moldy. Although teleomorphic characteristics are sufficient for recognizing 45 species, additional species may be revealed with data from cultures and/or anamorphs, which are currently quite limited and only available from approximately one-quarter of the species. Molecular phylogenetic studies, such as Hsieh et al. (2010) and Ren et al. (2016), where several leaf- and petiole-inhabiting *Xylaria* species were included, suggested the incoherence of these species, signifying the possibility of their multiple origins. Many more species are required to form a comprehensive molecular phylogenetic study on leaf- and petiole-inhabiting *Xylaria* species to reflect their phylogenetic relationships in taxonomy.

## Data Availability

Specimens collected by us are deposited at the herbarium HAST. Cultures are available at BCRC. ITS sequences are deposited at GenBank.
